# *In silico* and *in vitro* exploration of a tyrosinase for biocatalytic production of catechols

**DOI:** 10.1002/2211-5463.70311

**Published:** 2026-07-28

**Authors:** James Britton, Fang Zhao, Reeta Davis, Meg Walsh, Kevin E O'Connor, David O'Connell, Tanja Narancic

**Affiliations:** ^1^ BiOrbic Bioeconomy, Research Ireland Centre University College Dublin Ireland; ^2^ School of Biomolecular and Biomedical Science University College Dublin Ireland; ^3^ Nova Mentis Limited, Nova UCD, Belfield Innovation Park University College Dublin Ireland; ^4^ Present address: Research, Innovation & Engagement Office Atlantic Technological University Letterkenny Donegal Ireland; ^5^ Present address: Food Science Building, SUSFERM College of Science, Engineering & Food Science, University College Cork Cork County Cork Ireland

**Keywords:** AlphaFold, catechol, molecular docking, tyrosinase

## Abstract

Tyrosinases are binuclear copper enzymes widely used for the hydroxylation of phenolic compounds, yet most exhibit higher activity towards diphenols than monophenols, limiting their utility for catechol synthesis. However, a tyrosinase from *Ralstonia pseudosolanacearum* GMI1000 (RsTyr) is unusual in favouring monophenol substrates, making it a promising biocatalyst. Here, we combined molecular docking with kinetic characterisation to explore RsTyr's substrate range across eleven monophenols of industrial relevance. Docking studies using homology and AlphaFold models identified key interactions between substrate side chains and catalytic residues, particularly N228, N232 and P239. Experimental validation confirmed broad substrate acceptance, with highest catalytic efficiency observed for resveratrol and tyrosol. Structural analysis suggests that terminal functional groups and sidechain branching strongly influence activity. These findings provide mechanistic insight into RsTyr's substrate specificity and inform strategies for biocatalytic production of valuable catechols.

## Graphical abstract

Tyrosinase from *Ralstonia pseudosolanacearum* is a promising biocatalyst for producing valuable catechols from monophenol substrates. This tyrosinase is uniquely suited to this due to its high monophenolase : diphenolase ratio. We combined *in silico* docking and *in vivo* kinetic characterisation of this tyrosinase with 11 industrially relevant monophenols, identifying residues involved in catalysis and providing a roadmap to catechol production.

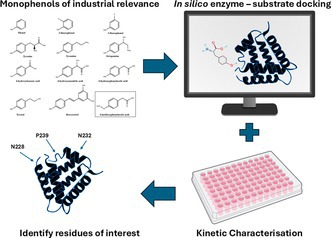

**Abbreviations**
2‐FP: 2‐fluorophenol
3‐FP: 3‐fluorophenol
4‐HBA: 4‐hydroxybenzoic acid
4‐HMA: 4‐hydroxymandelic acid
4‐HPAA: 4‐hydroxyphenylacetic acid
CSD: Cambridge structural database
CTD: C‐terminal domain
MDR: monophenolase: diphenolase ratio
PmTyr: *Priestia megaterium* tyrosinase
RsTyr: *Ralstonia pseudosolanacearum* tyrosinase

Catechols (diphenols) are valuable intermediates in the synthesis of antioxidants, pharmaceuticals, agrochemicals and flavour compounds, yet their production by organic chemistry synthesis remains challenging due to the difficulty of regiospecific hydroxylation of aromatic rings [3, 4, 5, 6, 7]. Due to their highly selective activity, oxidases, mono‐ and di‐oxygenases, native or engineered, have therefore found wide application in the synthesis of these catechols.

Tyrosinases (EC1.14.18.1) are a family of binuclear copper oxidase enzymes ubiquitous to all kingdoms of life that have been widely studied for this purpose [8]. In nature, tyrosinases catalyse the oxidation of both *o‐*monophenols and *o‐*diphenols, resulting in *o*‐quinone compounds which autopolymerise into pigmented melanins used for a variety of physiological purposes [9, 10, 11, 12, 13]. Due to their wide range of physiological functions, tyrosinases have evolved to have activity towards a wide range of both monophenol and diphenol compounds, and as such have garnered much attention as potential biocatalysts. They have been employed in the synthesis of *o‐*diphenols (catechols) [7, 14, 15, 16, 17], but also for use as biosensors for the detection of phenolic compounds or as bioremediation agents to remove phenolic compounds from the environment [18].

Past studies have shown tyrosinases can catalyse the conversion of monophenols and diphenols to their corresponding *o*‐quinones, which can be reduced to catechols (diphenols) under suitable conditions (Fig. 1) [7, 13, 19, 20]. However, most tyrosinases exhibit higher diphenolase activity than monophenolase activity, limiting their efficiency for monophenol biotransformations as they preferentially act on the desired diphenol product over the monophenol [21, 22].

**Fig. 1:**
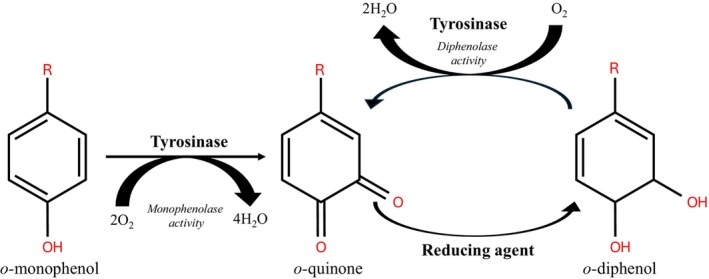
A tyrosinase catalysed biotransformation. The production of *o‐*diphenols (catechols) from *o‐*monophenol starting substrates using tyrosinase and reducing agents. Tyrosinases act on monophenols, converting them to quinone compounds. Maintaining a suitable reducing agent in the media allows for the reduction of the quinone to a catechol (*o‐*diphenol).

A small number of tyrosinases display an unusually high monophenolase:diphenolase ratio (MDR), including an enzyme from the plant pathogen bacterium *Ralstonia pseudosolanacearum* GMI1000 (UniProt ID: A0A0K1ZP03, henceforth RsTyr) [23]. This property makes RsTyr an attractive candidate for biocatalytic processes aimed at catechol synthesis. Despite its potential, detailed studies on RsTyr's substrate specificity and structural determinants of activity are lacking.

Here, we investigate RsTyr's substrate range using a combined *in silico* and *in vitro* approach. Molecular docking was performed on eleven monophenolic substrates of industrial relevance (Fig. 2, Table 1), followed by kinetic characterisation of selected compounds. Our findings reveal how sidechain branching and terminal functional groups influence substrate orientation and catalytic efficiency, providing mechanistic insight and guiding future bioprocess development for catechol production.

**Fig. 2:**
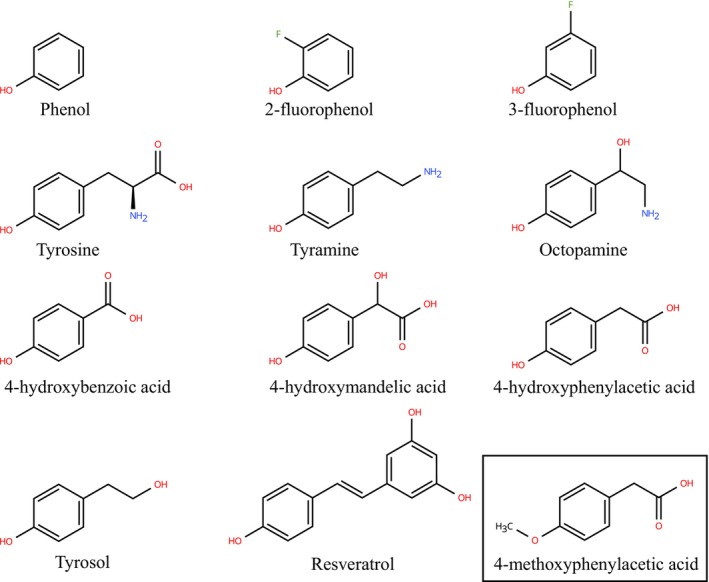
Structures of compounds tested against the *R. pseudosolanacearum* tyrosinase. In the black box is 4‐methoxyphenylacetic acid, which was used as a negative control.

**Table 1:** A list of compounds docked to RsTyr models, their desired catechol products and the uses of these products.

Substrate	Target product	Product use(s)	References
L‐tyrosine	L‐DOPA	Parkinson's disease treatment.	[24]
Tyramine	Dopamine	Neuromodulatory catecholamine used for treatment of low blood pressure in children.	[25]
Octopamine	Norepinephrine	A neurotransmitter and hormone used for treatment of low blood pressure in adults.	[26]
Phenol	Catechol	Chemical precursor for fragrances, flavourings and pesticides.	[3]
2‐fluorophenol (2‐FP)	2‐fluorocatechol	Potential precursor for biogenic amines and adrenergic catecholamines.	[27]
3‐fluorophenol (3‐FP)	3‐fluorocatechol	Potential precursor for biogenic amines and adrenergic catecholamines.	[27]
Tyrosol	Hydroxytyrosol	Potent phenolic antioxidant with cardiovascular and LDL‐cholesterol related health benefits.	[28]
Resveratrol	Piceatannol	Polyphenolic stilbene antioxidant with anticarcinogenic properties.	[29]
4‐hydroxybenzoic acid (4‐HBA)	Protocatechuic acid	A plant polyphenol antioxidant associated with reducing risk factors for several diseases.	[30]
4‐hydroxymandelic acid (4‐HMA)	3,4‐dihydroxymandelic acid	Potent phenolic antioxidant and a metabolite of norepinephrine	[31]
4‐hydroxyphenylacetic acid (4‐HPAA)	3,4‐dihydroxyphenylacetic acid	Potent phenolic antioxidant with protective effects against mitochondrial dysfunction, oxidative stress and apoptosis	[32]

## Materials and methods

### Computational studies

#### Substrate preparation

Chemical structures (.mol2 files) for all substrates were obtained from ChemSpider and optimised using the Cambridge Structural Database (CSD) conformer generator via Mercury (v2024.1.0) [33]. Ten conformers were generated per substrate.

#### Protein model generation

The RsTyr crystal structure (PDB: 7XIO) was retrieved from RCSB PDB [34]. Missing flexible loop regions were modelled using SWISS‐MODEL (ProMod3 engine) [35, 36], and copper ions were added into the active site using the MIB2 metal‐binding prediction server [37]. To retain water molecules present in the crystal structure, a merged model (7XIO‐II.pdb) was created in YASARA [38], combining the homology model with the water molecules present in the original crystal structure. Additional models were generated using AlphaFold 3 [39], and with SWISS‐MODEL using the list of *Priestia megaterium* (PmTyr) tyrosinase crystal structures in Table S1 as templates [35, 36]. Structural alignments were performed in PyMOL (v2.4.1) [40]. A list of RsTyr model structures generated during this study can be found in Table S2 [41]. PDB files for all models used in this study are freely available on Zenodo [41].

#### Docking protocol

Enzyme‐Substrate Docking was carried out using CSD GOLD with ChemPLP scoring through the Hermes GUI (v2024.1.0) [33]. The initial docking protocol was refined by ensemble redocking of tyrosine and tyrosol into PmTyr crystal structures listed in Table S1 (Figs S1–S4, [41]) with 100 separate docking experiments [42]. In the final protocol, the binding site was defined as a 20 Å sphere around copper ion A (CuA). Each ligand underwent 10 docking runs per conformer. Analysis of all crystal structures of PmTyr (listed in Table S1) noted movement of residues D55, E158, M184 and R209. As such, these residues were allowed flexibility during docking. Docking validation was performed by comparing the top docked tyrosine and tyrosol solution orientations to orientations of these substrates in PmTyr crystal structures (4P6R and 4P6T, Table S3, [41]).

For RsTyr, residues Y88, N184, M204 and N232 were set as flexible based on their analogous position to the residues listed above identified in PmTyr crystal structures (listed in Table S1) [42]. For docking using SWISS‐MODEL generated structures, an ensemble docking strategy was employed as above. Substrates were docked into AlphaFold generated structures using a single structure rather than an ensemble strategy, with all other parameters kept the same.

Docking poses were analysed in PyMOL, and interaction data were compiled for hydrogen bonds, halogen bonds, copper ion–substrate distance and *π*–*π* interactions. A list of all docking files can be found in Table S4; PDB files for top scoring docking results are available on Zenodo [41].

### Expression and purification of RsTyr

*E. coli* BL21 was used to express *R. pseudosolanacearum* tyrosinase (RsTyr) from a pRSETb vector constructed previously [43]. Strains were maintained in lysogeny broth (LB) with 25% glycerol at −80 °C. In preparation for tyrosinase expression *E. coli* strains stored at −80 °C were streaked on LB agar and incubated at 37 °C overnight. A primary inoculum cultured from a single colony from an agar plate was grown in 2 mL of LB overnight in a rotary shaking incubator (200 rpm, 37 °C). Carbenicillin antibiotic was used for maintenance of plasmids throughout the study at a concentration of 50 μg·mL^−1^. 0.5 mL of the primary inoculum was added to 49.5 mL of LB broth in 250 mL culture flasks which were then incubated at 37 °C, 200 rpm in a shaking incubator until an OD_600_ of 0.4 was reached. At this point flasks were incubated at 4 °C for 15 min followed by the addition of induction agent isopropyl‐β‐d‐1‐thiogalactopyranoside to a final concentration of 1 mm. The flasks were then incubated at 30 °C, 200 rpm in a shaking incubator for a further 16 h. At this point cells were harvested by centrifugation at 4 °C, 7000 × ***g*** for 10 min.

Cells harvested from tyrosinase expression culture were lysed to create a crude whole cell homogenate. To create whole cell homogenate, cell pellets were resuspended in 5 mL 50 mm phosphate buffer pH 7, 1 μL·mL^−1^ homogenate of protease inhibitor cocktail (Sigma‐Aldrich) and 1 μL·mL^−1^ of a 1 mg·mL^−1^ DNase solution (Sigma‐Aldrich) per gram cell wet weight. Resuspended cells were lysed via French press homogenisation at 12 000 PSI. The resulting homogenate was collected and tyrosinase purified as described previously [43].

### Activity screening and kinetic assays

Initial screening was performed by incubating 2 μg purified RsTyr with 1 mm substrate in 50 mm phosphate buffer (pH 7.0) at 30 °C overnight. Quinone formation was assessed visually and by UV–Vis spectroscopy (250–500 nm). The peak absorbance (*λ* max) and molar extinction coefficient of these quinone compounds were sourced from the literature where possible. Peak absorption and molar extinction coefficients for the substituted quinone compounds formed by resveratrol and 4‐hydroxymandelic acid (4‐HMA) were determined here as previously described (Fig. S5A–E) using HPLC to monitor substrate depletion [44].

Kinetic analyses were performed as described by Nolan et al. [44]. Reactions (200 μL) contained 50 mm phosphate buffer, substrate (0.01–50 mm) and 2 μg purified RsTyr. Initial rates were measured spectrophotometrically using the peak absorption spectra and molar extinction coefficient for the relevant 4‐substituted quinone compound formed by the substrate in question. Kinetic parameters (*K*_m_ (mm), *k*_cat_ (min^−1^), *k*_cat_ (min^−1^)/*K*_m_ (mm)) were calculated using enzfitter software [45].

## Results

### Structural modelling and docking

Sequence analysis of RsTyr by InterPro (https://www.ebi.ac.uk/interpro/) [1] confirmed RsTyr as an enzyme within the phenol oxidase family and identified three sub‐domains within the protein: an N‐terminal twin arginine translocase (TAT) signal peptide (M1–A34), a polyphenol oxidase catalytic domain (R38–V321) and a polyphenol oxidase C‐terminal domain (P363–I496) (Fig. S6, [1]).

At the time of writing one crystal structure for RsTyr has been released (PDB: 7XIO, [46]), however it is incomplete and lacks the canonical two copper (Cu) ions in the catalytic centre, which resulted in poor docking performance. As described above, SWISS‐MODEL was used to generate a model using 7XIO as a template to generate missing segments of the crystal structure (T335–L347, A372–T389 and T435–H443), all within flexible loops. The Cu ions were added to this generated model using the top predictions made by the MIB2 metal ion binding prediction webserver [37].

Docking using the modified 7XIO structure led to negative ChemPLP fitness scores under all docking protocols tested (Table S5). Additionally, tyrosine and tyrosol docked into 7XIO were oriented with the R‐group of the substrates in contact with the catalytic Cu ions, in contrast to what has been previously observed in PmTyr crystal structures (Figs S7, S8 [46]).

As such, a library of homology models was generated via SWISS‐MODEL and an AlphaFold 3 [39] prediction was also performed (Table S2, [41]). All models displayed two Cu ions bonded to the six conserved histidines in the active site in an orientation like that observed in crystal structures for other tyrosinases. Alignment revealed that the AlphaFold model was closest to the crystal structure (RMSD = 0.387 Å).

As with 7XIO, docking attempts using the AlphaFold model structure prediction which modelled the entirety of RsTyr (Residues 1–496) were unsuccessful (Figs S9, S10, [46]). It was hypothesised that the C‐terminal domain (CTD) of RsTyr was retarding successful docking by impeding substrate access to the active site. CTD removal or movement to an altered confirmation is commonly seen in plant and fungal tyrosinases prior to catalytic activity [47, 48]. As such, all docking studies from this point forth used structure models of RsTyr which contained only the catalytic domain (residues R38 – V321).

**Fig. 3:**
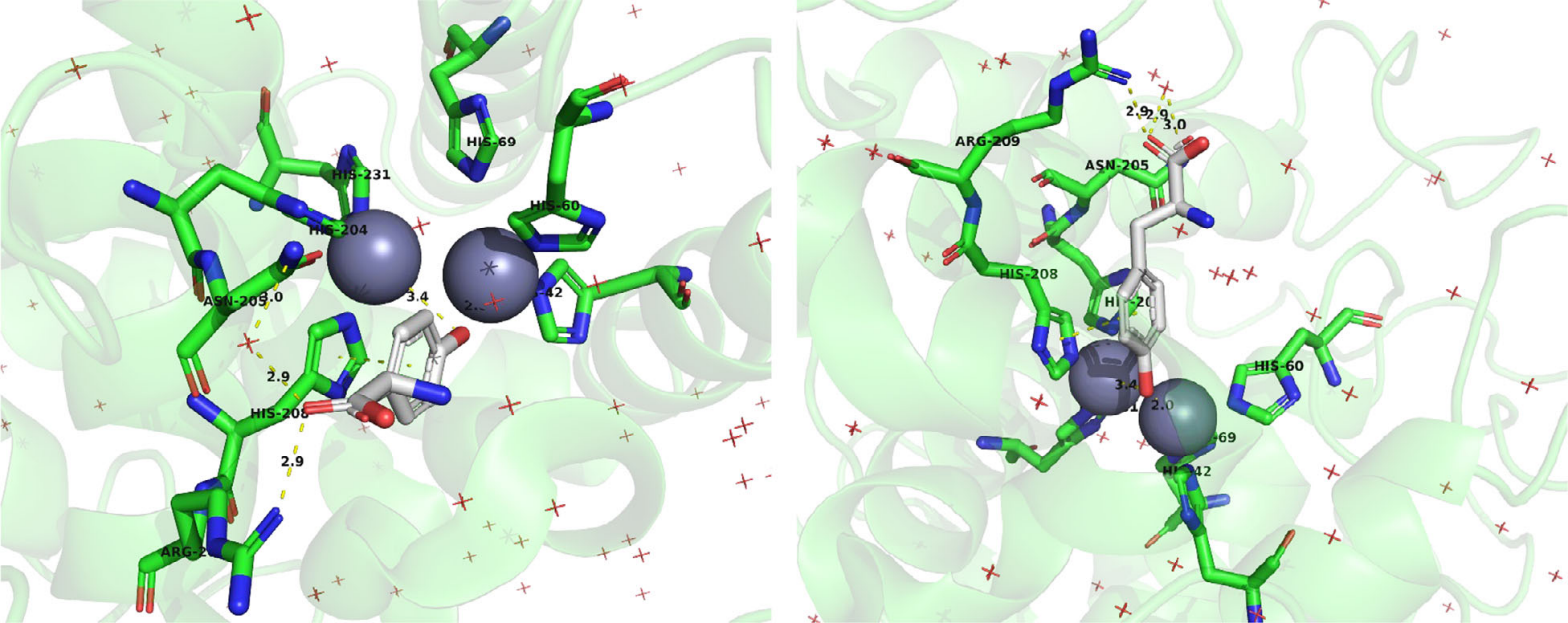
Crystal structure 4P6R.pdb showing tyrosine bound to the active site of *Priestia megaterium* tyrosinase (PmTyr). The protein backbone is shown as a green ribbon; substrates are shown as grey stick models; cofactors are shown as spheres of brown (copper) or grey (zinc). The six copper‐coordinating histidine residues and the two activity controller residues are highlighted as green stick models. Additional residues with which substrates interact are also highlighted as green stick models. Water molecules are shown as red stars. PDB files for all docking solutions shown here are freely available on Zenodo [41].

**Fig. 4:**
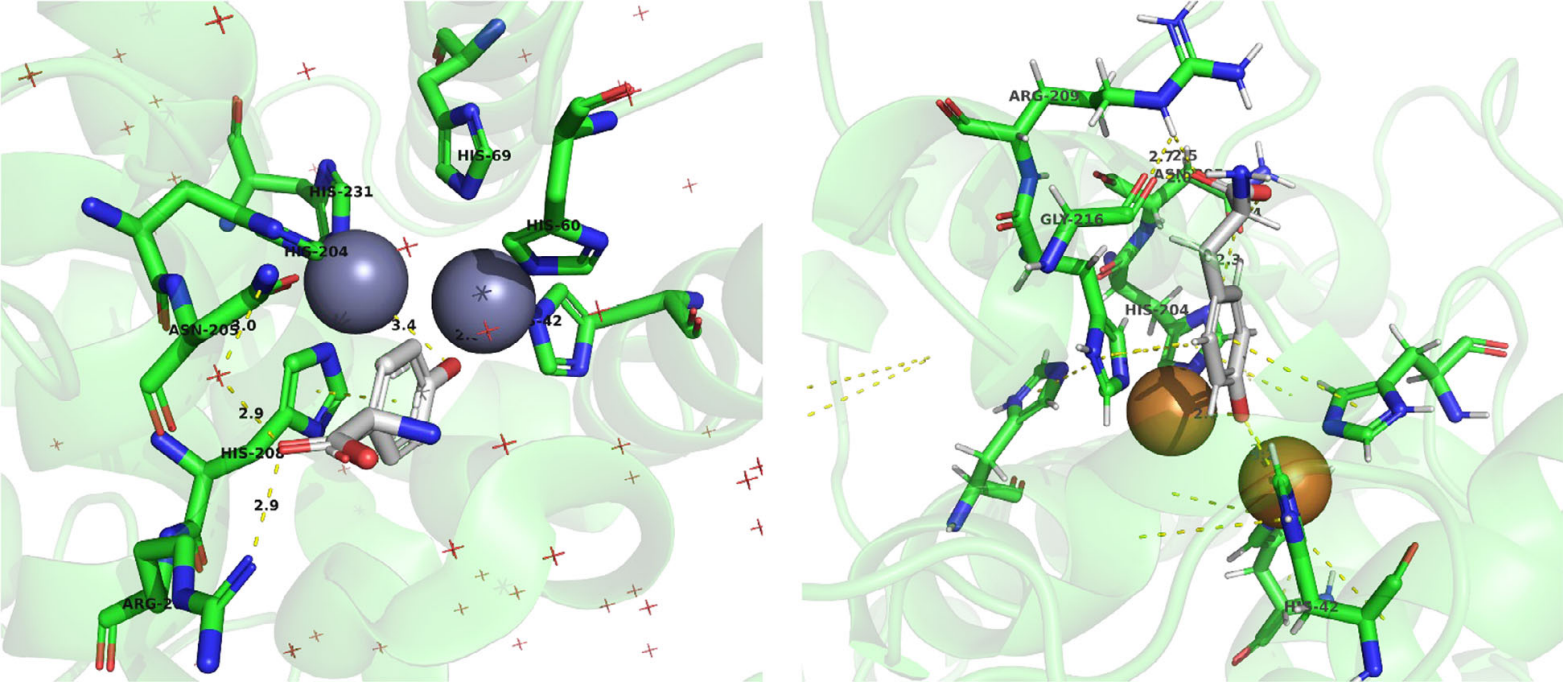
Top scoring result of ensemble redocking of tyrosine into *Priestia megaterium* tyrosinase crystal structures. The protein backbone is shown as a green ribbon, substrates are shown as grey stick models, cofactors are shown as spheres of brown (copper) or grey (zinc). The six copper‐coordinating histidine residues and the two activity controller residues are highlighted as green stick models. Additional residues with which substrates interact are also highlighted as green stick models. Water molecules are shown as red stars. PDB files for all docking solutions shown here are freely available on Zenodo [41].

**Fig. 5:**
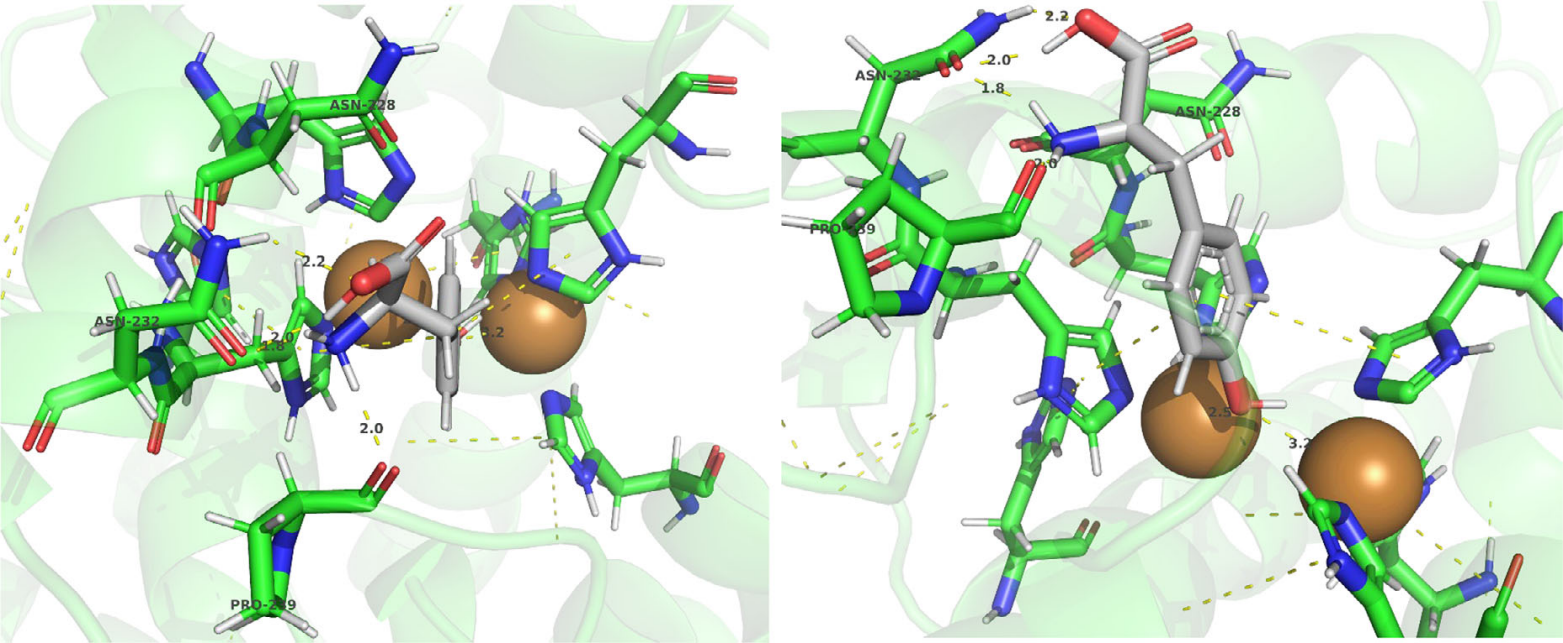
Top scoring result of ensemble docking of tyrosine into SWISS‐MODEL homology structures prediction of RsTyr catalytic domains. The protein backbone is shown as a green ribbon, substrates are shown as grey stick models, cofactors are shown as spheres of brown (copper) or grey (zinc). The six copper‐coordinating histidine residues and the two activity controller residues are highlighted as green stick models. Additional residues with which substrates interact are also highlighted as green stick models. Water molecules are shown as red stars. PDB files for all docking solutions shown here are freely available on Zenodo [41].

**Fig. 6:**
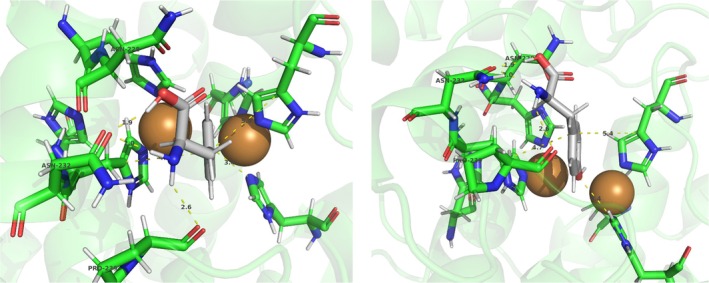
Docking of tyrosine into AlphaFold structure prediction of RsTyr catalytic domain (RsTyr_catalytic_AF3.pdb). Structures were predicted by SWISS‐MODEL or AlphaFold as indicated in each subheading. Crystal structures resolved by Goldfeder et al. [42] were used where indicated in each subheading above. In all images, the protein backbone is shown as a green ribbon, substrates are shown as grey stick models, cofactors are shown as spheres of brown (copper) or grey (zinc). The six copper‐coordinating histidine residues and the two activity controller residues are highlighted as green stick models. Additional residues with which substrates interact are also highlighted as green stick models. Water molecules are shown as red stars. PDB files for all docking solutions shown here are freely available on Zenodo [41].

Ensemble docking of tyrosine into a library of RsTyr SWISS‐MODEL homology structures modelled on the panel of PmTyr crystal structures listed in Table S1 [41] led to substrate orientations similar to those observed when tyrosine was observed crystallised in tyrosinase from *P. megaterium* (4P6R) (Figs 3, 4, 5) [42]. As such a new AlphaFold 3 model of RsTyr was generated, modelling only the catalytic domain of RsTyr, docking validation with this model led to tyrosine docked in a similar orientation to that of crystal structure 4P6R (Fig. 6).

Docking of the twelve compounds listed in Table 1 was then carried out using SWISS‐MODEL and AlphaFold 3 models of the RsTyr catalytic domain. A table detailing ChemPLP fitness scoring for each substrate can be found in the Supporting Information (Table S5). Images of the top scoring docking solutions for each substrate with both SWISS‐MODEL and AlphaFold 3.0 structures can be found in the Supporting Information (Figs S11–S22, [49] and Figs S23–S34, [39] respectively). PDB files of the top scoring docking solutions for each substrate with both AlphaFold 3.0 and SWISS‐MODEL structure predictions are freely available on Zenodo [41].

### Enzyme–substrate interaction patterns

Docking of the chosen monophenolic substrates to both AlphaFold and SWISS‐MODEL structure predictions revealed consistent *π*–*π* interactions with active site histidines (H93 and H231) and variable hydrogen bonding with Y88, N228, N232 and P239 (Table 2). Halogen bonding with H93, H227, N228, T240 and S243 was observed between RsTyr and the fluorine groups of 2‐ and 3‐fluorophenol. Docking of all substrates bar 2‐fluorophenol (during SWISS‐MODEL structure docking) and 4‐methoxyphenylacetic acid showed the phenol hydroxyl group oriented towards the catalytic copper ions at distances ranging from 2.4 Å to 3.5 Å (Table 2).

**Table 2:** Hydrogen bonds, halogen bonds, *π*–*π* interactions and substrate phenol group–copper ion distances displayed by docking of substrates to SWISS‐MODEL and AlphaFold models of RsTyr. PDB files of the top docking solutions for each substrate with both docking strategies are freely available on Zenodo [41].

Substrate	Hydrogen bonds		π–π interactions		Phenol group–CuA/CuB distance (Å)	
	SWISS‐MODEL	AlphaFold 3	SWISS‐MODEL	AlphaFold 3	SWISS‐MODEL	AlphaFold 3
L‐tyrosine	N232, P239	N232, P239	H93, H231	H93, H231	3.2/2.5	2.6/2.6
Octopamine	N228, N232, P239	Y88, N232	H93, H231	H93, H231	3.4/3.2	2.4/2.6
Tyramine	N228, N232	N232, P239	H93, H231	H93, H231	3.3/2.6	2.5/2.6
L‐tyrosol	N228, N232	N232	H93, H231	H93, H231	3.2/2.7	2.4/2.6
Resveratrol	N/A	N232	H93, H231	N/A	3.4/2.7	2.4/2.6
Phenol	N/A	N/A	H93, H231	H93, H231	3.3/2.5	2.5/2.6
2‐FP	T240, S243, H93 (Halogen bond)	H227 (Halogen bond)	H93, H231	H93, H231	6.4/5.2	2.6/2.6
3‐FP	N228 (Halogen bond)	N228 (Halogen bond)	H93	N/A	3.3/2.5	2.6/2.6
4‐HBA	N232	N232, P239	H93, H231	H93, H231	3.5/3.5	2.6/3.2
4‐HMA	N232, P239	Y88, N232, P239	H93, H231	H93	3.3/2.6	2.4/2.6
4‐HPAA	N232, P239	N232	H93, H231	H93, H231	3.3/2.6	2.5/2.6

N232 was observed interacting with all substrates bar phenol and the fluorophenols. Similarly N228 was observed interacting with side chains of tyrosine, tyramine, octopamine, 4‐HBA, 4‐HMA and 4‐HPAA.

Interactions with Y88 were observed with the branched side chains of octopamine and 4‐HMA only when using the AlphaFold model structure; interestingly, in the corresponding docking using SWISS‐MODEL structures, interaction with P239 took the place of Y88. This may be due to the different orientation of Y88 between the AlphaFold and SWISS‐MODEL structures. P239 was also predicted to form hydrogen bonds with tyrosine, tyramine, 4‐HBA and 4‐HPAA. Interactions with Y88 and P239 are likely stabilising the side chains of the various substrates.

#### Interactions with the ‘activity controller’ residues N228 and N232

N232 together with N228 is the two ‘activity controller’ residues of RsTyr, identifiable by their position immediately downstream of the first two Histidines coordinating the binding of copper ion B. Also known as HisB1^+1^ and HisB2^+1^, the activity controllers have been identified as having a critical influence on tyrosinase catalysis through facilitating substrate deprotonation and subsequently substrate orientation in the active site [13, 50].

Apart from phenol and the fluorophenols, all substrates were predicted to interact with the second activity controller N232. Previous studies showed *P. megaterium* tyrosinase (PmTyr) crystallised bound with tyrosine and tyrosol displayed hydrogen bonding between the second activity controller (R209 in PmTyr) and the substrates [42], directly supporting the efficacy of the *in silico* docking performed here. However, compared to PmTyr, RsTyr has a significantly higher monophenolase: diphenolase activity ratio (RsTyr MDR = 5.4, PmTyr MDR = 0.09) [21, 23]. Past studies have shown that, to facilitate catalysis, asparagine is the most beneficial residue in the activity controller positions [22], and the residues in these positions may be key activity determinants between tyrosinases and catechol oxidases [50, 51]. This docking study indicates N232 is the dominant determinant of binding across substrates containing protruding sidechains. Engagement occurs either via the N232 terminal oxygen (tyrosine, tyramine, octopamine) or via its terminal amine, depending on the ligand's terminal functional group and sidechain.

In contrast with N232, predicted interactions with N228 were more varied and model dependent. N228 engaged tyramine, octopamine and tyrosol during docking with the SWISS‐MODEL structure. However, N228 was replaced by P239 (tyramine docking), Y88 (octopamine docking) and a second N232 bond (tyrosol docking) in AlphaFold structure docking solutions.

#### P239 the ‘gatekeeper’ candidate

P239 is positioned in a flexible loop directly above the active site. Docking showed recurrent contact by amine‐ and carboxylate‐terminated ligands. Its positioning appears to ‘brace’ the side chain, potentially stabilising the phenolic ring over the dicopper centre. Model‐dependent differences in P239 proximity (SWISS‐MODEL vs. AlphaFold) likely contribute to the slightly different contact maps noted for several substrates. Other studies have reported the existence of a ‘gatekeeper’ residue, present in analogous flexible loops above the active sites of other tyrosinases [13, 21, 42]. The so‐called gatekeeper is reported to influence substrate orientation and therefore catalysis; we purport that P239 may act as the gatekeeper residue in RsTyr.

#### Y88

Y88 was a recurrent acceptor for branched hydroxyl groups such as octopamine and 4‐HMA in AlphaFold models (Figs S26, S32). Model‐dependent differences in the orientation of Y88 prevented interaction between this residue and substrates in SWISS‐MODEL docking (Figs S14, S20).

#### Halogen bonding

2‐fluorophenol had a significantly different interaction profile than any other substrate tested, forming halogen bonds with T240, S243 and with the copper‐coordinating H227 in SWISS‐MODEL and AlphaFold structure docking respectively (Table 2, Figs 4, S17, S29). Additionally, during SWISS‐MODEL structure docking the hydroxyl phenol group of 2‐fluorophenol was positioned much further away from the copper ions than the other substrates (Table 2).

**Fig. 7:**
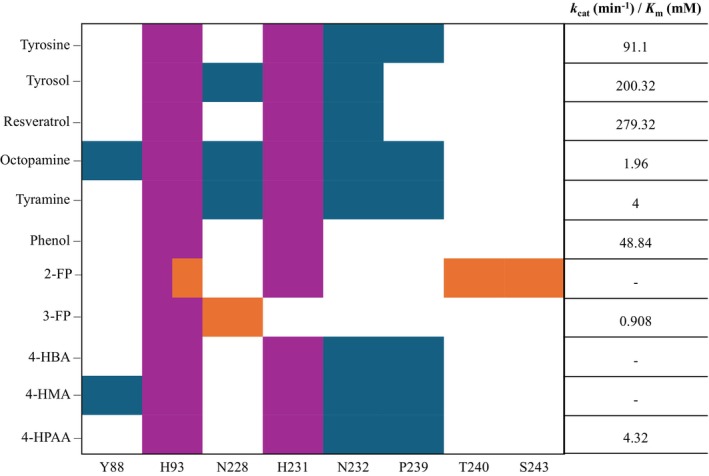
A heatmap of residue interactions formed during docking of tested substrates into RsTyr and their respective catalytic efficiency (*k*_cat_ (min^−1^)/*K*m (mm)). Colours indicate the type of bonding predicted in docking studies. Blue indicates hydrogen bonding, pink indicates π–π interactions, orange indicates halogen bonding.

Analysis of 3‐fluorophenol docking poses shows consistent halogen bond prediction between the fluorine group and N228 (Table 2). It is of note that past studies have shown phenolic compounds with electronegative sidechains such as those containing fluorine are poor tyrosinase substrates [52]. While the predicted halogen bonding of H93, T240 and S243 may retard catalysis of 2‐fluorophenol, halogen bonding between 3‐fluorophenol and N228 potentially interferes with the residue's function as an ‘activity controller’ [13], interfering with catalysis but not eliminating it completely.

### *In vitro* activity screening

Screening for RsTyr activity towards substrates listed in Table 1 revealed pigment formation on all substrates except 2‐fluorophenol and the negative control (4‐methoxyphenylacetic acid) (Table 3).

**Table 3:** A colorimetric tyrosinase activity screen towards selected substrates. Tyrosinase catalysis of monophenol substrates will yield pigmented quinone compounds.

Substrate	Colour formation?	Substrate	Colour formation?
L‐tyrosine	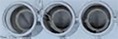	2‐fluorophenol	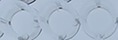
L‐tyrosol	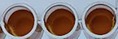	3‐fluorophenol	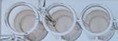
Resveratrol	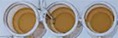	4‐hydroxybenzoic acid	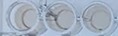
Octopamine	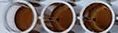	4‐hydroxymandelic acid	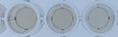
Tyramine	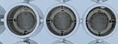	4‐hydroxyphenylacetic acid	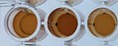
Phenol	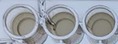	4‐methoxyphenylacetic acid	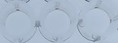

### Kinetic characterisation

Of the ten substrates which showed pigment formation during the activity screen (Table 3) RsTyr activity towards eight of these was kinetically characterised (Table 4). The molar extinction coefficient of the substituted quinone product formed by RsTyr catalysis of 4‐HBA could not be determined (Fig. S5C, [44]). Kinetic parameters of RsTyr activity towards 4‐HMA were not characterised as absorbance change over the course of the assay was minor and appeared subject to substrate inhibition above 30 mm (Fig. S35).

**Table 4:** Kinetic data of RsTyr towards monophenol substrates. 4‐hydroxymandelic acid has been removed as the change in absorbance over the assay period was too low for kinetic data calculation (Fig. S35). All values are an average of three independent experiments.

Substrate	*K*_m_ (mm)	*k*_cat_ (min^−1^)	*k*_cat_ (min^−1^)/*K*_m_ (mm)
L‐tyrosine	0.68 ± 0.13	62 ± 14.92	91.1
L‐tyrosol	0.83 ± 0.14	165.5 ± 29.7	200.32
Resveratrol	0.497 ± 0.11	138.83 ± 24.11	279.26
Octopamine	5.15 ± 0.375	10.13 ± 2.49	1.96
Tyramine	7.14 ± 1.25	28.55 ± 12.25	4
Phenol	2.13 ± 0.07	104.03 ± 21.39	48.84
3‐fluorophenol	368.72 ± 0.57	334.83 ± 12.99	0.908
4‐hydroxyphenylacetic acid	2.53 ± 1.38	9.99 ± 4.32	4.32

RsTyr exhibited highest catalytic efficiency towards resveratrol (*k*_cat_/*K*_m_ (min^−1^) = 279.3) and tyrosol (200.3), followed by tyrosine (91.1). The turnover number of RsTyr towards phenol was quite high (*k*_cat_ (min^−1^) = 104.03), however it displayed poor affinity (*K*_m_ = 2.13) relative to tyrosine, tyrosol or resveratrol. RsTyr showed weaker activity towards tyramine, octopamine and 4‐HPPA, each with a comparatively low turnover number and poor affinity.

RsTyr displayed poor affinity towards 3‐fluorophenol (*K*_m_ > 300 mm) despite a high turnover number, indicating a potentially unfavourable binding orientation.

### Correlation between docking and catalysis

*In vitro* kinetic data was compared with *in silico* docking to examine substrate binding and catalysis. The goal was to relate residue‐level interactions in the active site to observed differences in affinity (*K*_m_) and turnover (*k*_cat_) across chemically related substrates. Because tyrosinases employ a dicopper active site to perform phenolic ortho‐hydroxylation and subsequent oxidation, the productive orientation of the phenolic hydroxyl relative to the copper centre is expected to be a determinant of catalysis. We therefore focused on (i) residues that contact the side chain and influence pose pre‐organisation, and (ii) positional effects of substituents that may reorient the phenolic OH away from the copper ions.

Of the 11 potential substrates tested, all bar 2‐fluorophenol showed colour development during the screening phase (Table 3). Figure 7 illustrates the interactions between RsTyr and the tested substrates compared with the measured catalytic efficiency.

### Substrate‐class analyses

#### Aminated substrates: Tyrosine, tyramine, Octopamine

All three substrates form a conserved interaction where the terminal amine engages the terminal oxygen of N232 in docking with both model types (Figs S5 and S6). This anchoring likely positions the phenolic OH towards the copper centre.

##### Model‐dependent contacts

- Tyrosine: Both docking strategies maintain the N232 contact; additional P239 and/or N228 interactions are variably present and likely reflect local differences in flexible loop conformations between AlphaFold and SWISS‐MODEL structures (Figs S11, S23).
- Tyramine: Both docking strategies maintained contact with N232. SWISS‐MODEL docking predicts N228 engagement while AlphaFold docking favours P239 (Figs S13, S25).
- Octopamine: Both docking strategies maintained contact with N232. SWISS‐MODEL docking shows N228 engagement via the branched β‐OH; AlphaFold docking substitutes this with Y88 (Figs S14, S26).

Tyramine and octopamine have 7.5–10× higher *K*_m_ (mm) than tyrosine (Table 4). Despite similar anchoring at N232, *k*_cat_ differs markedly: tyramine turnover is ~3× faster than octopamine (Table 4), possibly because the β‐OH in octopamine forms additional H‐bonds (e.g., to Y88) trapping it in a suboptimal orientation for catalysis. The contact with N228 observed in docking of the SWISS‐MODEL structure and octopamine via the branched β‐OH seems analogous to the contact formed with Y88 when octopamine was docked to the AlphaFold structure.

#### Tyrosol and resveratrol

In the AlphaFold model, resveratrol forms a single H‐bond to N232 (Fig. S27); meanwhile, SWISS‐MODEL docking did not predict side‐chain contact (Table 4, Fig. S15). Despite sparse polar contacts, RsTyr displays excellent efficiency towards resveratrol with a comparable *K*_m_ and *k*_cat_ > 2× that of tyrosine. The extended, planar scaffold possibly supports further favourable π‐stacking or hydrophobic complementarity that, together with N232 anchoring shown during docking with the AlphaFold model, suffices for high turnover.

For tyrosol in contrast both models predict two H‐bonds from the sidechain's terminal hydroxyl, but with different partners depending on the model used:

- SWISS‐MODEL ensemble docking: Tyrosol terminal hydroxyl binds N232 amine and N228's carbonyl oxygen (Fig. S12).
- AlphaFold structure docking: Tyrosol terminal hydroxyl binds N232 amine and N232 terminal oxygen (Fig. S24).

#### Carboxylates: 4‐HBA, 4‐HMA, 4‐HPAA

In kinetic assays, 4‐HBA showed no measurable activity; activity towards 4‐HMA was weak and seemed to suffer substrate inhibition (Fig. S35); 4‐HPAA exhibited an overall efficiency comparable to tyramine (Table 4).

Across models, each carboxylate forms an H‐bond between a carboxylate oxygen and the N232 amine (Table 2, Figs 4, S19–S21, S31–S33). P239 contacts are common but model‐dependent (exceptions: 4‐HBA in SWISS‐MODEL; 4‐HPAA in AlphaFold).

##### Model‐dependent poses

- 4‐HBA: The SWISS‐MODEL docking strategy favoured interaction with the N232 terminal oxygen, whereas AlphaFold docking model favours interaction with the N232 terminal nitrogen plus a stabilising second contact to P239 (Figs. S19, S31).
- 4‐HMA: The branched β‐OH of the substrate engages P239 (SWISS‐MODEL) or Y88 (AlphaFold), paralleling the branched β‐OH of octopamine (Figs. S20, S32).
- 4‐HPAA: A carboxylate oxygen interacts with both the N232 amine and an oxygen of P239 during docking using the SWISS‐MODEL structure. Meanwhile, two H‐bonds are formed between the substrates' carboxylate oxygen and N232 (amine and oxygen) during docking with the AlphaFold structure (Figs S21, S33).

The single β‐OH substituent (present in 4‐HMA vs. 4‐HPAA, and in octopamine vs. tyramine) consistently correlates with poorer catalysis, suggesting that this group tends to form non‐productive H‐bonds within the pocket as witnessed by docking studies, raising *K*_*m*_ and/or reducing *k*_cat_. The absence of measurable activity for 4‐HBA may result from its carboxylate occupying the same spatial region used by the β‐OH in octopamine/4‐HMA.

#### Phenol and fluorophenols

RsTyr showed no activity towards 2‐FP in the activity screen (Table 3). Docking to the SWISS‐MODEL structure predicts H‐bonds from the phenolic OH to T240 and S243 and a halogen bond from fluorine to H93 (Table 2, Figs 4, S17). Meanwhile, docking to the AlphaFold model predicts a halogen bond to H227 (Table 2, Figs 4, S29). In both cases, the ring is anchored such that the phenolic OH is misoriented relative to the copper site, consistent with the observed lack of activity and with prior reports of inhibitory behaviour for 2‐substituted phenols [52].

RsTyr showed low activity towards 3‐FP (Table 4). 3‐fluorophenol docking to both models predicted halogen bond formation from the substrate fluorine to the terminal oxygen of N228 (Table 2, Figs 4, S18, S30). Relative to 2‐FP, this interaction seems to be less disruptive to the orientation of the phenolic OH. For context, past studies have demonstrated RsTyr retains significant activity towards 4‐fluorophenol (*k*_cat_ = 618 ± 22 min^−1^; *K*_m_ = 34 ± 2 mm) [16]. Halogen bonding to N228 may interfere with its role as the first ‘activity controller’ residue and so negatively impact catalysis.

There were only minor differences in the orientation of phenol docked into both SWISS‐MODEL and AlphaFold model structures (Figs 4, S16, S28). As with the other substrates, π‐π interactions between the substrates' aromatic ring and H93 and H231 of RsTyr were present (Table 2). The *k*_cat_ of RsTyr towards phenol was in a similar range to those of tyrosine, tyrosol and resveratrol (*k*_cat_ (min^−1^) = 104.03); however, the *K*_m_ observed towards phenol (*K*_m_ (mm) = 2.13) was between 2.5 to 4‐fold higher than these substrates. This could possibly be explained by the lack of an anchoring sidechain in phenol.

### Comparative structure–activity themes

#### Substrate terminal group: Tyrosol vs. Tyramine vs. 4‐hydroxyphenylacetic acid

These substrates differ only at the terminal group (alcohol vs. amine vs. carboxylate) yet elicit distinct activities (Table 4). The common denominator between the three substrates is N232 engagement coupled with orientation of the phenolic OH towards copper. Tyrosol benefits from dual N232 contacts (AlphaFold, Fig. S24) and an uncharged, compact terminus, aligning with low *K*_m_ and high *k*_cat_. Docking of both tyramine and 4‐HPAA displays interaction between the sidechains' terminal group and P239 (Figs S13, S21, S25, S33); this may account for the higher *K*_m_ and lower *k*_cat_ observed.

#### Effect of branching on the β‐carbon

Across both amine and carboxylate series, β‐OH branching (octopamine, 4‐HMA) correlates with reduced turnover and/or higher *K*_m_ relative to the unbranched analogues (tyramine, 4‐HPAA) (Fig. 3). The mechanistic picture is that the β‐OH often ‘finds’ a hydrogen‐bond partner e.g. Y88 (Figs S26, S32) or P239 (Figs S14, S20) that stabilises a non‐productive orientation, good enough to bind (thus not necessarily catastrophic for *K*_m_), but misaligned for catalysis (depressing *k*_cat_).

### Working mechanistic model

RsTyr catalytic performance seems to be governed primarily by N232‐mediated anchoring of the substrate side chain, with P239 likely serving as the ‘gatekeeper’ which aids in stabilising the aromatic ring above the dicopper centre. N228 interaction can contribute to binding but does not seem critical to catalysis. Y88/P239 were observed capturing β‐OH branches, correlating with reduced *k*_cat_ (octopamine, 4‐HMA). Substrates that (i) robustly engage N232 and (ii) avoid competing H‐bonds that re‐anchor the substrate (e.g., to Y88) tend to exhibit low *K*_m_ and high *k*_cat_ (tyrosol, resveratrol, tyrosine). In contrast, β‐branching and fluorination promote halogen/H‐bond networks which are detrimental to catalysis, increasing *K*_*m*_ and/or reducing *k*_cat_, or abolishing activity (2‐FP).

## Discussion

This study provides new insights into the substrate specificity of *Ralstonia pseudosolanacearum* tyrosinase (RsTyr), a rare tyrosinase with an MDR favouring monophenols. By integrating *in silico* docking with kinetic characterisation, we identified structural determinants that govern RsTyr's broad substrate range and catalytic efficiency.

Docking analyses revealed that interactions with the activity controller residues (N228 and N232) and the flexible loop residue P239 are important for substrate orientation and catalysis. Interaction with N232 (the second activity controller residue) was by far the most common contact between substrate sidechain and the enzyme, reflective of past studies which identified interactions between the second activity controller and substrate sidechains [42]. The activity controller residues are also reported to have a role in facilitating catalysis via substrate deprotonation as described in other tyrosinases [13, 50, 51]. The presence of π–π interactions between substrate aromatic rings and copper‐coordinating histidines further supports their substrate anchoring function during catalysis, as observed in other tyrosinase enzymes previously [42]. The fact that the top pose of certain substrates into the AlphaFold model was not satisfactory and all top SWISS‐MODEL poses were could be due to the docking protocol being developed using PmTyr crystal structures, on which the SWISS‐MODEL structures were based.

Interestingly, RsTyr's preference for hydroxyl‐terminated side chains over amines and carboxylic acids suggests that electronic effects and steric factors jointly influence activity. Branched hydroxyl groups, as in octopamine and 4‐hydroxymandelic acid, correlated with reduced turnover, likely due to unfavourable positioning within the active site.

That RsTyr seemed to suffer substrate inhibition towards 4‐hydroxymandelic acid was surprising; it is not unheard of. Fungal tyrosinase from *Armillaria ostoyae* (AoTyr), when incubated with both tyrosine and L‐DOPA, displays substrate inhibition [53]. The inhibition observed has been linked to the activity controller residues of AoTyr (D262 & D266); however, a detailed mechanism for inhibition has not yet been elucidated.

Fluorinated phenols exhibited poor activity, consistent with their electron‐withdrawing nature and halogen bonding to catalytic histidines, which impeded correct orientation. While the preference of specific functional groups may be due to the interactions formed between the substrate and the enzyme, it could also be due to the tendency of the sidechain to donate or withdraw electrons from the aromatic ring. An estimation of the Hammett σ potential (a measure of the electron withdrawing or donating potential) of the substrates tested can be found in the Supporting Information (Table S6, [2]) and seems to have a loose correlation (*R* ^2^ = 0.487) with the observed turnover number (*k*_cat_) of the enzyme towards each substrate (Fig. S36, [2]).

There was broad agreement between docking predictions and kinetic data, validating the computational approach. However, it is of critical importance to take biological evidence into account when utilising docking software. While docking software such as GOLD can replicate or approximate the pose of substrates in an enzyme's active site (such as carried out here comparing the docking of tyrosine into PmTyr crystal structures to the structure 4p6r.pdb which contains tyrosine in the active site), the docking software does not innately know what the biologically active pose is. It searches for the most stable conformation of the ligand in the chosen site of the enzyme. This may result in poses which, while computationally more stable, are not biologically active, achieving a higher score [54]. Therefore, it is critical to assess docking results in the known biological context, as was carried out here.

Resveratrol and tyrosol displayed the highest catalytic efficiencies, surpassing tyrosine. These findings highlight RsTyr's potential for biotransformation of plant‐derived phenols, aligning with its ecological role as a plant pathogen [23].

A survey of kinetic properties of other tyrosinase enzymes shows RsTyr as the enzyme with the highest catalytic efficiency towards tyrosol, resveratrol and phenol and the second highest recorded catalytic efficiency towards tyrosine (Table S7, [22, 51, 55, 56, 57, 58, 59, 60, 61, 62, 63]). RsTyr's ability to efficiently hydroxylate monophenols expands its utility for producing catechols such as hydroxytyrosol and piceatannol, compounds with significant pharmaceutical and nutraceutical applications. Understanding the structural basis of substrate preference provides a foundation for rational enzyme engineering. Mutational studies targeting N228, N232 and P239 could further enhance monophenolase activity or broaden substrate scope.

The work presented herein aligned the results of kinetic analysis of RsTyr's activity towards a panel of substrates with *in silico* docking using RsTyr structures generated by two methods (SWISS‐MODEL & AlphaFold) to identify structural determinants of catalytic activity. Initial docking attempts using the RsTyr crystal structure (7xio.pdb) were unsuccessful, as were initial docking attempts using AlphaFold generated structures. We determined that the C‐terminal domain of RsTyr was retarding docking. As such, docking was carried out using models lacking the C‐terminal domain. C‐terminal domains are a common feature of eukaryotic tyrosinases wherein they act as a barrier to catalysis until removed [48]. In contrast, prokaryotic tyrosinases are sometimes found with C‐terminal domains as in RsTyr, sometimes without [42], and sometimes the tyrosinase catalytic domain is co‐expressed with an analogous ‘caddie’ protein which functions in a similar manner to the C‐terminal domain [64]. The C‐terminal domain of tyrosinase from *Verrucomicrobium spinosum* has been observed to change confirmation upon a shift in pH, allowing for substrate access [65]. In this study, RsTyr was expressed and purified as a 58 kDa protein, indicating the C‐terminal domain was intact. It is possible that the C‐terminal domain has an ability to change confirmation like the tyrosinase from *V. spinosum*, but this large‐scale re‐organisation was not captured in *in silico* model generation and docking studies.

While outside of the scope of this work, future studies could employ the use of Quantum mechanics/molecular mechanics (QM/MM) or molecular dynamics to further refine the structure of the tyrosinase and the poses of the docked compounds. Docking in this study captures enthalpic anchors (H‐bonds, halogen bonds) but incompletely samples induced fit and solvent dynamics. Consequently, we interpret model‐specific contacts as plausible poses rather than definitive states.

Crystallisation of RsTyr with bound substrates would clarify the role of the C‐terminal domain, which may influence substrate access or active site dynamics. Additionally, exploring the relationship between electronic properties (Hammett *σ* values) and catalytic performance could enable predictive models for substrate selection.

This work demonstrates that *Ralstonia pseudosolanacearum* tyrosinase (RsTyr) possesses a broad substrate range and exhibits exceptional monophenolase activity, particularly towards resveratrol and tyrosol. Combined *in silico* docking and kinetic analysis revealed that catalytic efficiency is strongly influenced by terminal functional groups and side‐chain length, with key roles played by residues N228, N232 and P239. These findings provide mechanistic insight into RsTyr's substrate specificity and highlight its potential for biocatalytic production of high value catechols. Future studies should focus on structural characterisation with bound substrates and targeted mutagenesis to further optimise enzyme performance for industrial applications.

## Conflicts of interest

James Britton and Fang Zhao are former employees of Bioplastech–Nova Mentis Ltd. Reeta Davis and Meg Walsh are current employees of Bioplastech–Nova Mentis. Kevin O'Connor is Chief Scientific Officer of Bioplastech–Nova Mentis. This work was carried out under the Irish Research Council Grant EBPPG/2021/54. The authors declare no conflict of interest.

## Author contributions

JB and FZ conceived the project. JB and FZ contributed to *in silico* experimental design and data analysis. JB, TN, RD and KOC contributed to *in vitro* experimental design and data analysis. JB, MW and RD acquired *in vitro* data. JB, RD and TN wrote the manuscript. TN, RD, DOC and KOC reviewed and edited the manuscript.

## Supporting information


**Fig. S1.** The active site of *P. megaterium* tyrosinase crystallised with tyrosol (4P6T.pdb).
**Fig. S2.** Top result of ensemble docking of tyrosol into *P. megaterium* tyrosinase crystal structures.
**Fig. S3.** Alignment of 4P6R.pdb with PmTyr ensemble docking of tyrosine with a top down (top) and profile (bottom) view.
**Fig. S4.** Alignment of 4P6T.pdb with PmTyr ensemble docking of tyrosol with a top down (top) and profile (bottom) view.
**Fig. S5.** (A–E) Peak absorption and molar extinction coefficient calculations for substituted quinone compounds formed by tyrosinase action on resveratrol and 4‐hydroxymandelic acid.
**Fig. S6.** A screenshot of the sequence analysis of *R. pseudosolanacearum* tyrosinase by InterPro [1] and the representative key.
**Fig. S7.** Tyrosine docked into 7XIO crystal structure and zoomed view on the active site.
**Fig. S8.** Tyrosol docked into 7XIO crystal structure and zoomed view on the active site.
**Fig. S9.** Tyrosine docked into RsTyr AlphaFold 3 predicted model structure and zoomed view on the active site.
**Fig. S10.** Tyrosol docked into RsTyr AlphaFold 3 predicted model structure and zoomed view on the active site.
**Fig. S11.** Top scoring SWISS‐MODEL RsTyr ensemble docking solution for tyrosine.
**Fig. S12.** Top scoring SWISS‐MODEL RsTyr ensemble docking solution for tyrosol.
**Fig. S13.** Top scoring SWISS‐MODEL RsTyr ensemble docking solution for tyramine.
**Fig. S14.** Top scoring SWISS‐MODEL RsTyr ensemble docking solution for octopamine.
**Fig. S15.** Top scoring SWISS‐MODEL RsTyr ensemble docking solution for resveratrol.
**Fig. S16.** Top scoring SWISS‐MODEL RsTyr ensemble docking solution for phenol.
**Fig. S17.** Top scoring SWISS‐MODEL RsTyr ensemble docking solution for 2‐flurophenol.
**Fig. S18.** Top scoring SWISS‐MODEL RsTyr ensemble docking solution for 3‐fluorophenol.
**Fig. S19.** Top scoring SWISS‐MODEL RsTyr ensemble docking solution for 4‐hydroxybenzoic acid.
**Fig. S20.** Top scoring SWISS‐MODEL RsTyr ensemble docking solution for 4‐hydroxymandelic acid.
**Fig. S21.** Top scoring SWISS‐MODEL RsTyr ensemble docking solution for 4‐hydroxyphenylacetic acid.
**Fig. S22.** Top scoring SWISS‐MODEL RsTyr ensemble docking solution for 4‐methoxyphenylacetic acid.
**Fig. S23.** Top scoring AlphaFold RsTyr model docking solution for Tyrosine.
**Fig. S24.** Top scoring AlphaFold RsTyr model docking solution for Tyrosol.
**Fig. S25.** Top scoring AlphaFold RsTyr model docking solution for Tyramine.
**Fig. S26.** Top scoring AlphaFold RsTyr model docking solution for Octopamine.
**Fig. S27.** Top scoring AlphaFold RsTyr model docking solution for Resveratrol.
**Fig. S28.** Top scoring AlphaFold RsTyr model docking solution for Phenol.
**Fig. S29.** Top scoring AlphaFold RsTyr model docking solution for 2‐fluorophenol.
**Fig. S30.** Top scoring AlphaFold RsTyr model docking solution for 3‐fluorophenol.
**Fig. S31.** Top scoring AlphaFold RsTyr model docking solution for 4‐hydroxybenzoic acid.
**Fig. S32.** Top scoring AlphaFold RsTyr model docking solution for 4‐hydroxymandelic acid.
**Fig. S33.** Top scoring AlphaFold RsTyr model docking solution for 4‐hydroxyphenylacetic acid.
**Fig. S34.** Top scoring AlphaFold RsTyr model docking solution for 4‐methoxyphenylacetic acid.
**Fig. S35.** 4‐hydroxymandelic acid Δ317 absorption change in a 1‐h kinetic assay with RsTyr at 317 nm.
**Fig. S36.** Predicted Hammetts *σ* values versus observed *k*_*cat*_(min^−1^) for all non‐fluorinated substrates tested. Hammetts σ value predictions were made using the Ertl‐molecular Hammett Sigma Constant calculator [2].
**Table S1.** *Priestia* megaterium tyrosinase PDB files used for building homology models of the *Ralstonia pseudosolanacearum* tyrosinase.
**Table S2.** A list of *R. pseudosolanacearum* tyrosinase models used in this study.
**Table S3.** A list of *P. megaterium* tyrosinase GOLD docking PDB files.
**Table S4.** A list of *R. pseudosolanacearum* tyrosinase GOLD docking PDB files.
**Table S5.** An overview of *in silico* GOLD docking scores (ChemPLP fitness) of a range of substrates into RsTyr models.
**Table S6.** Hammet σ constant prediction for all non‐fluorinated substrates tested in this study.
**Table S7.** A comparison of *K*_m_, *k*_cat_ and *k*_cat_/*K*_m_ of various tyrosinase enzymes towards tyrosine, tyrosol, resveratrol, tyramine, phenol and 4‐hydroxybenzoic acid.

## Data Availability

The data that supports the findings of this study are available in the Supporting Information of this article. The structural data that support these findings are openly available on the Zenodo repository at https://doi.org/10.5281/zenodo.21128270.
