# Supplementary material for: In silico and in vitro exploration of a tyrosinase for biocatalytic production of catechols
Source: FEBS Open Bio. 2026 Jul 28:10.1002/2211-5463.70311. Online ahead of print. doi: 10.1002/2211-5463.70311 (PMC13416272; doi:10.1002/2211-5463.70311)
Supplement: Supplementary file 1 — Fig. S1. The active site of P. megaterium tyrosinase crystallised with tyrosol (4P6T.pdb). Fig. S2. Top result of ensemble docking of tyrosol into P. megaterium tyrosinase crystal structures. Fig. S3. Alignment of 4P6R.pdb with PmTyr ensemble docking of tyrosine with a top down (top) and profile (bottom) view. Fig. S4. Alignment of 4P6T.pdb with PmTyr ensemble docking of tyrosol with a top down (top) and profile (bottom) view. Fig. S5. (A–E) Peak absorption and molar extinction coefficient calculations for substituted quinone compounds formed by tyrosinase action on resveratrol and 4‐hydroxymandelic acid. Fig. S6. A screenshot of the sequence analysis of R. pseudosolanacearum tyrosinase by InterPro [1] and the representative key. Fig. S7. Tyrosine docked into 7XIO crystal structure and zoomed view on the active site. Fig. S8. Tyrosol docked into 7XIO crystal structure and zoomed view on the active site. Fig. S9. Tyrosine docked into RsTyr AlphaFold 3 predicted model structure and zoomed view on the active site. Fig. S10. Tyrosol docked into RsTyr AlphaFold 3 predicted model structure and zoomed view on the active site. Fig. S11. Top scoring SWISS‐MODEL RsTyr ensemble docking solution for tyrosine. Fig. S12. Top scoring SWISS‐MODEL RsTyr ensemble docking solution for tyrosol. Fig. S13. Top scoring SWISS‐MODEL RsTyr ensemble docking solution for tyramine. Fig. S14. Top scoring SWISS‐MODEL RsTyr ensemble docking solution for octopamine. Fig. S15. Top scoring SWISS‐MODEL RsTyr ensemble docking solution for resveratrol. Fig. S16. Top scoring SWISS‐MODEL RsTyr ensemble docking solution for phenol. Fig. S17. Top scoring SWISS‐MODEL RsTyr ensemble docking solution for 2‐flurophenol. Fig. S18. Top scoring SWISS‐MODEL RsTyr ensemble docking solution for 3‐fluorophenol. Fig. S19. Top scoring SWISS‐MODEL RsTyr ensemble docking solution for 4‐hydroxybenzoic acid. Fig. S20. Top scoring SWISS‐MODEL RsTyr ensemble docking solution for 4‐hydroxymandelic acid. Fig. S21. Top scoring S [file FEB4-9999-0-s001.pdf]

Biocatalytic production of catechols: *In silico* and *in vitro* exploration of a tyrosinase

Supplementary Figures

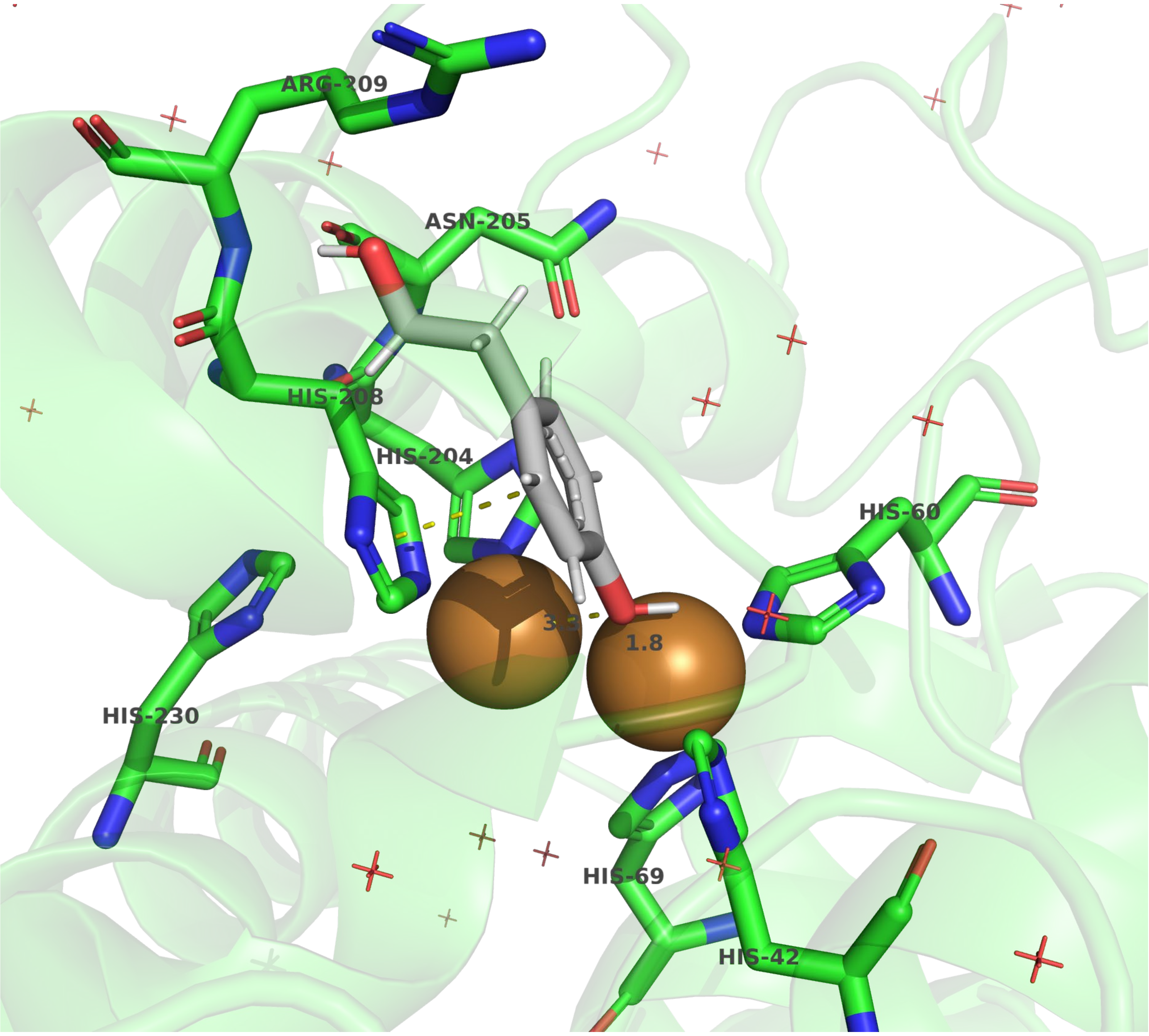

Figure S1. The active site of *P. megaterium* tyrosinase crystallised with tyrosol (4P6T.pdb, [41]). Tyrosol is depicted as a grey stick model. The 6 Cu coordinating histidines in the tyrosinase active site and the two activity controller residues (N228 and N232) are depicted as stick models. Cu ions are brown/copper colour spheres. The protein backbone is depicted as a green ribbon. Water ions are red stars. Dashed lines represent interactions between the substrate and the enzyme, with numbers representing the distance in angstrom (Å).

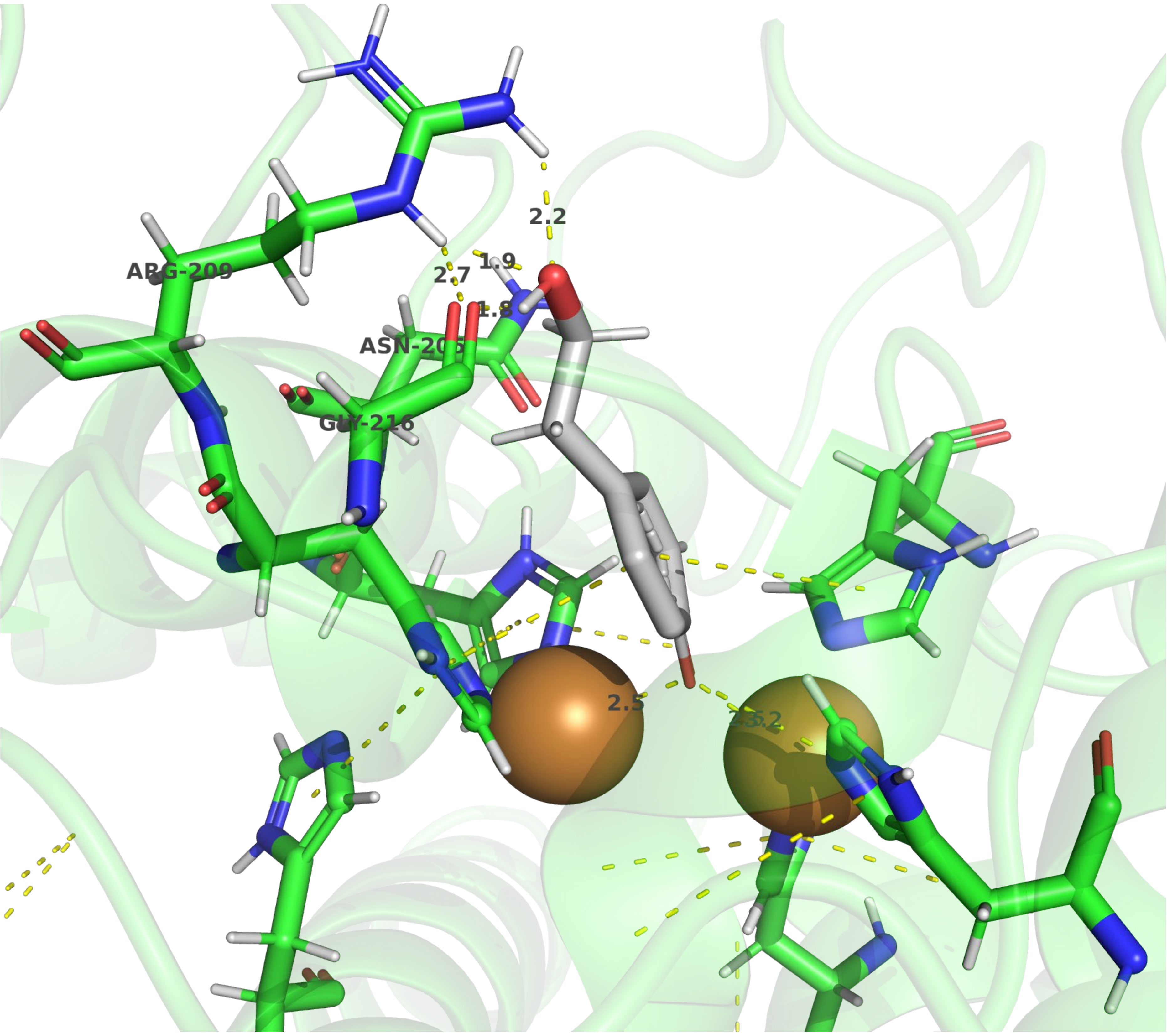

Figure S2. Top result of ensemble docking of tyrosol into *P. megaterium* tyrosinase crystal structures. Tyrosine is depicted as a grey stick model. The 6 Cu coordinating histidines in the tyrosinase active site and the two activity controller residues (N228 and N232) are depicted as stick models. Cu ions are brown/copper colour spheres. The protein backbone is depicted as a green ribbon. Water ions are red stars. Dashed lines represent interactions between the substrate and the enzyme, with numbers representing the distance in angstrom (Å).

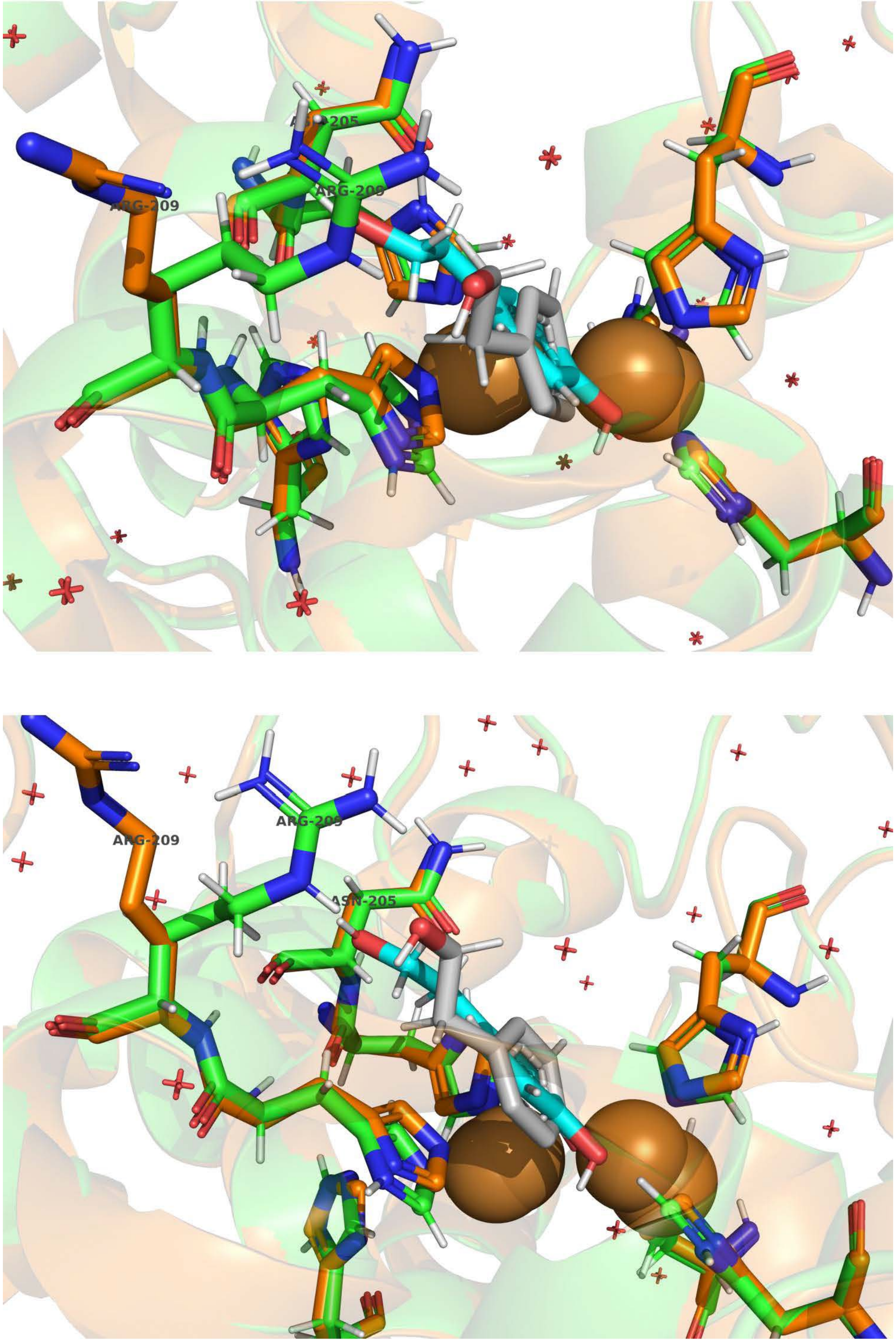

Figure S3. Alignment of 4P6R.pdb [41] with PmTyr ensemble docking of tyrosine with a top down (top) and profile (bottom) view. Tyrosine is depicted as either a grey (4P6R.pdb) or teal (docking) stick model. The 6 Cu coordinating histidines in the tyrosinase active site and the two activity controller residues (N228 and N232) are depicted as stick models. Cu ions are brown/copper colour spheres. The protein backbone is depicted in green (4P6R.pdb) or orange (docking). Water ions are red stars. Dashed lines represent interactions between the substrate and the enzyme, with numbers representing the distance in angstrom (Å).

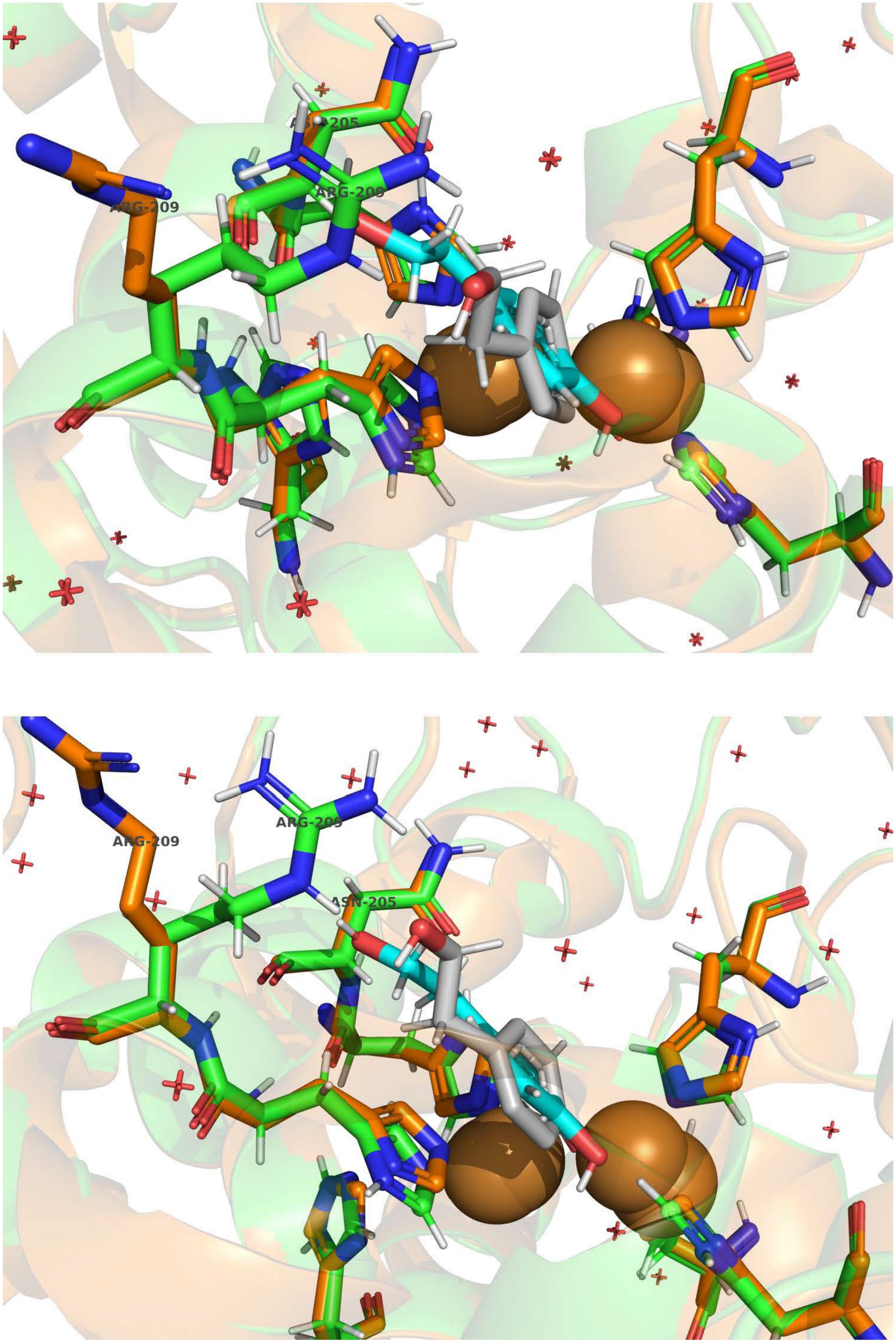

Figure S4. Alignment of 4P6T.pdb [41] with PmTyr ensemble docking of tyrosol with a top down (top) and profile (bottom) view. Tyrosol is depicted as either a grey (4P6R.pdb) or teal (docking) stick model. The 6 Cu coordinating histidines in the tyrosinase active site and the two activity controller residues (N228 and N232) are depicted as stick models. Cu ions are brown/copper colour spheres. The protein backbone is depicted in green (4P6R.pdb) or orange (docking). Water ions are red stars. Dashed lines represent interactions between the substrate and the enzyme, with numbers representing the distance in angstrom (Å).

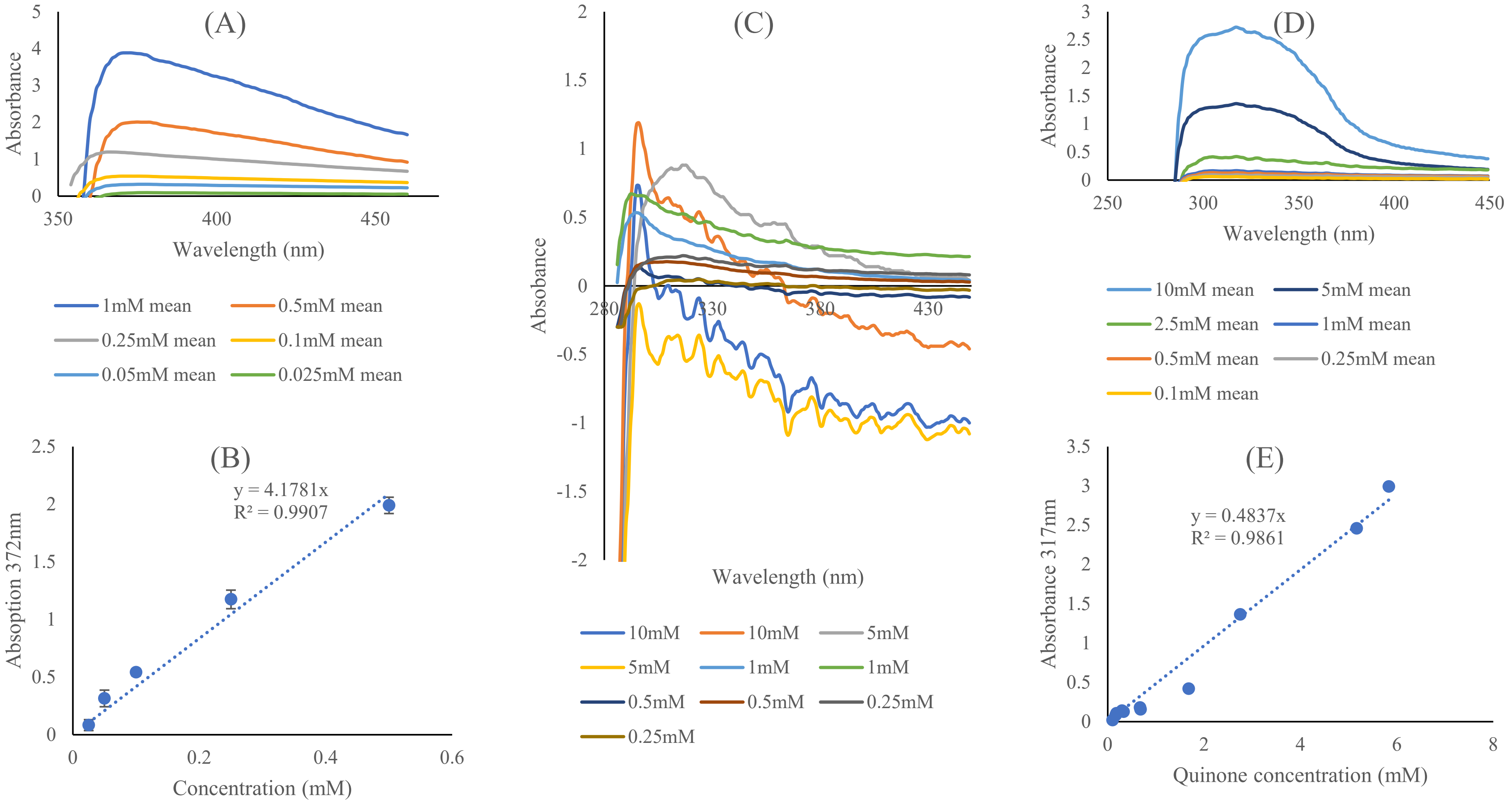

Figure S5A-E. Peak absorption and molar extinction coefficient calculations for substituted quinone compounds formed by tyrosinase action on resveratrol and 4-hydroxymandelic acid. (**A)** 4-substituted resveratrol-quinone spectra. **(B)** 4-substituted resveratrol-quinone 372 nm standard curve **(C)** 4-substituted 4-hydroxybenzoic acid-quinone spectra **(D)** 4-substituted 4-hydroxymandelic acid-quinone spectra **(E)** 4-substituted 4-hydroxymandelic acid-quinone standard curve. Error bars indicate one standard deviation from the mean.

A brief explanation of the process:

HPLC standards for the substrates are created. Following this a range of concentrations of each substrate are incubated with active tyrosinase overnight. The following morning the absorption spectrum of the samples are measured noting the absorption maximum wavelength. The level of substrate depletion is measured also, based on HPLC analysis. The formation of the pigmented quinone at the absorption maximum is proportional to the amount of substrate depleted.

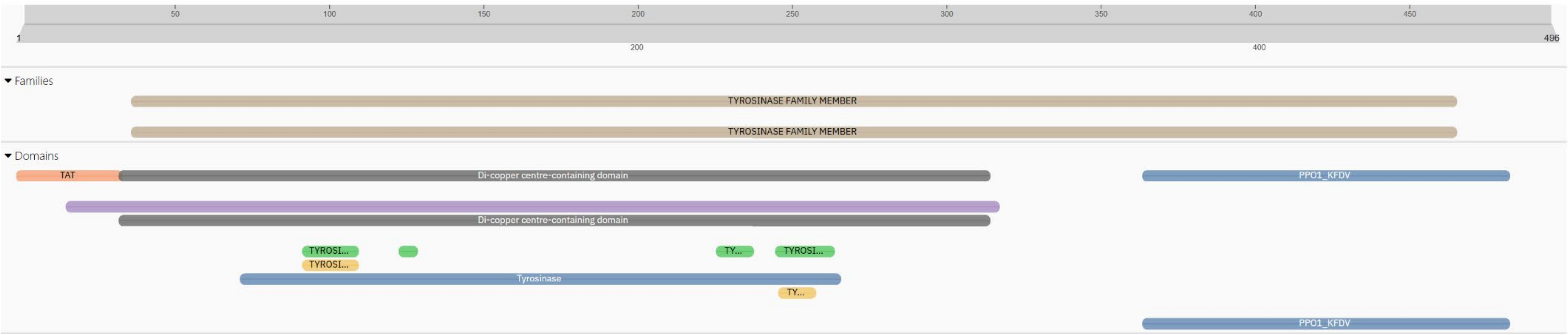

Representative families

F Tyrosinase/Hemocyanin - Tyrosinase and Hemocyanin
PANTHER: TYROSINASE FAMILY MEMBER - TYROSINASE FAMILY MEMBER

Representative domains

H Di-copper_centre_dom_sf - Di-copper centre-containing domain superfamily
CATHGENE3D: G3DSA:1.10.1280.10
SSF: Di-copper centre-containing domain - Di-copper centre-containing domain

D Tyrosinase_Cu-bd - Tyrosinase copper-binding domain
PRINTS: TYROSINASE - Tyrosinase copper-binding domain signature
PROSITE: TYROSINASE_1 - Tyrosinase CuA-binding region signature.
PFAM: Tyrosinase - Common central domain of tyrosinase
PROSITE: TYROSINASE_2 - Tyrosinase and hemocyanins CuB-binding region signature.

D Polyphenol_oxidase_C - Polyphenol oxidase, C-terminal
PFAM: PPO1_KFDV - Chloroplastic Polyphenol Oxidase C-terminal domain

Figure S6. Screenshot of sequence analysis of *Ralstonia pseudosolanacearum* tyrosinase by InterPro and the representative family key.

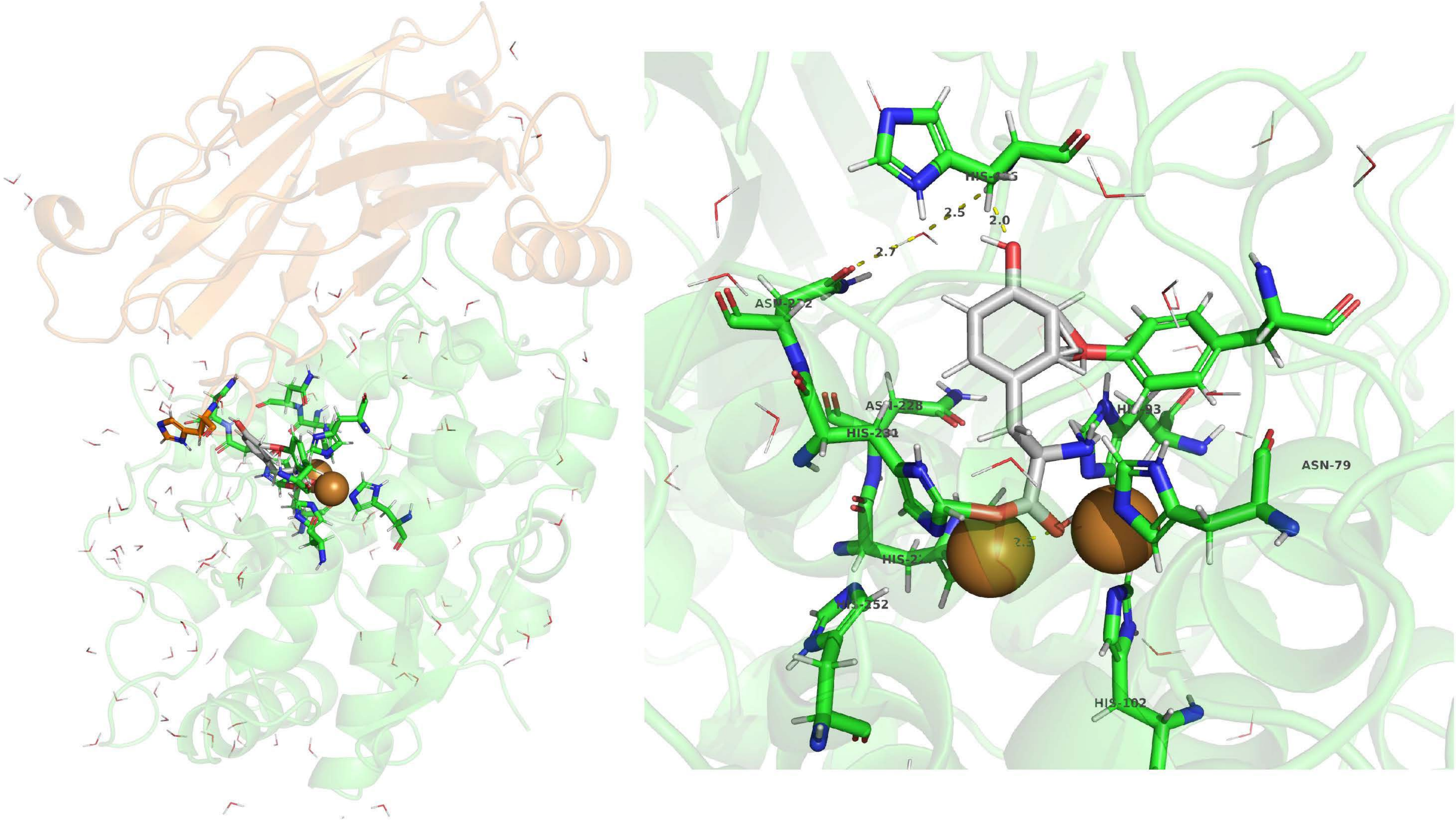

Figure S7. Tyrosine docked into 7XIO crystal structure and close view on the active site. Green ribbons depict the catalytic domain of the enzyme, orange ribbons depict the C-terminal domain and red ribbons depict the N-terminal signal peptide. Both tyrosine and tyrosol are depicted as grey stick models. In all models the 6 Cu coordinating histidines in the tyrosinase active site, the two activity controller residues (N228 and N232) and other residues which the substrate interacts with are depicted as stick models. Cu ions are brown/copper colour spheres.

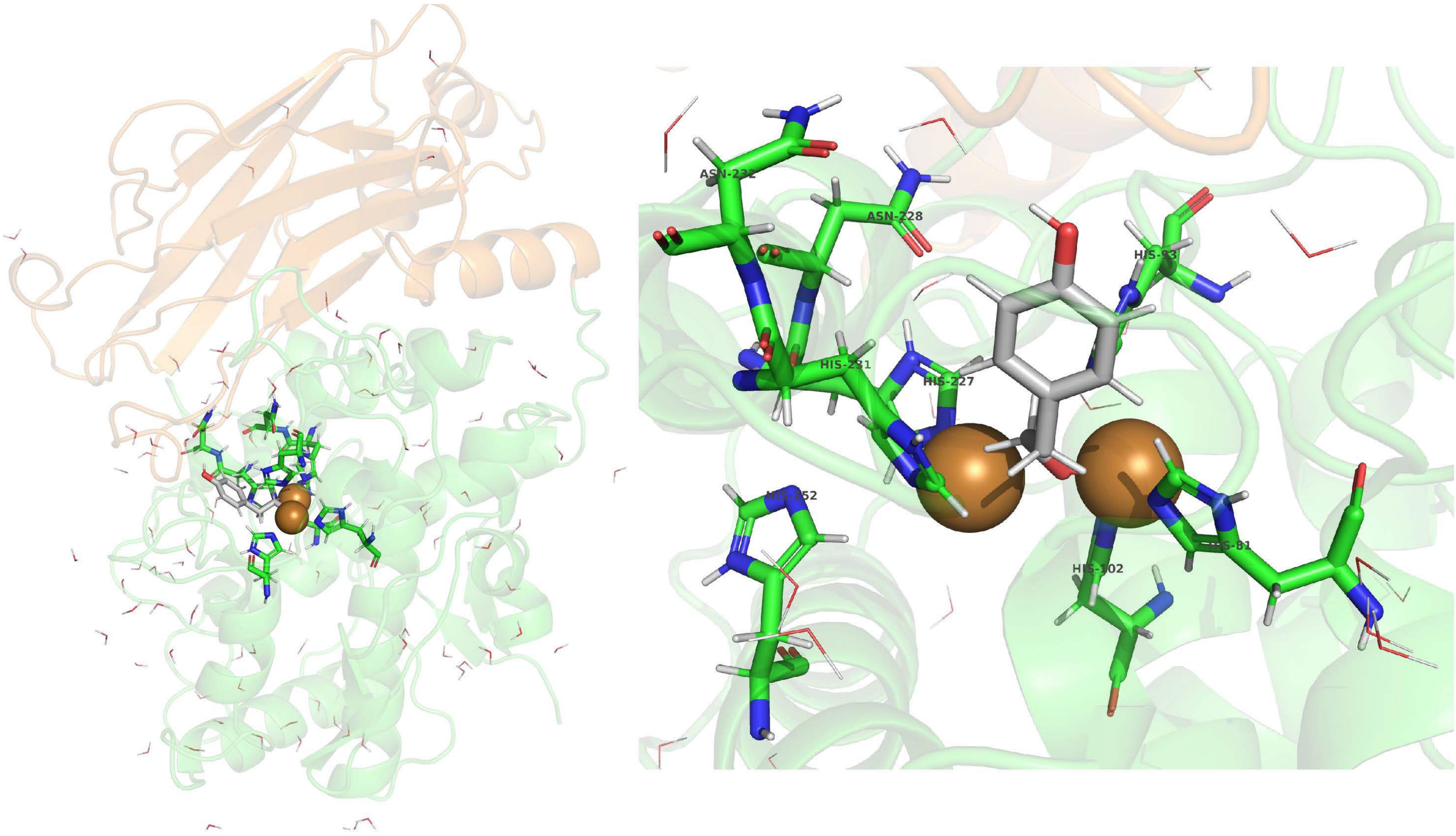

Figure S8. Tyrosol docked into 7XIO crystal structure and close view on the active site. Green ribbons depict the catalytic domain of the enzyme, orange ribbons depict the C-terminal domain and red ribbons depict the N-terminal signal peptide. Both tyrosine and tyrosol are depicted as grey stick models. In all models the 6 Cu coordinating histidines in the tyrosinase active site, the two activity controller residues (N228 and N232) and other residues which the substrate interacts with are depicted as stick models. Cu ions are brown/copper colour spheres.

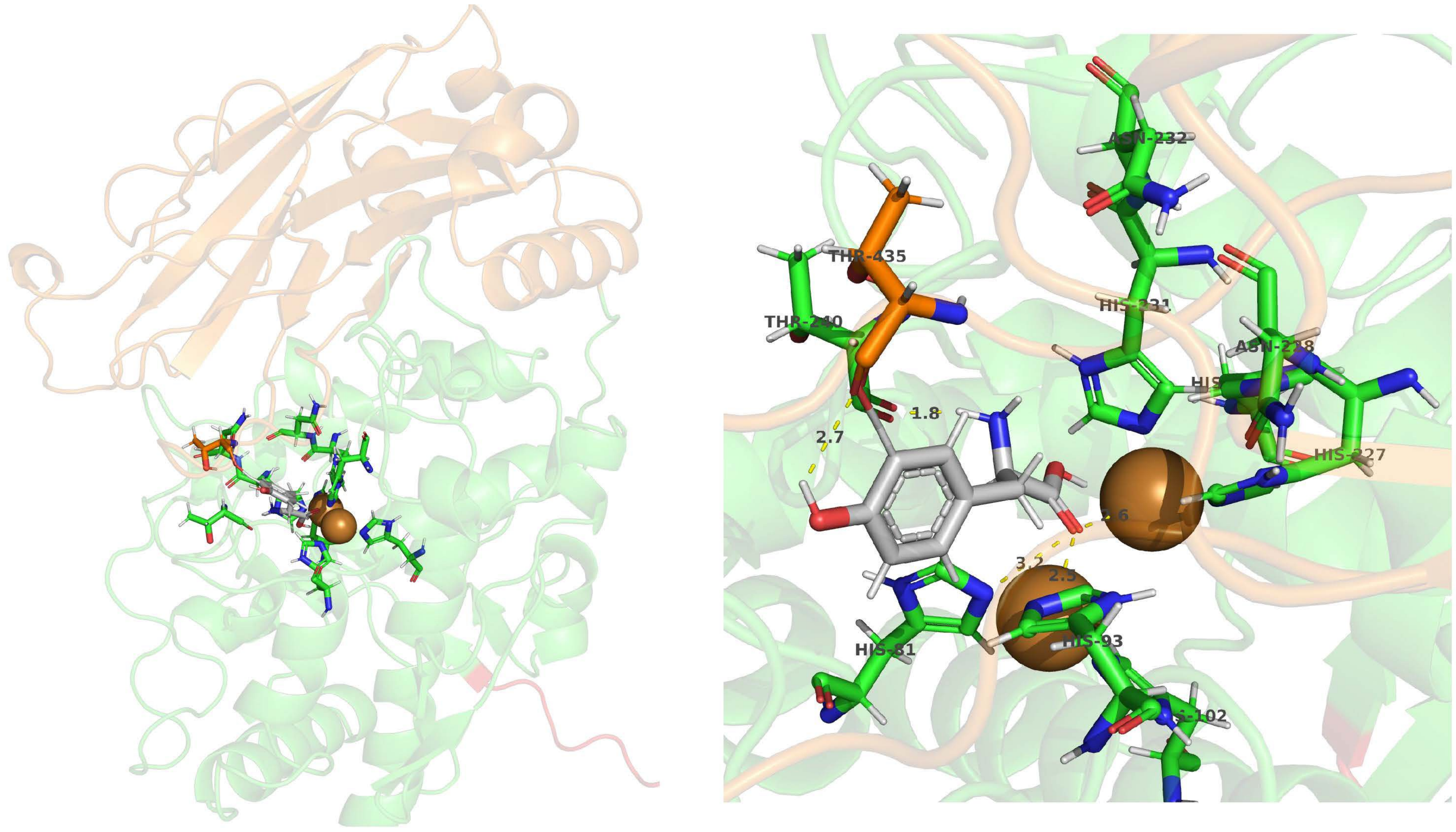

Figure S9. Tyrosine docked into RsTyr AlphaFold 3 predicted model structure and close view on the active site. Green ribbons depict the catalytic domain of the enzyme, orange ribbons depict the C-terminal domain and red ribbons depict the N-terminal signal peptide. Both tyrosine and tyrosol are depicted as grey stick models. In all models the 6 Cu coordinating histidines in the tyrosinase active site, the two activity controller residues (N228 and N232) and other residues which the substrate interacts with are depicted as stick models. Cu ions are brown/copper colour spheres.

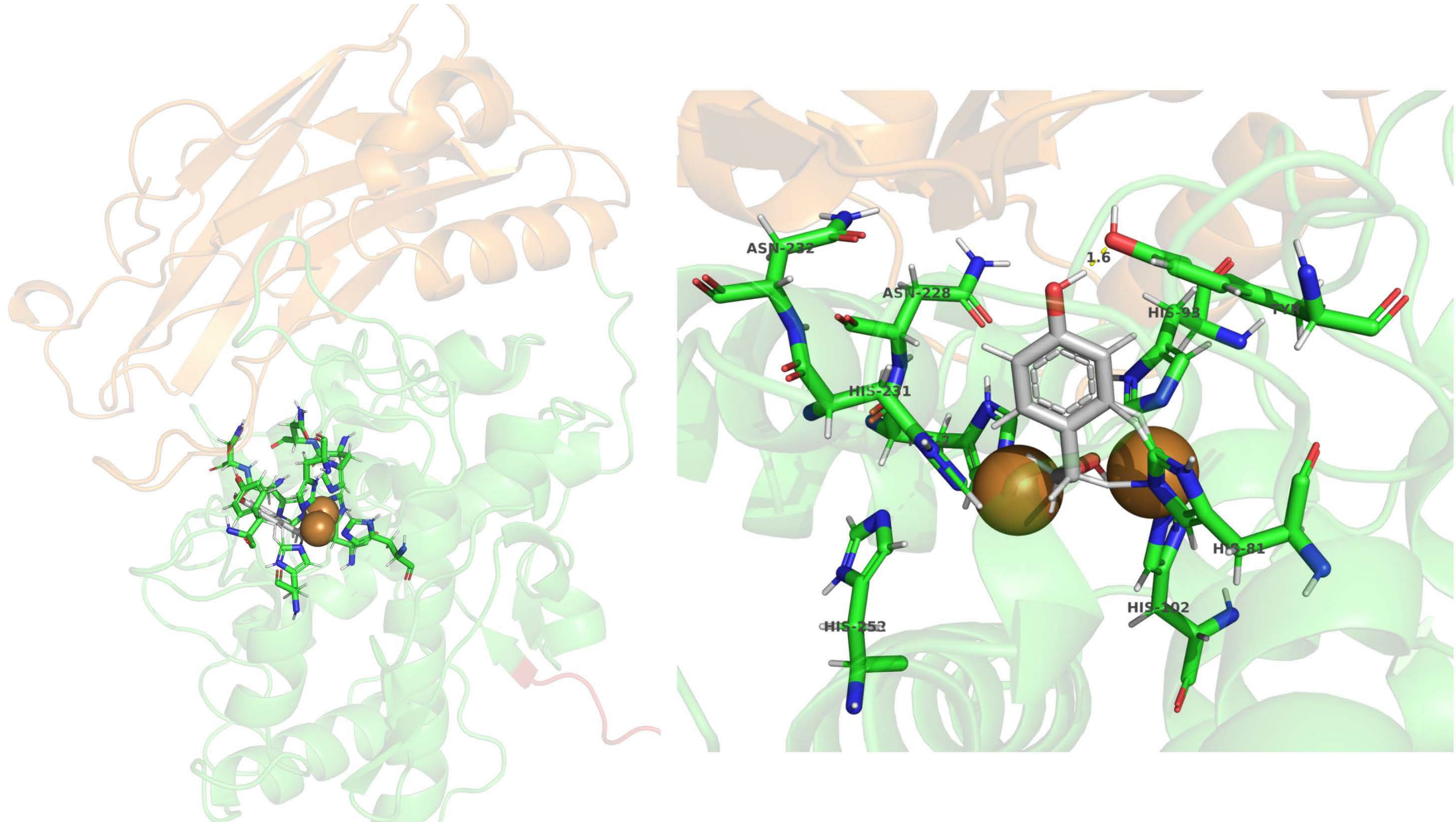

Figure S10. Tyrosol docked into RsTyr AlphaFold 3 predicted model structure and close view on the active site. Green ribbons depict the catalytic domain of the enzyme, orange ribbons depict the C-terminal domain and red ribbons depict the N-terminal signal peptide. Both tyrosine and tyrosol are depicted as grey stick models. In all models the 6 Cu coordinating histidines in the tyrosinase active site, the two activity controller residues (N228 and N232) and other residues which the substrate interacts with are depicted as stick models. Cu ions are brown/copper colour spheres.

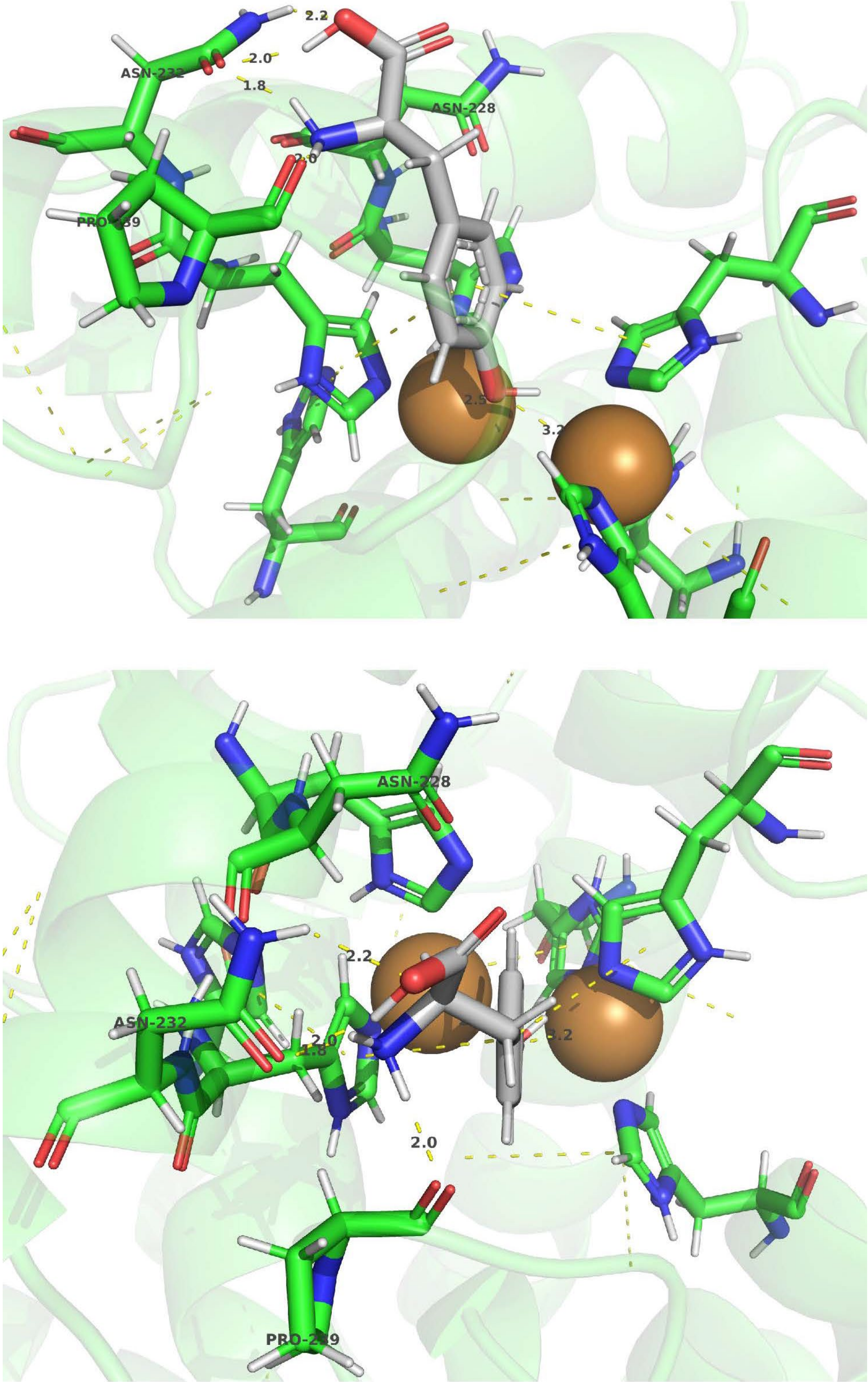

Figure S11. Top scoring SWISS-MODEL RsTyr ensemble docking solution for tyrosine. A profile (top) and top-down (bottom) view is provided. In both images green ribbons depict the catalytic domain of the enzyme. Tyrosine is depicted as grey stick models. In both models the 6 Cu coordinating histidines in the tyrosinase active site, the two activity controller residues (N228 and N232) and other residues which the substrate interacts with are depicted as stick models. Cu ions are brown/copper colour spheres. Yellow dotted lines depict interactions between residues, substrates and cofactors. Numbers on the yellow lines indicate the distance of the bond in angstroms (Å).

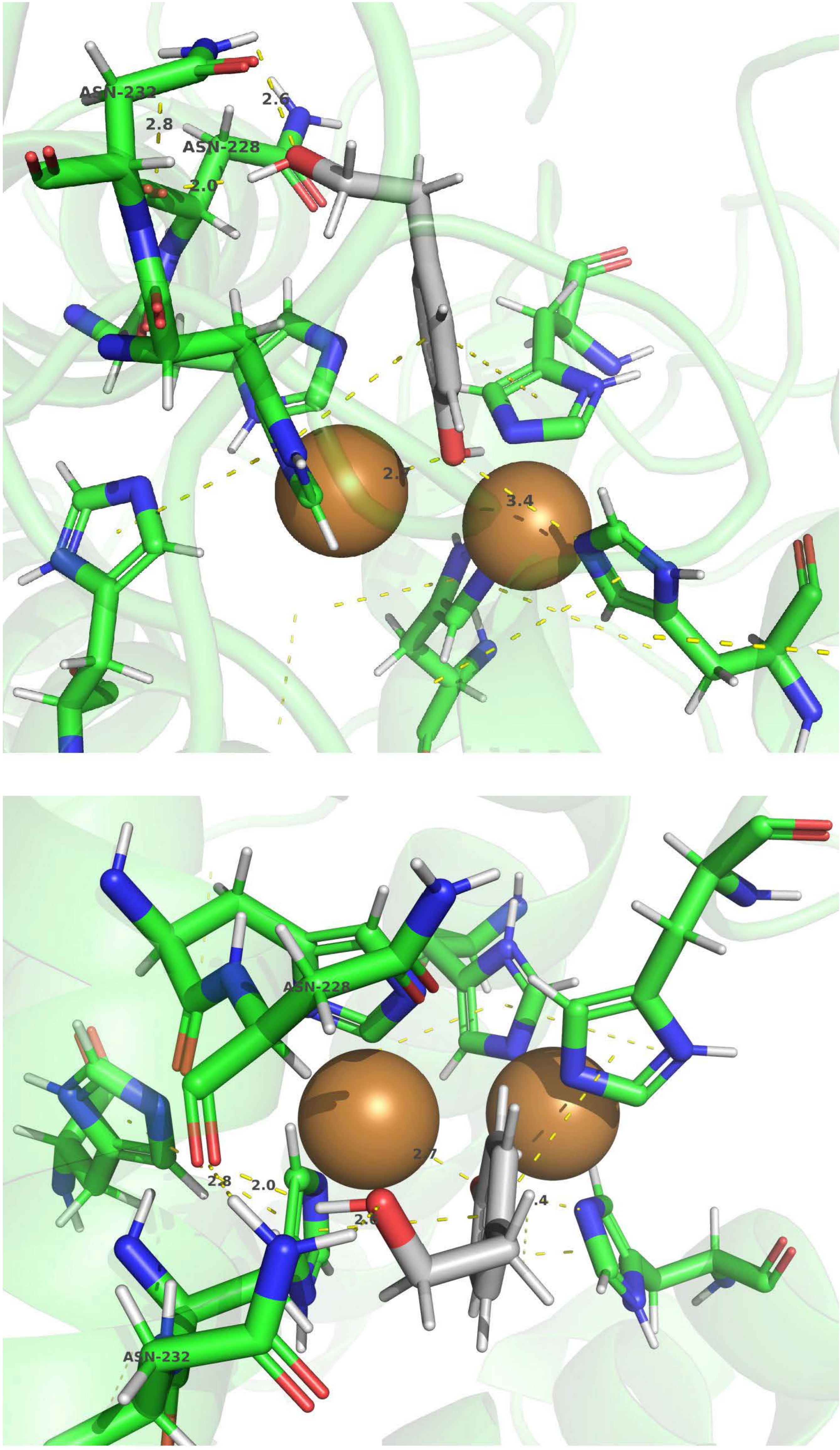

Figure S12. Top scoring SWISS-MODEL RsTyr ensemble docking solution for tyrosol. A profile (top) and top-down (bottom) view is provided. In both images green ribbons depict the catalytic domain of the enzyme. Tyrosol is depicted as grey stick models. In both models the 6 Cu coordinating histidines in the tyrosinase active site, the two activity controller residues (N228 and N232) and other residues which the substrate interacts with are depicted as stick models. Cu ions are brown/copper colour spheres. Yellow dotted lines depict interactions between residues, substrates and cofactors. Numbers on the yellow lines indicate the distance of the bond in angstroms (Å).

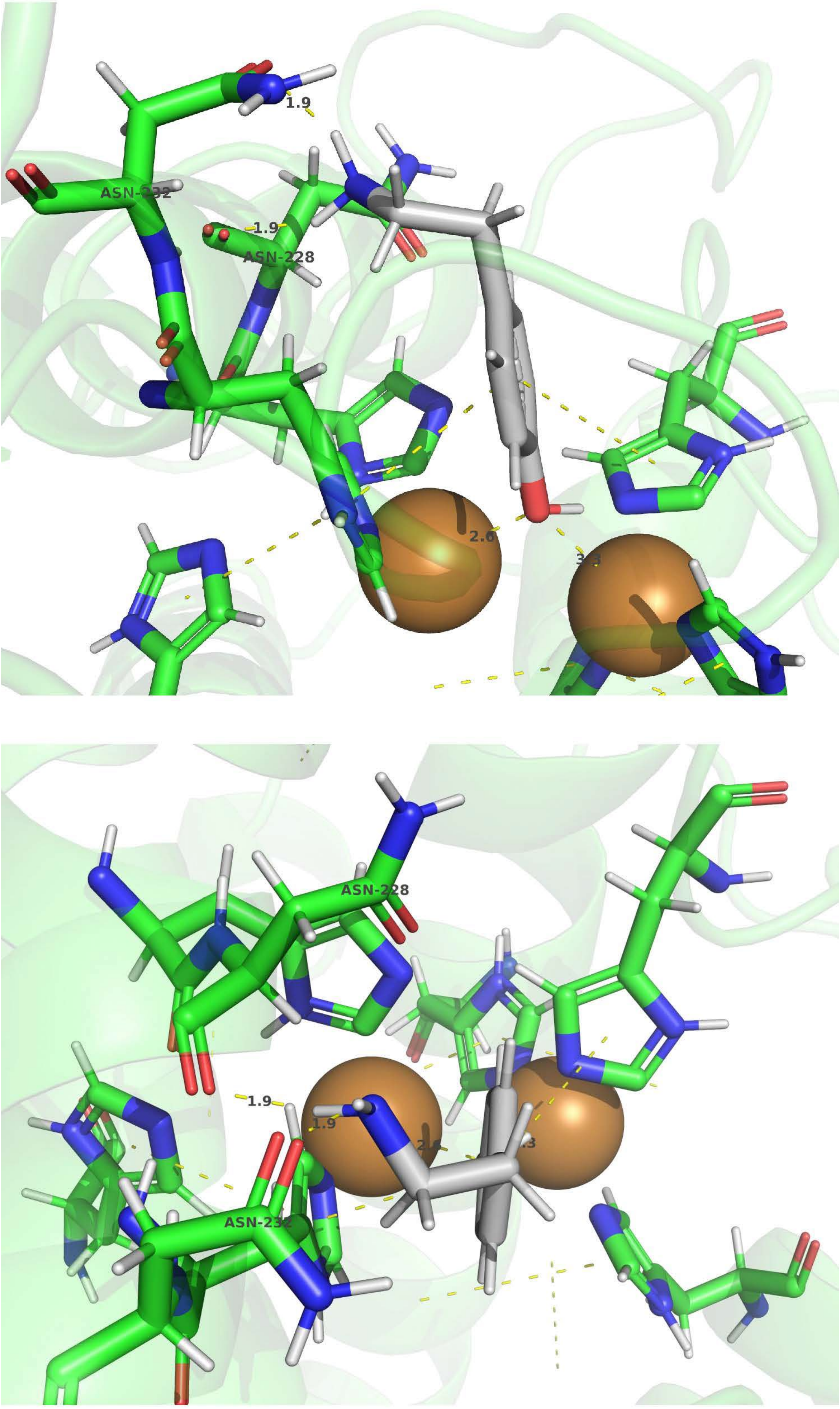

Figure S13. Top scoring SWISS-MODEL RsTyr ensemble docking solution for tyramine. A profile (top) and top-down (bottom) view is provided. In both images green ribbons depict the catalytic domain of the enzyme. Tyramine is depicted as grey stick models. In both models the 6 Cu coordinating histidines in the tyrosinase active site, the two activity controller residues (N228 and N232) and other residues which the substrate interacts with are depicted as stick models. Cu ions are brown/copper colour spheres. Yellow dotted lines depict interactions between residues, substrates and cofactors. Numbers on the yellow lines indicate the distance of the bond in angstroms (Å).

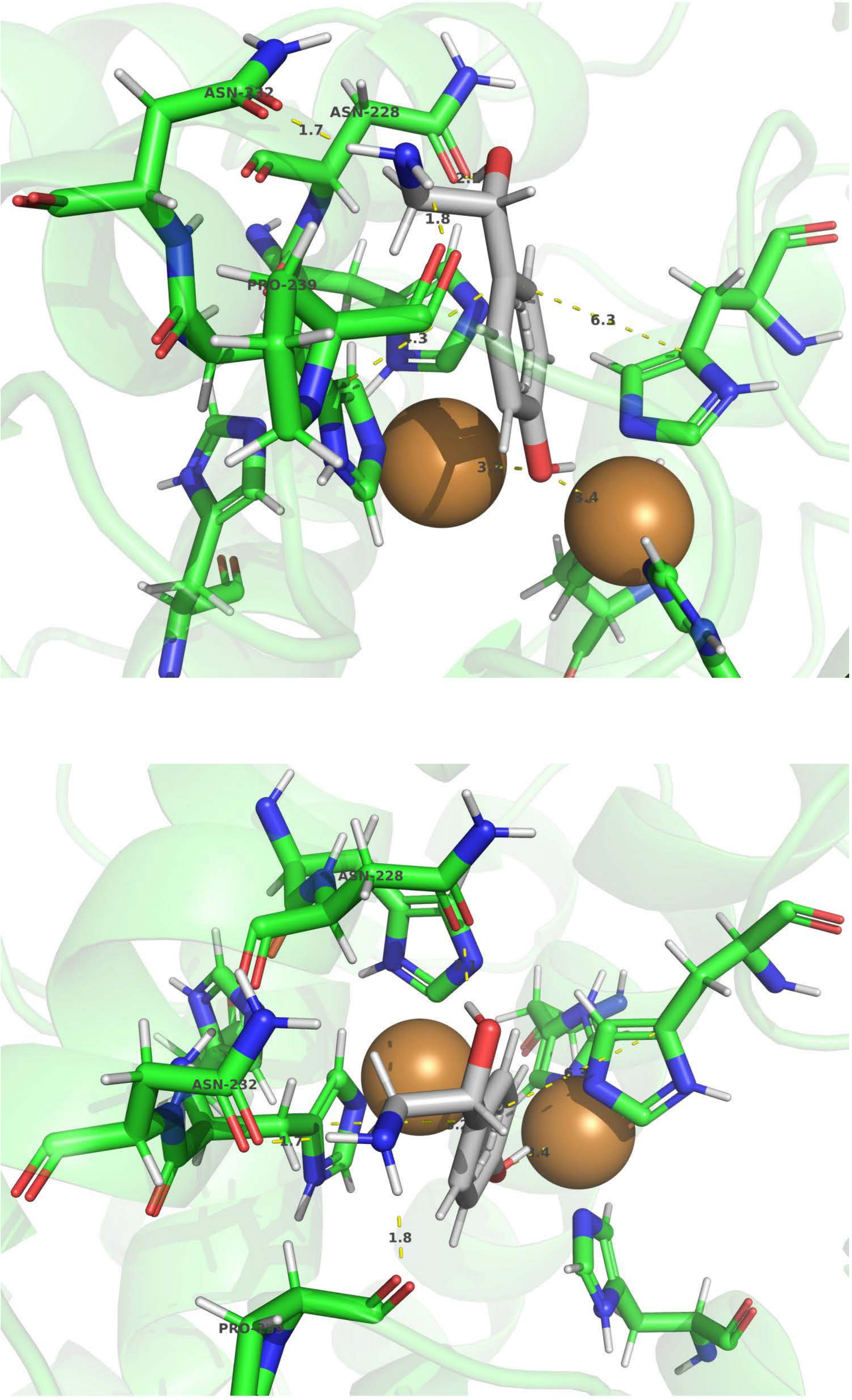

Figure S14. Top scoring SWISS-MODEL RsTyr ensemble docking solution for octopamine. A profile (top) and top-down (bottom) view is provided. In both images green ribbons depict the catalytic domain of the enzyme. Octopamine is depicted as grey stick models. In both models the 6 Cu coordinating histidines in the tyrosinase active site, the two activity controller residues (N228 and N232) and other residues which the substrate interacts with are depicted as stick models. Cu ions are brown/copper colour spheres. Yellow dotted lines depict interactions between residues, substrates and cofactors. Numbers on the yellow lines indicate the distance of the bond in angstroms (Å).

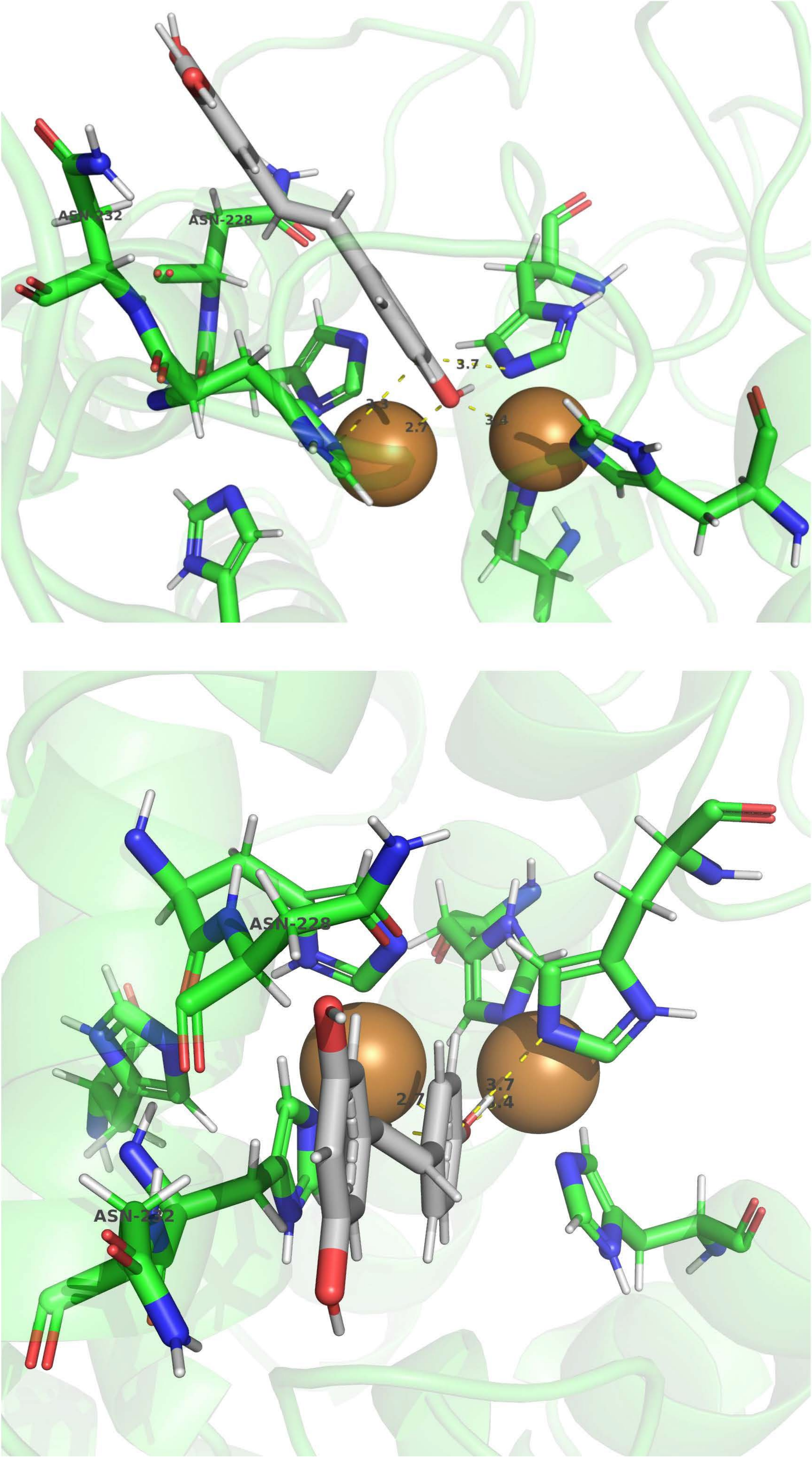

Figure S15. Top scoring SWISS-MODEL RsTyr ensemble docking solution for resveratrol. A profile (top) and top-down (bottom) view is provided. In both images green ribbons depict the catalytic domain of the enzyme. Resveratrol is depicted as grey stick models. In both models the 6 Cu coordinating histidines in the tyrosinase active site, the two activity controller residues (N228 and N232) and other residues which the substrate interacts with are depicted as stick models. Cu ions are brown/copper colour spheres. Yellow dotted lines depict interactions between residues, substrates and cofactors. Numbers on the yellow lines indicate the distance of the bond in angstroms (Å).

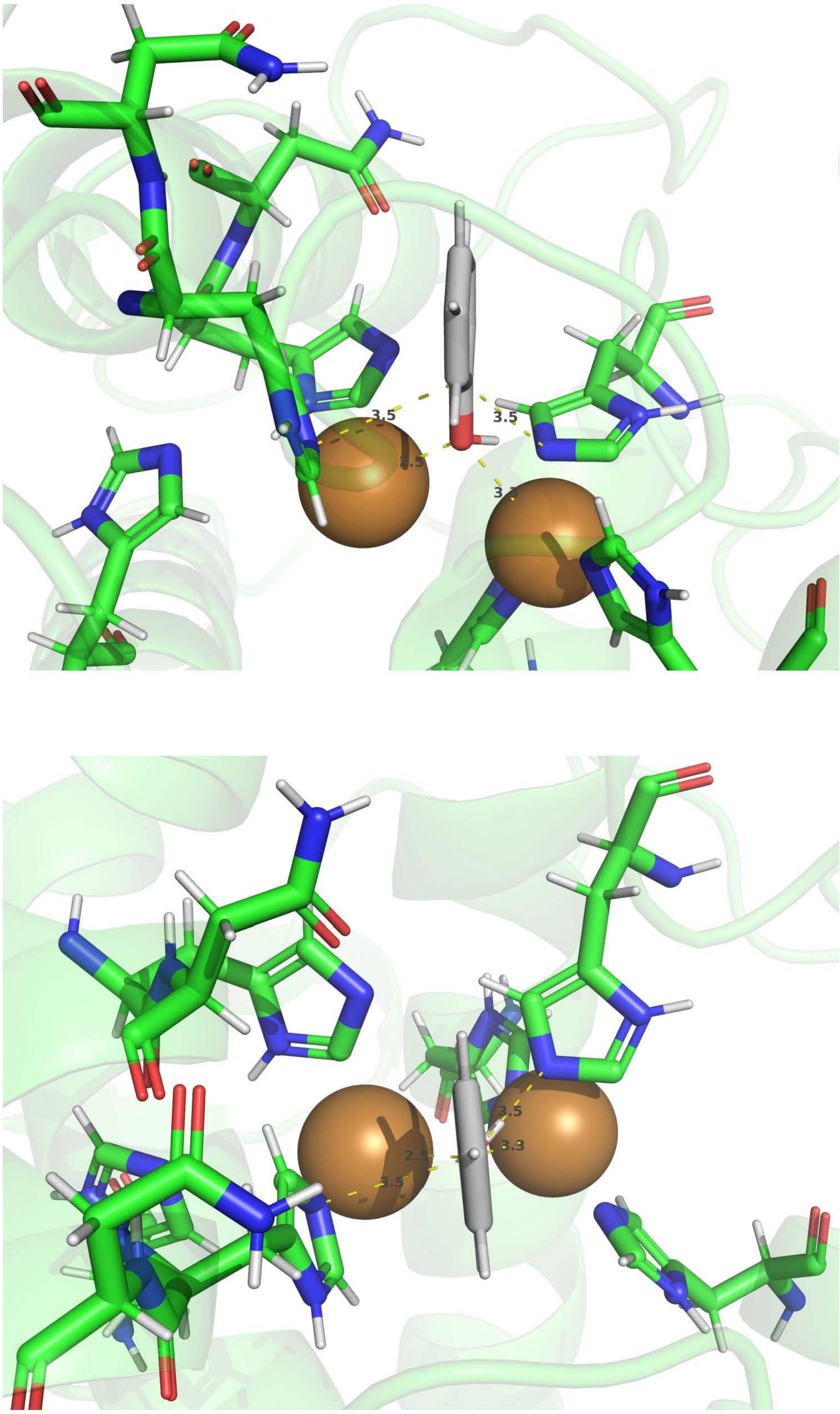

Figure S16. Top scoring SWISS-MODEL RsTyr ensemble docking solution for phenol. A profile (top) and top-down (bottom) view is provided. In both images green ribbons depict the catalytic domain of the enzyme. Phenol is depicted as grey stick models. In both models the 6 Cu coordinating histidines in the tyrosinase active site, the two activity controller residues (N228 and N232) and other residues which the substrate interacts with are depicted as stick models. Cu ions are brown/copper colour spheres. Yellow dotted lines depict interactions between residues, substrates and cofactors. Numbers on the yellow lines indicate the distance of the bond in angstroms (Å).

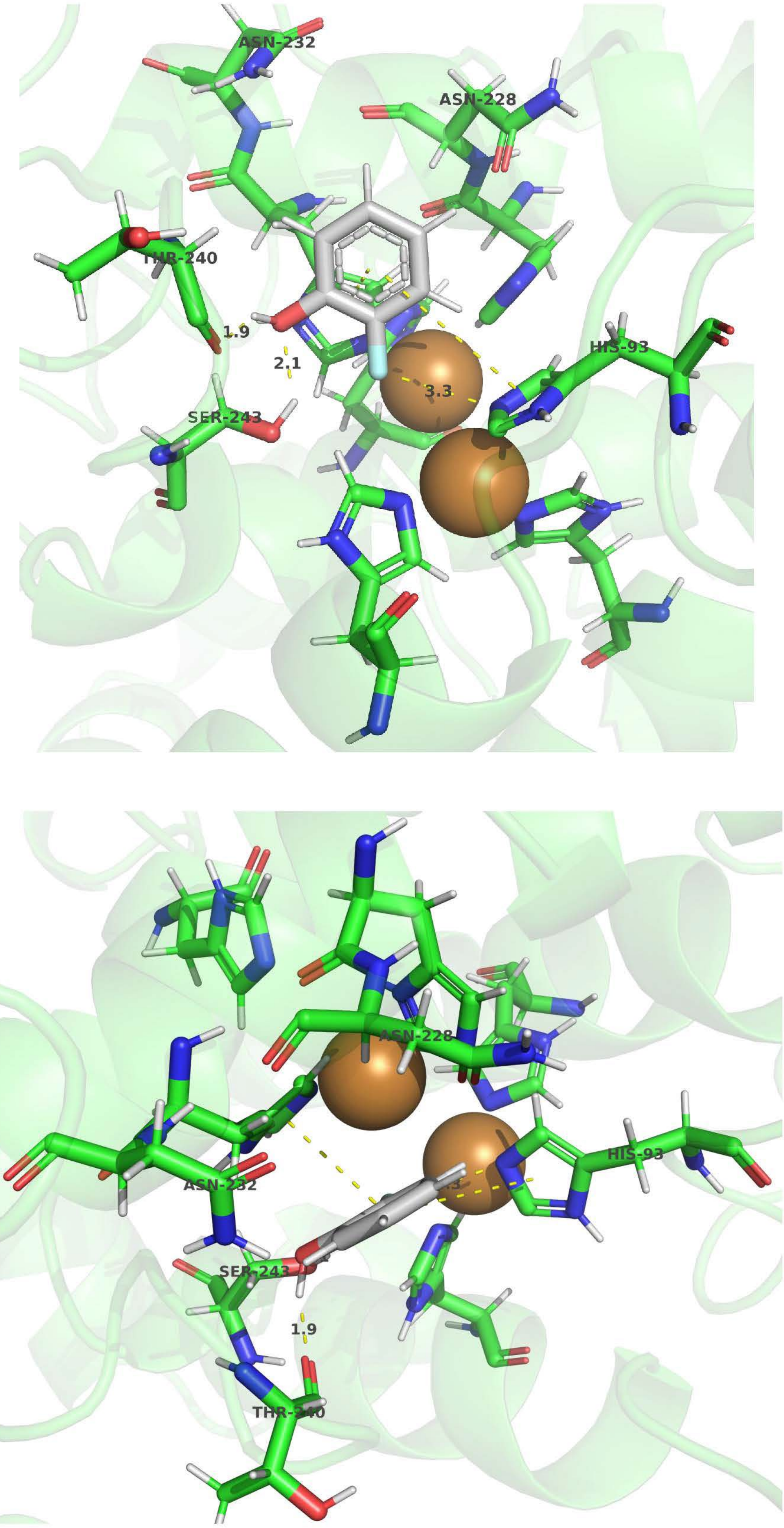

Figure S17. Top scoring SWISS-MODEL RsTyr ensemble docking solution for 2-flurophenol. A profile (top) and top-down (bottom) view is provided. In both images green ribbons depict the catalytic domain of the enzyme. 2-fluorophenol is depicted as grey stick models. In both models the 6 Cu coordinating histidines in the tyrosinase active site, the two activity controller residues (N228 and N232) and other residues which the substrate interacts with are depicted as stick models. Cu ions are brown/copper colour spheres. Yellow dotted lines depict interactions between residues, substrates and cofactors. Numbers on the yellow lines indicate the distance of the bond in angstroms (Å).

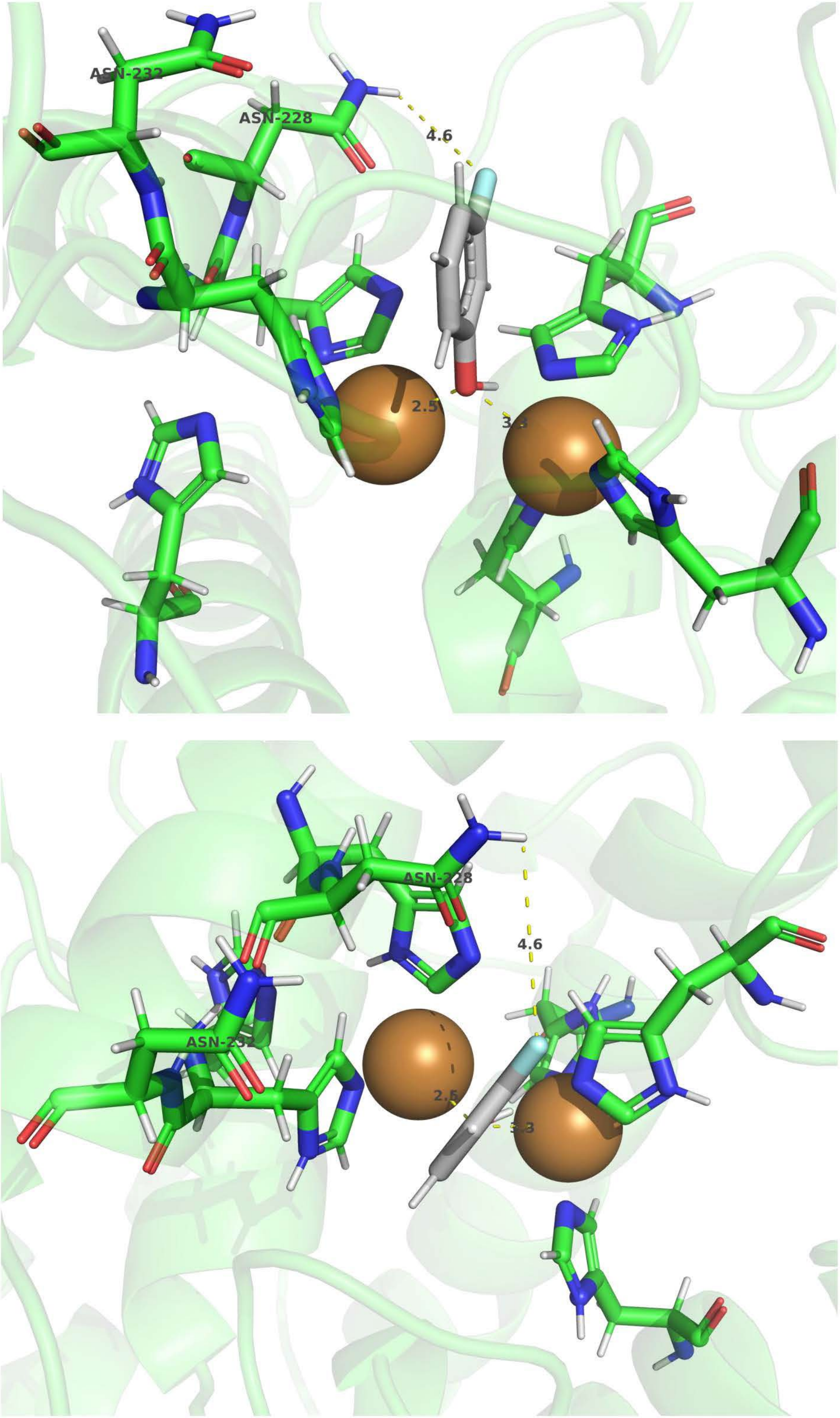

Figure S18. Top scoring SWISS-MODEL RsTyr ensemble docking solution for 3-fluorophenol. A profile (top) and top-down (bottom) view is provided. In both images green ribbons depict the catalytic domain of the enzyme. 3-fluorophenol is depicted as grey stick models. In both models the 6 Cu coordinating histidines in the tyrosinase active site, the two activity controller residues (N228 and N232) and other residues which the substrate interacts with are depicted as stick models. Cu ions are brown/copper colour spheres. Yellow dotted lines depict interactions between residues, substrates and cofactors. Numbers on the yellow lines indicate the distance of the bond in angstroms (Å).

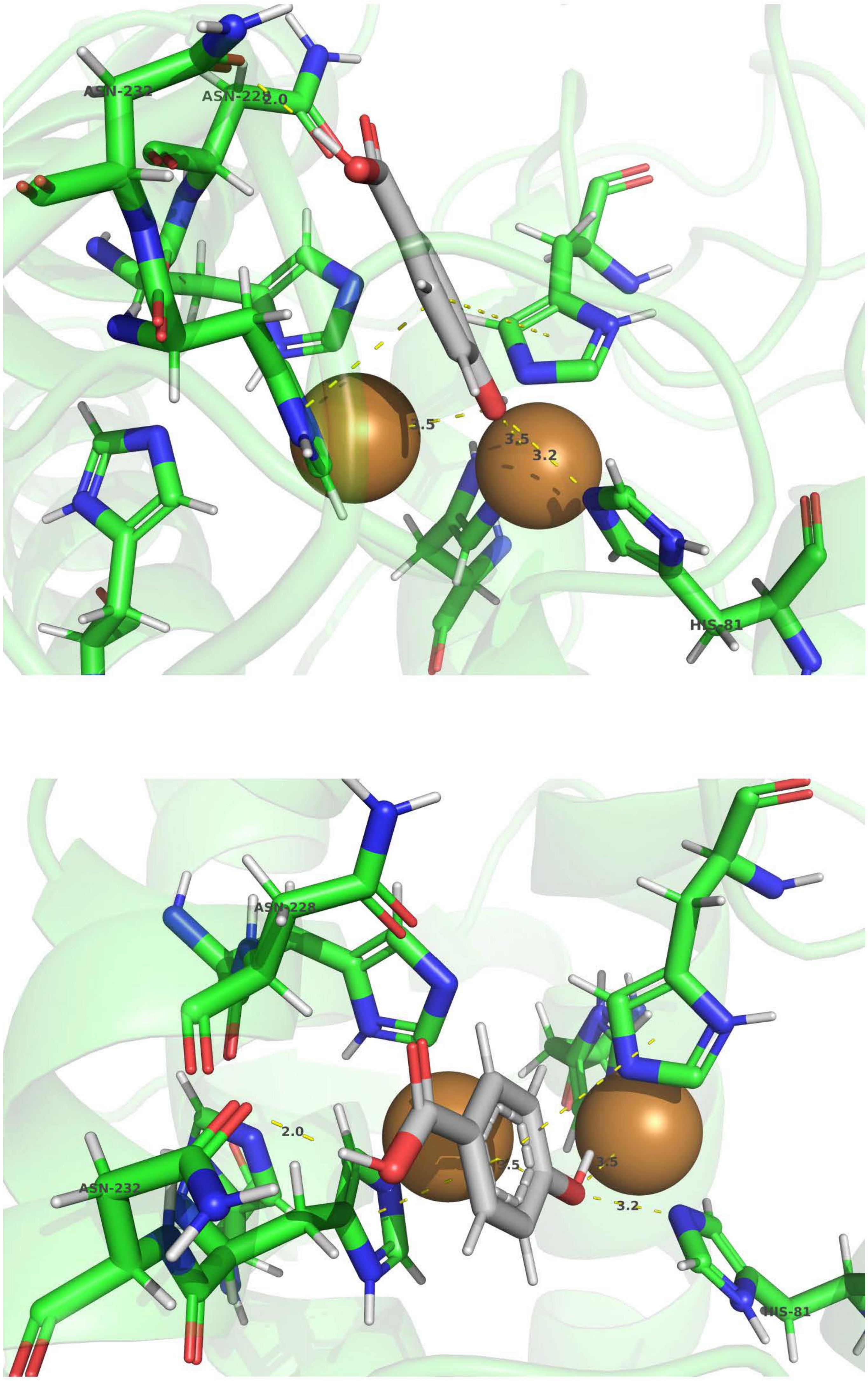

Figure S19. Top scoring SWISS-MODEL RsTyr ensemble docking solution for 4-hydroxybenzoic acid. A profile (top) and top-down (bottom) view is provided. In both images green ribbons depict the catalytic domain of the enzyme. 4-hydroxybenzoic acid is depicted as grey stick models. In both models the 6 Cu coordinating histidines in the tyrosinase active site, the two activity controller residues (N228 and N232) and other residues which the substrate interacts with are depicted as stick models. Cu ions are brown/copper colour spheres. Yellow dotted lines depict interactions between residues, substrates and cofactors. Numbers on the yellow lines indicate the distance of the bond in angstroms (Å).

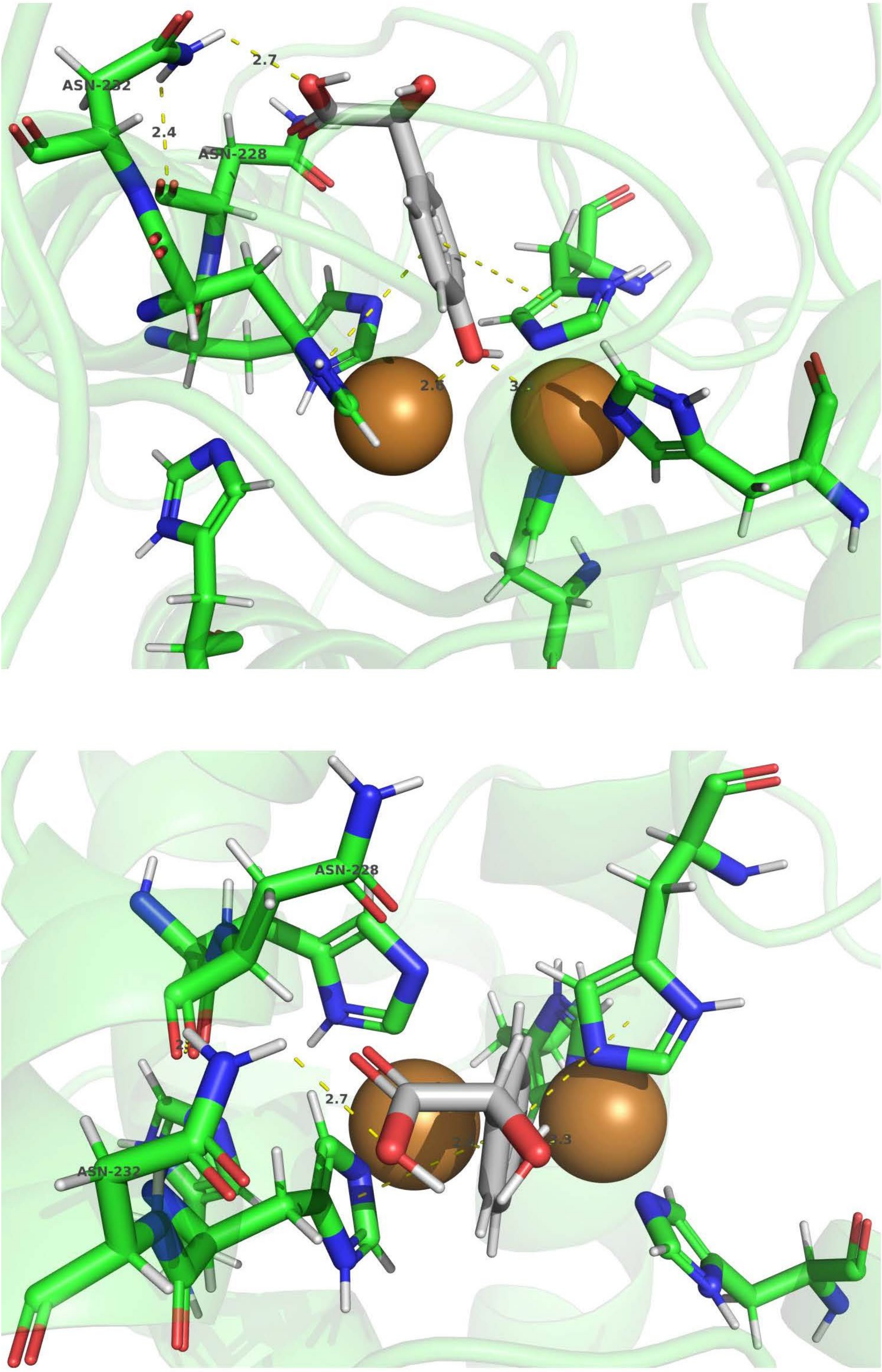

Figure S20. Top scoring SWISS-MODEL RsTyr ensemble docking solution for 4-hydroxymandelic acid. A profile (top) and top-down (bottom) view is provided. In both images green ribbons depict the catalytic domain of the enzyme. 4-hydroxymandelic acid is depicted as grey stick models. In both models the 6 Cu coordinating histidines in the tyrosinase active site, the two activity controller residues (N228 and N232) and other residues which the substrate interacts with are depicted as stick models. Cu ions are brown/copper colour spheres. Yellow dotted lines depict interactions between residues, substrates and cofactors. Numbers on the yellow lines indicate the distance of the bond in angstroms (Å).

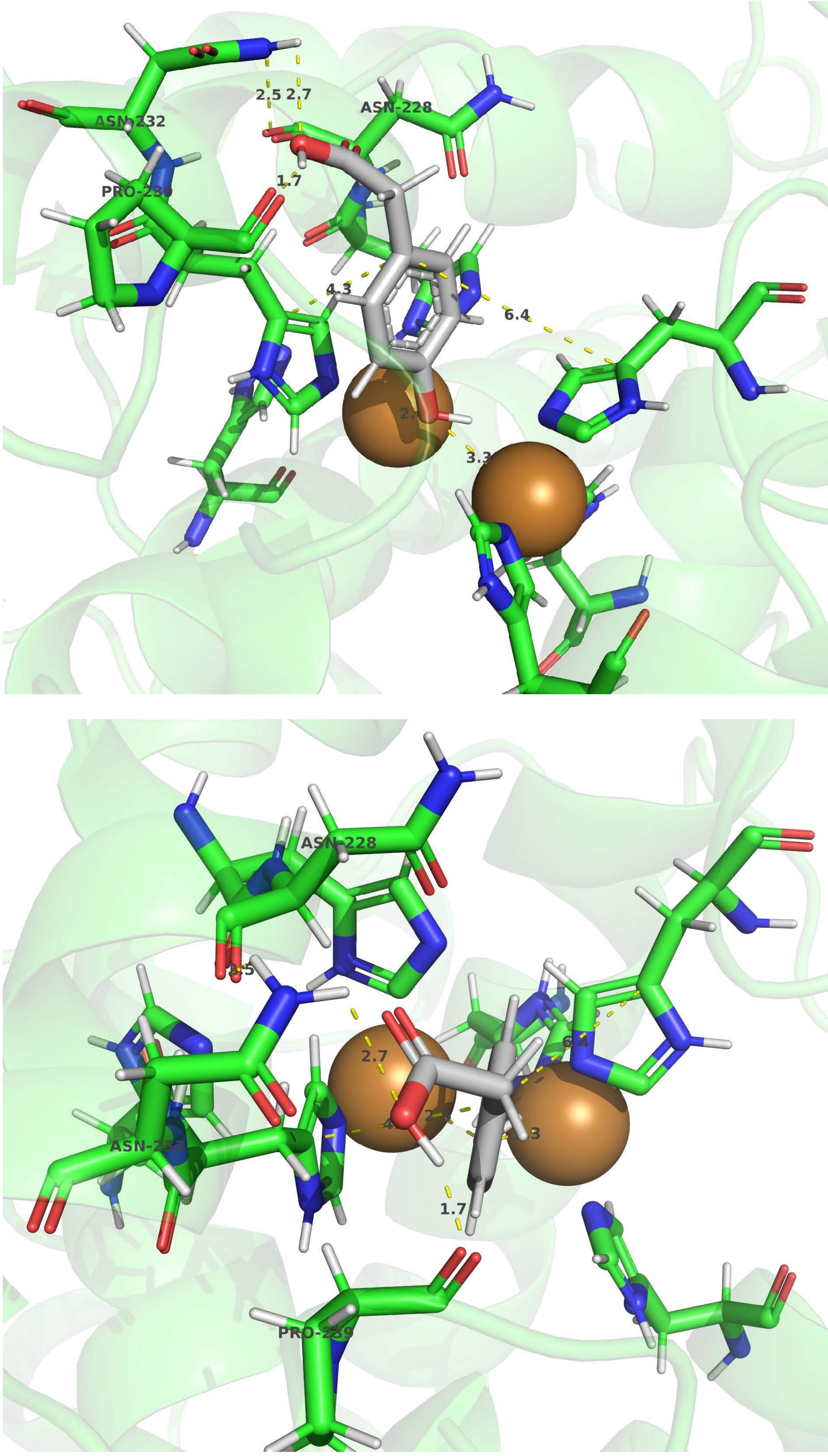

Figure S21. Top scoring SWISS-MODEL RsTyr ensemble docking solution for 4-hydroxyphenylacetic acid. A profile (top) and top-down (bottom) view is provided. In both images green ribbons depict the catalytic domain of the enzyme. The substrate is depicted as grey stick models. In both models the 6 Cu coordinating histidines in the tyrosinase active site, the two activity controller residues (N228 and N232) and other residues which the substrate interacts with are depicted as stick models. Cu ions are brown/copper colour spheres. Yellow dotted lines depict interactions between residues, substrates and cofactors. Numbers on the yellow lines indicate the distance of the bond in angstroms (Å).

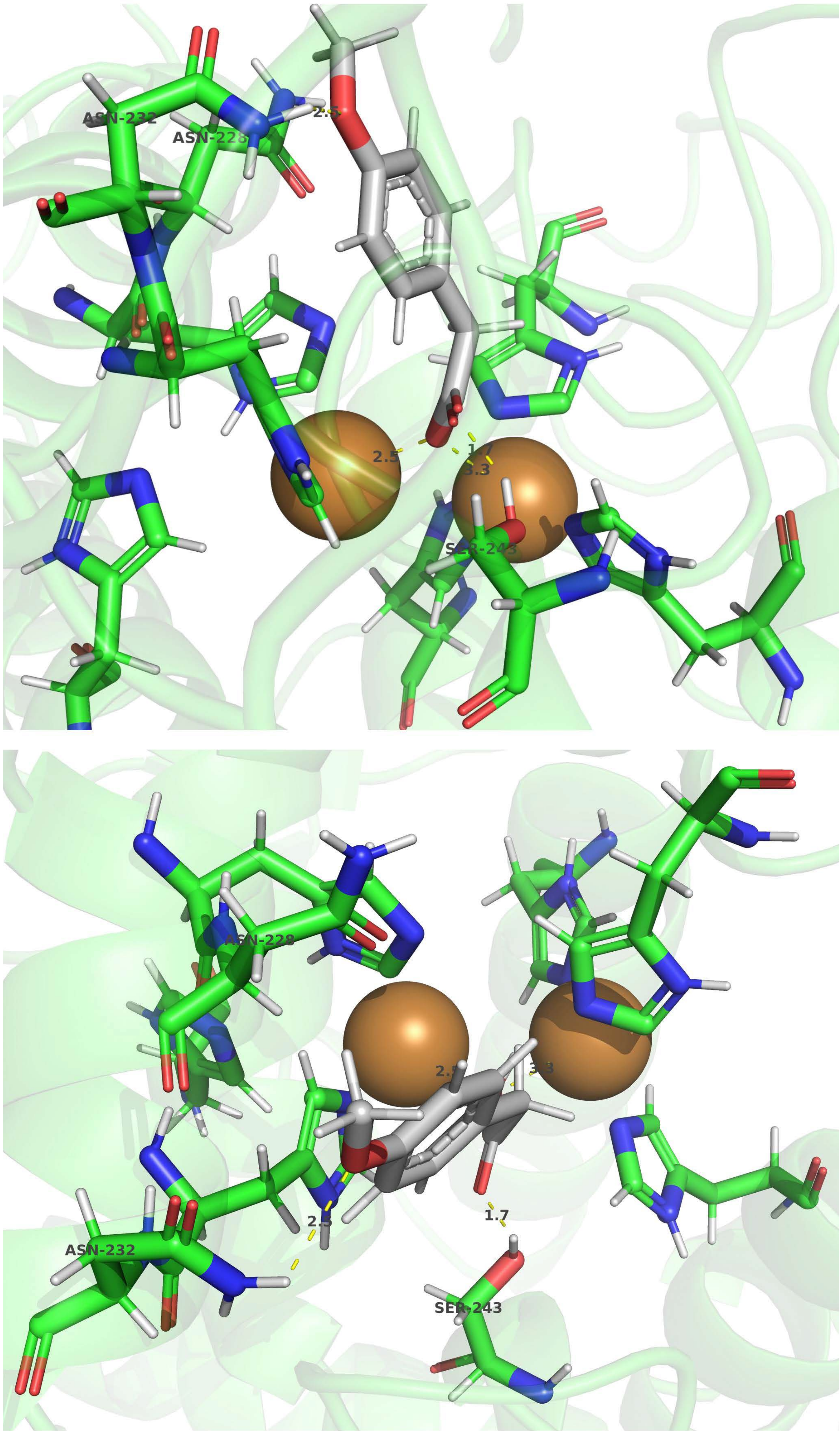

Figure S22. Top scoring SWISS-MODEL RsTyr ensemble docking solution for 4-methoxyphenylacetic acid. In both images green ribbons depict the catalytic domain of the enzyme. The substrate is depicted as grey stick models. In both models the 6 Cu coordinating histidines in the tyrosinase active site, the two activity controller residues (N228 and N232) and other residues which the substrate interacts with are depicted as stick models. Cu ions are brown/copper colour spheres. Yellow dotted lines depict interactions between residues, substrates and cofactors. Numbers on the yellow lines indicate the distance of the bond in angstroms (Å).

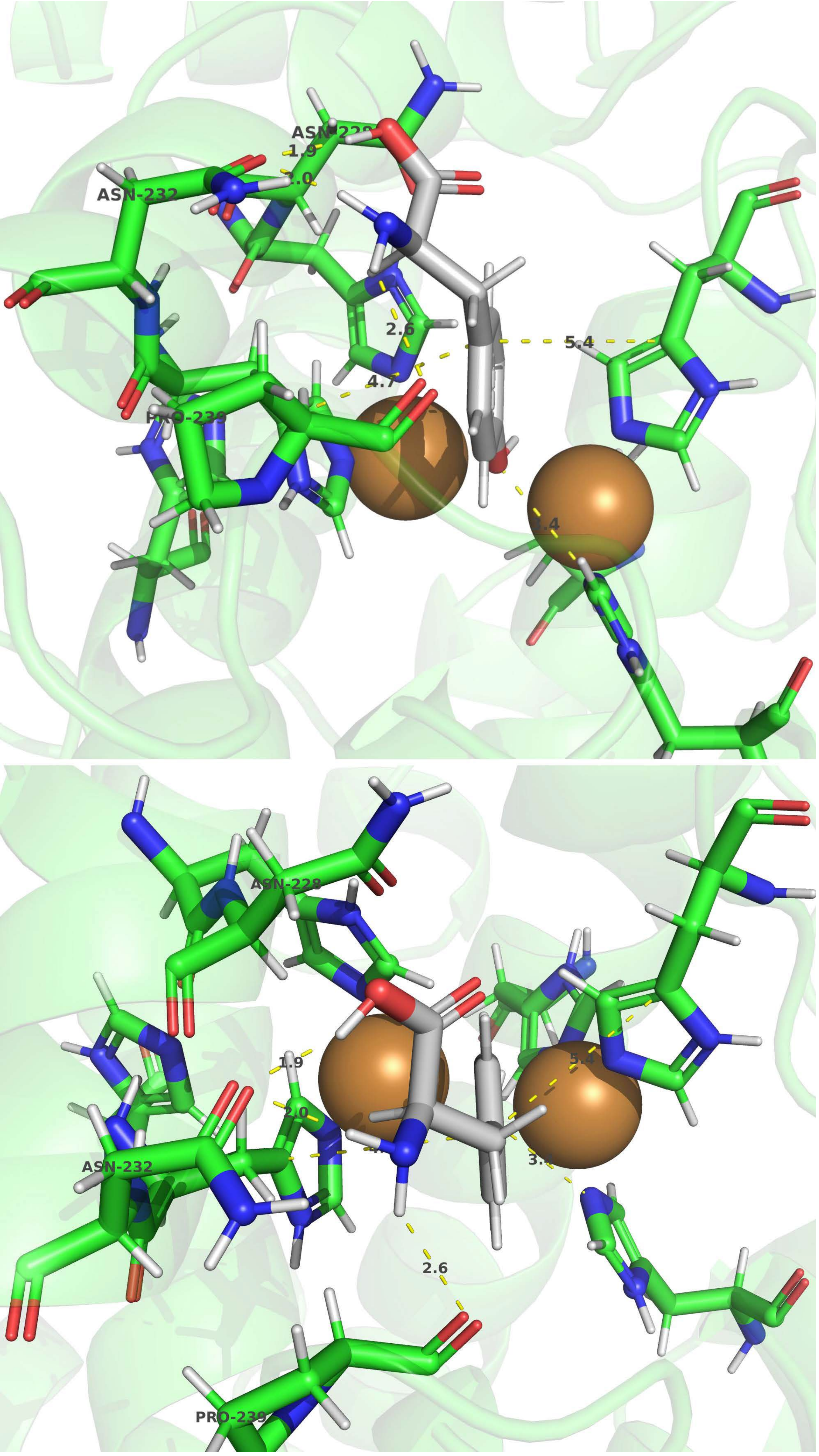

Figure S23. Top scoring AlphaFold RsTyr model docking solution for Tyrosine. A profile (top) and top-down (bottom) view for each substrate is provided. In all images the green ribbons depict the catalytic domain of the enzyme. The substrate is depicted as grey stick models. In all models the 6 Cu coordinating histidines in the tyrosinase active site, the two activity controller residues (N228 and N232) and other residues which the substrate interacts with are depicted as stick models. Cu ions are brown/copper colour spheres. Yellow dotted lines depict interactions between residues, substrates and cofactors. Numbers on the yellow lines indicate the distance of the bond in angstroms (Å).

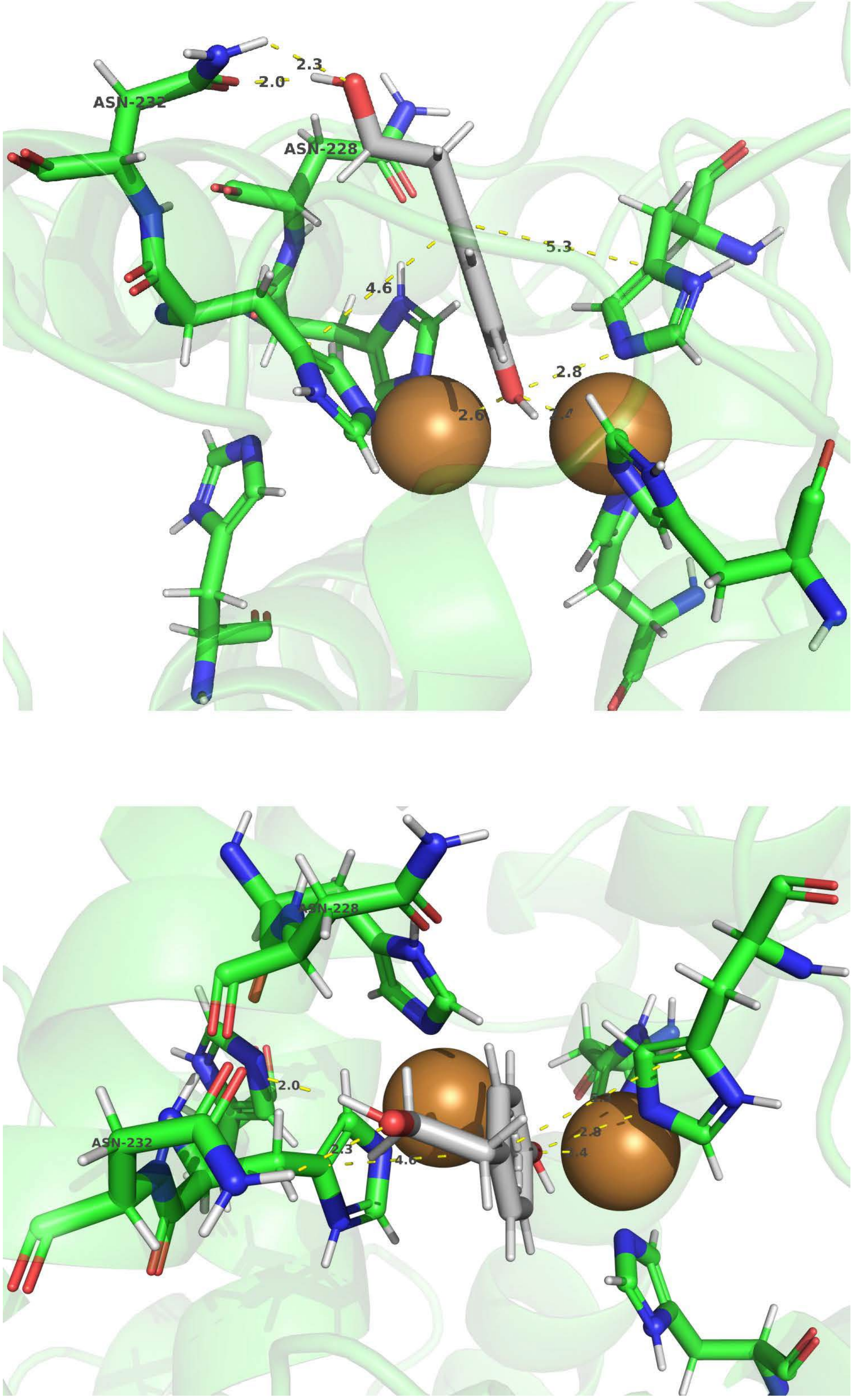

Figure S24. Top scoring AlphaFold RsTyr model docking solution for Tyrosol. A profile (top) and top-down (bottom) view for each substrate is provided. In all images the green ribbons depict the catalytic domain of the enzyme. The substrate is depicted as grey stick models. In all models the 6 Cu coordinating histidines in the tyrosinase active site, the two activity controller residues (N228 and N232) and other residues which the substrate interacts with are depicted as stick models. Cu ions are brown/copper colour spheres. Yellow dotted lines depict interactions between residues, substrates and cofactors. Numbers on the yellow lines indicate the distance of the bond in angstroms (Å).

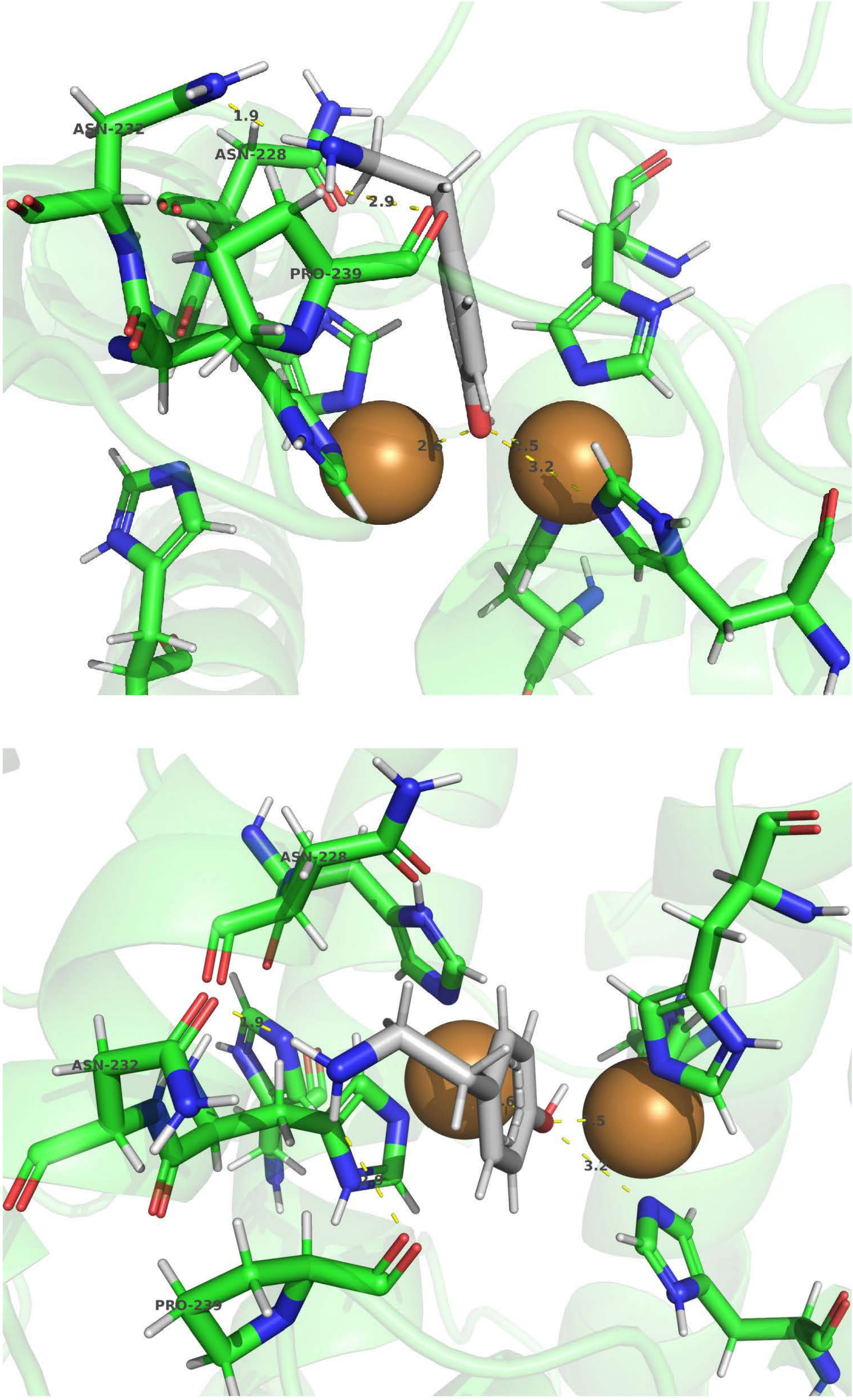

Figure S25. Top scoring AlphaFold RsTyr model docking solution for Tyramine. A profile (top) and top-down (bottom) view for each substrate is provided. In all images the green ribbons depict the catalytic domain of the enzyme. The substrate is depicted as grey stick models. In all models the 6 Cu coordinating histidines in the tyrosinase active site, the two activity controller residues (N228 and N232) and other residues which the substrate interacts with are depicted as stick models. Cu ions are brown/copper colour spheres. Yellow dotted lines depict interactions between residues, substrates and cofactors. Numbers on the yellow lines indicate the distance of the bond in angstroms (Å).

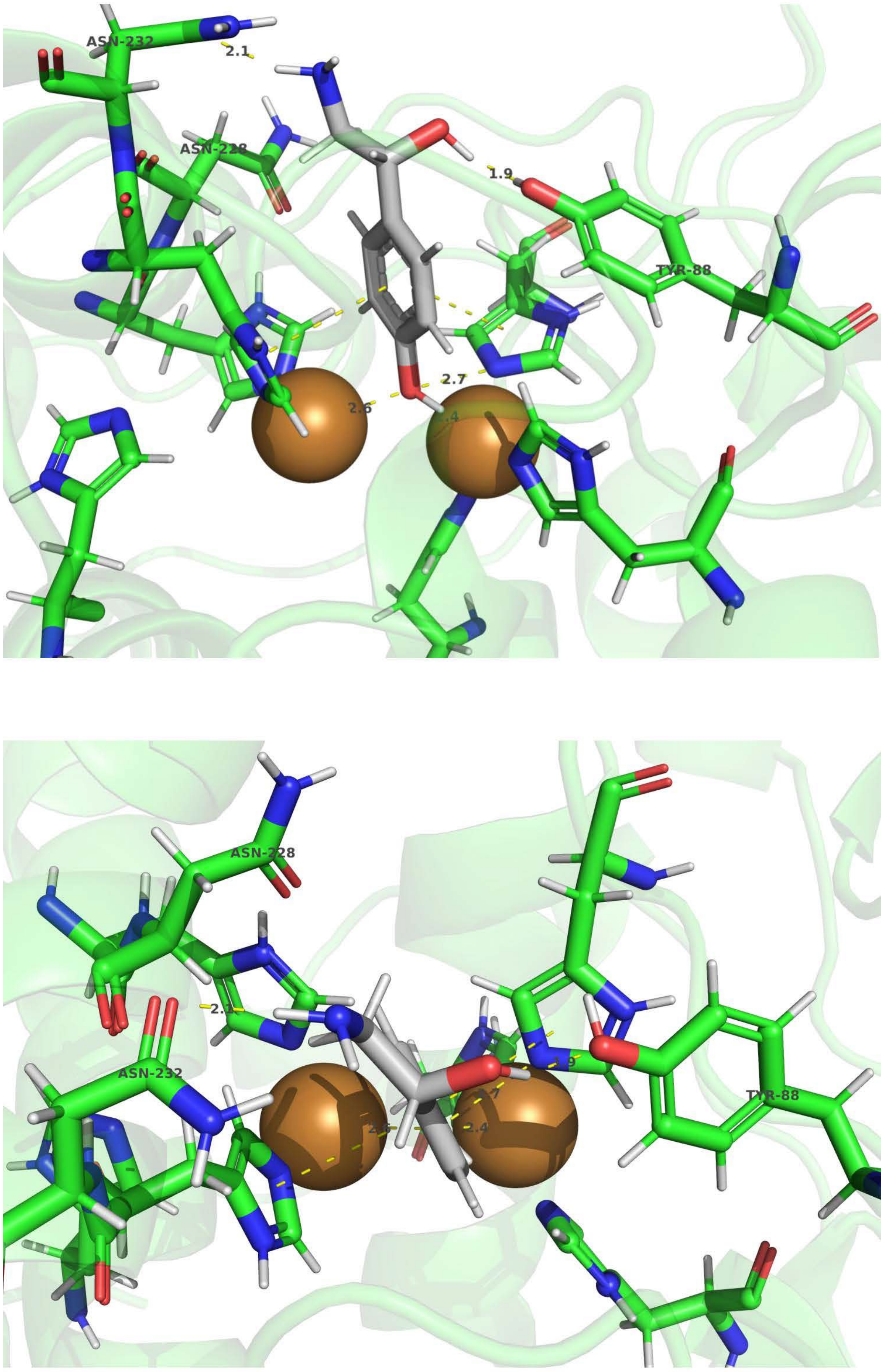

Figure S26. Top scoring AlphaFold RsTyr model docking solution for Octopamine. A profile (top) and top-down (bottom) view for each substrate is provided. In all images the green ribbons depict the catalytic domain of the enzyme. The substrate is depicted as grey stick models. In all models the 6 Cu coordinating histidines in the tyrosinase active site, the two activity controller residues (N228 and N232) and other residues which the substrate interacts with are depicted as stick models. Cu ions are brown/copper colour spheres. Yellow dotted lines depict interactions between residues, substrates and cofactors. Numbers on the yellow lines indicate the distance of the bond in angstroms (Å).

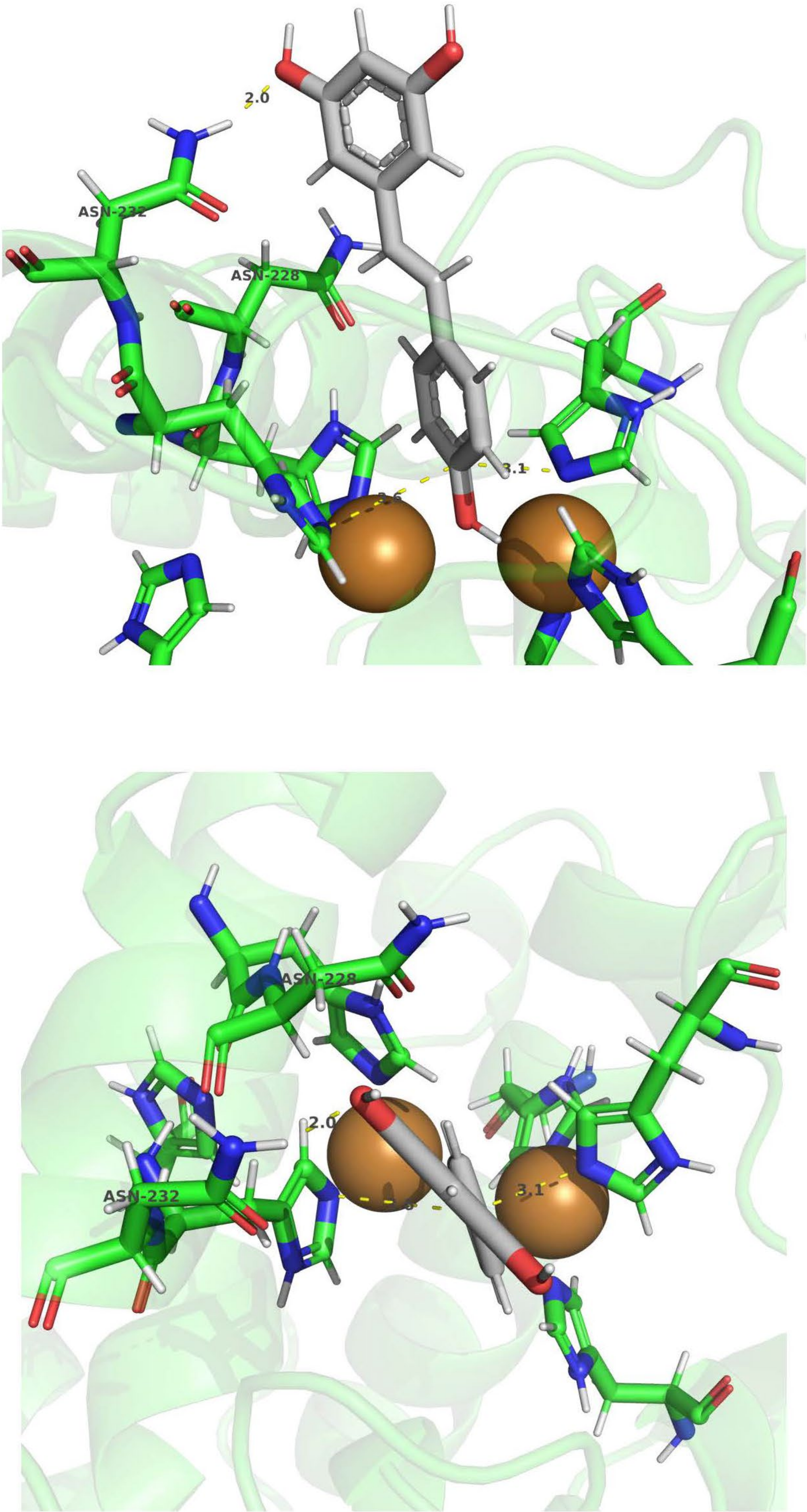

Figure S27. Top scoring AlphaFold RsTyr model docking solution for Resveratrol. A profile (top) and top-down (bottom) view for each substrate is provided. In all images the green ribbons depict the catalytic domain of the enzyme. The substrate is depicted as grey stick models. In all models the 6 Cu coordinating histidines in the tyrosinase active site, the two activity controller residues (N228 and N232) and other residues which the substrate interacts with are depicted as stick models. Cu ions are brown/copper colour spheres. Yellow dotted lines depict interactions between residues, substrates and cofactors. Numbers on the yellow lines indicate the distance of the bond in angstroms (Å).

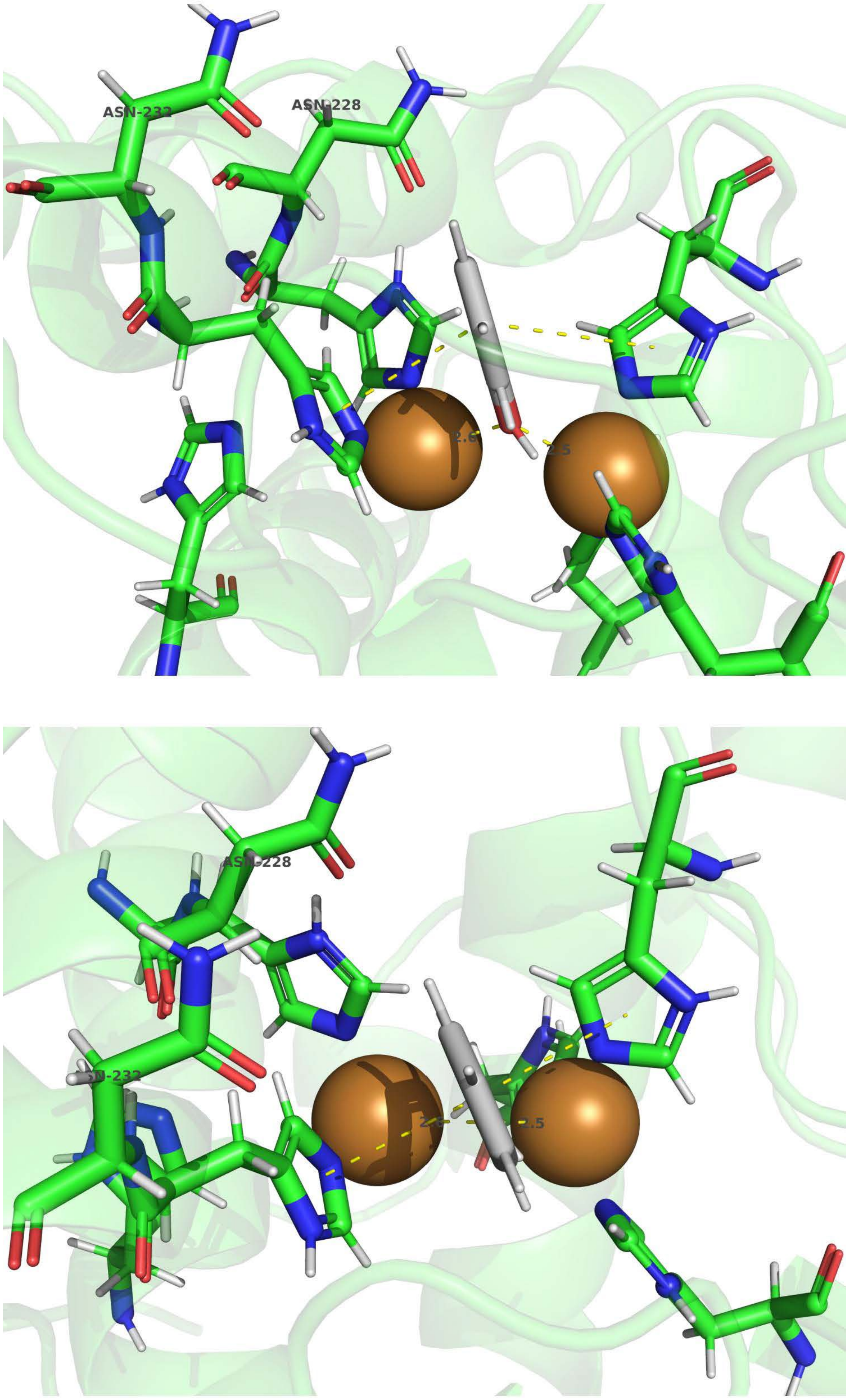

Figure S28. Top scoring AlphaFold RsTyr model docking solution for Phenol. A profile (top) and top-down (bottom) view for each substrate is provided. In all images the green ribbons depict the catalytic domain of the enzyme. The substrate is depicted as grey stick models. In all models the 6 Cu coordinating histidines in the tyrosinase active site, the two activity controller residues (N228 and N232) and other residues which the substrate interacts with are depicted as stick models. Cu ions are brown/copper colour spheres. Yellow dotted lines depict interactions between residues, substrates and cofactors. Numbers on the yellow lines indicate the distance of the bond in angstroms (Å).

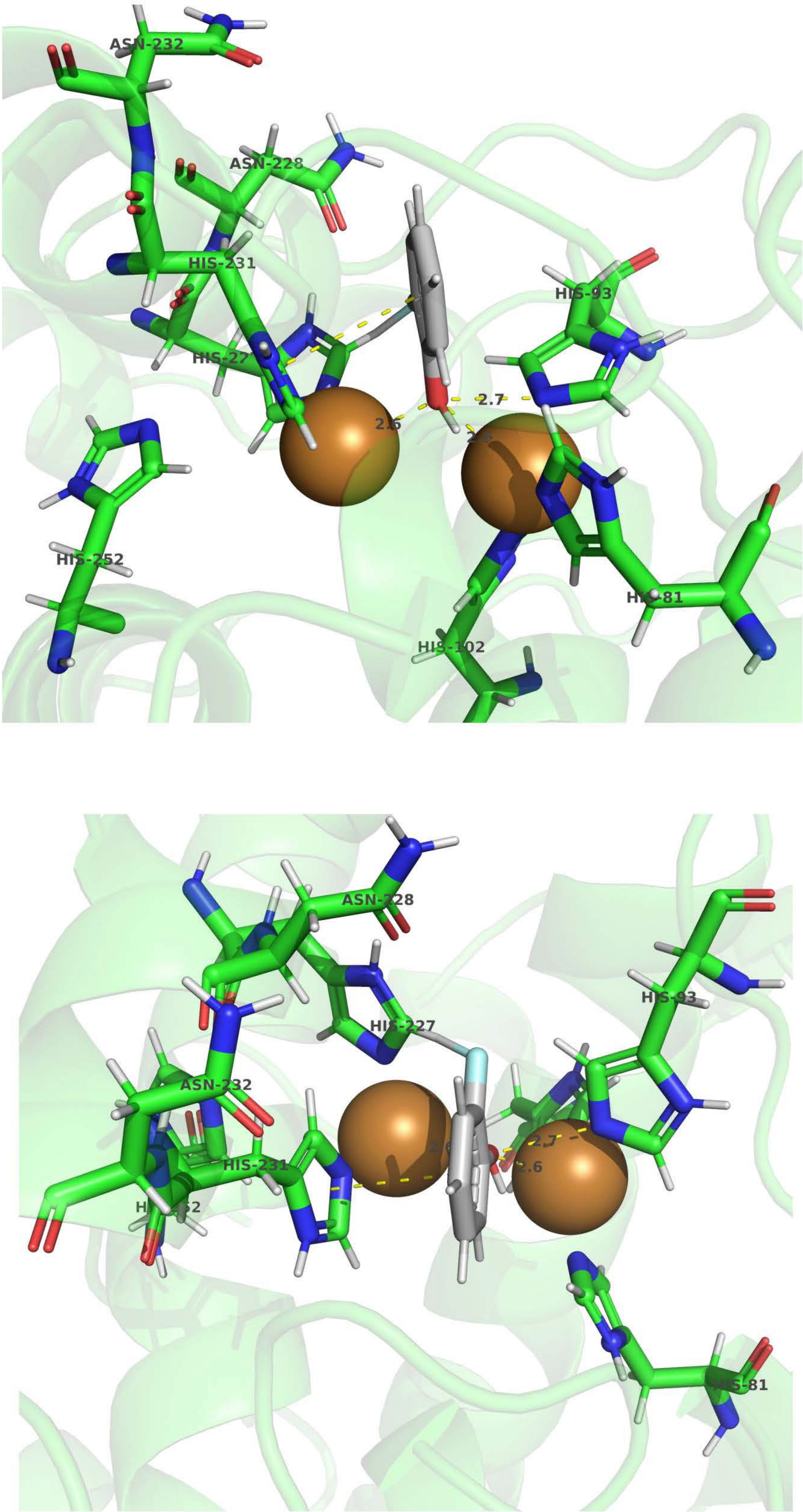

Figure S29. Top scoring AlphaFold RsTyr model docking solution for 2-fluorophenol. A profile (top) and top-down (bottom) view for each substrate is provided. In all images the green ribbons depict the catalytic domain of the enzyme. The substrate is depicted as grey stick models. In all models the 6 Cu coordinating histidines in the tyrosinase active site, the two activity controller residues (N228 and N232) and other residues which the substrate interacts with are depicted as stick models. Cu ions are brown/copper colour spheres. Yellow dotted lines depict interactions between residues, substrates and cofactors. Numbers on the yellow lines indicate the distance of the bond in angstroms (Å).

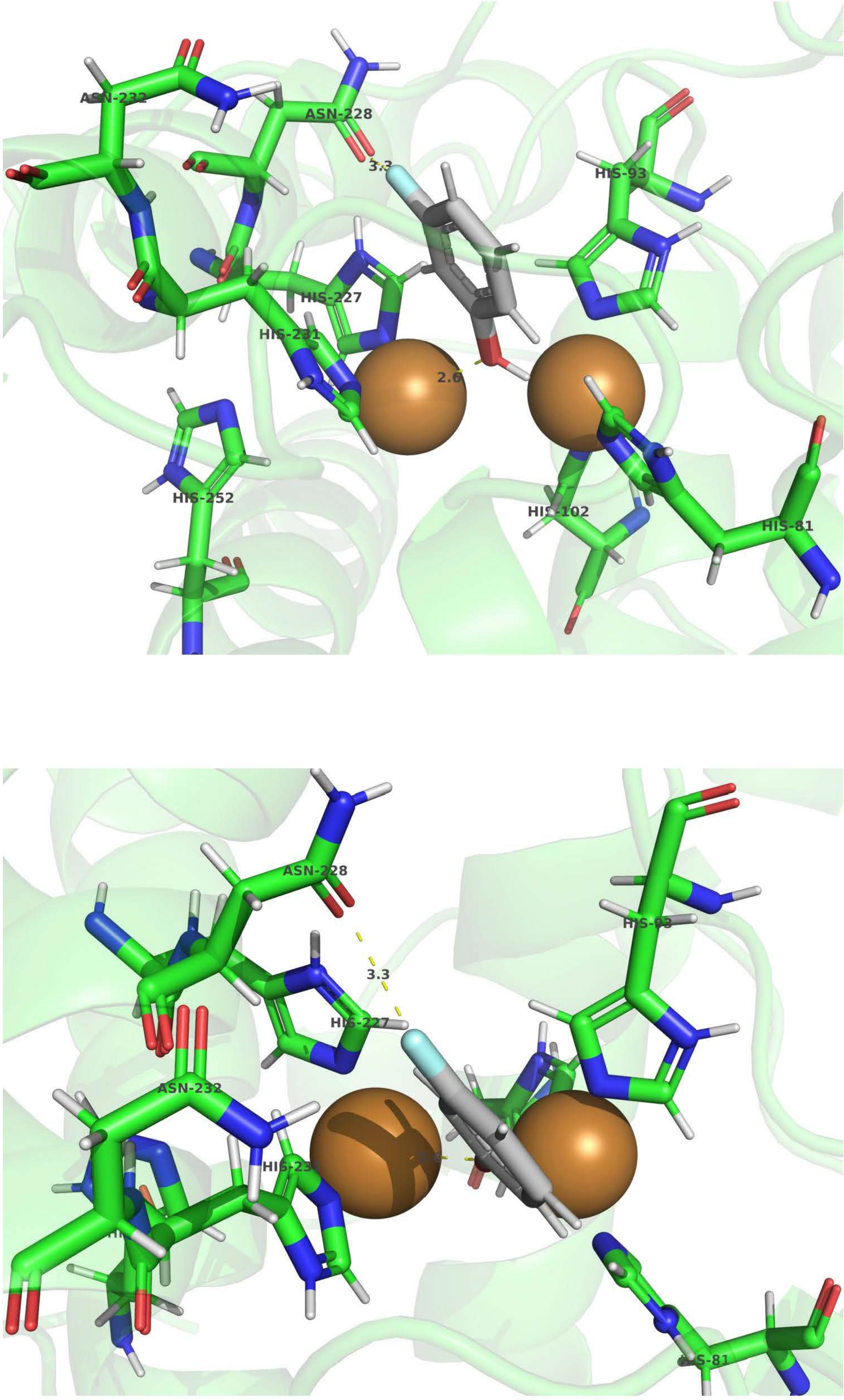

Figure S30. Top scoring AlphaFold RsTyr model docking solution for 3-fluorophenol. A profile (top) and top-down (bottom) view for each substrate is provided. In all images the green ribbons depict the catalytic domain of the enzyme. The substrate is depicted as grey stick models. In all models the 6 Cu coordinating histidines in the tyrosinase active site, the two activity controller residues (N228 and N232) and other residues which the substrate interacts with are depicted as stick models. Cu ions are brown/copper colour spheres. Yellow dotted lines depict interactions between residues, substrates and cofactors. Numbers on the yellow lines indicate the distance of the bond in angstroms (Å).

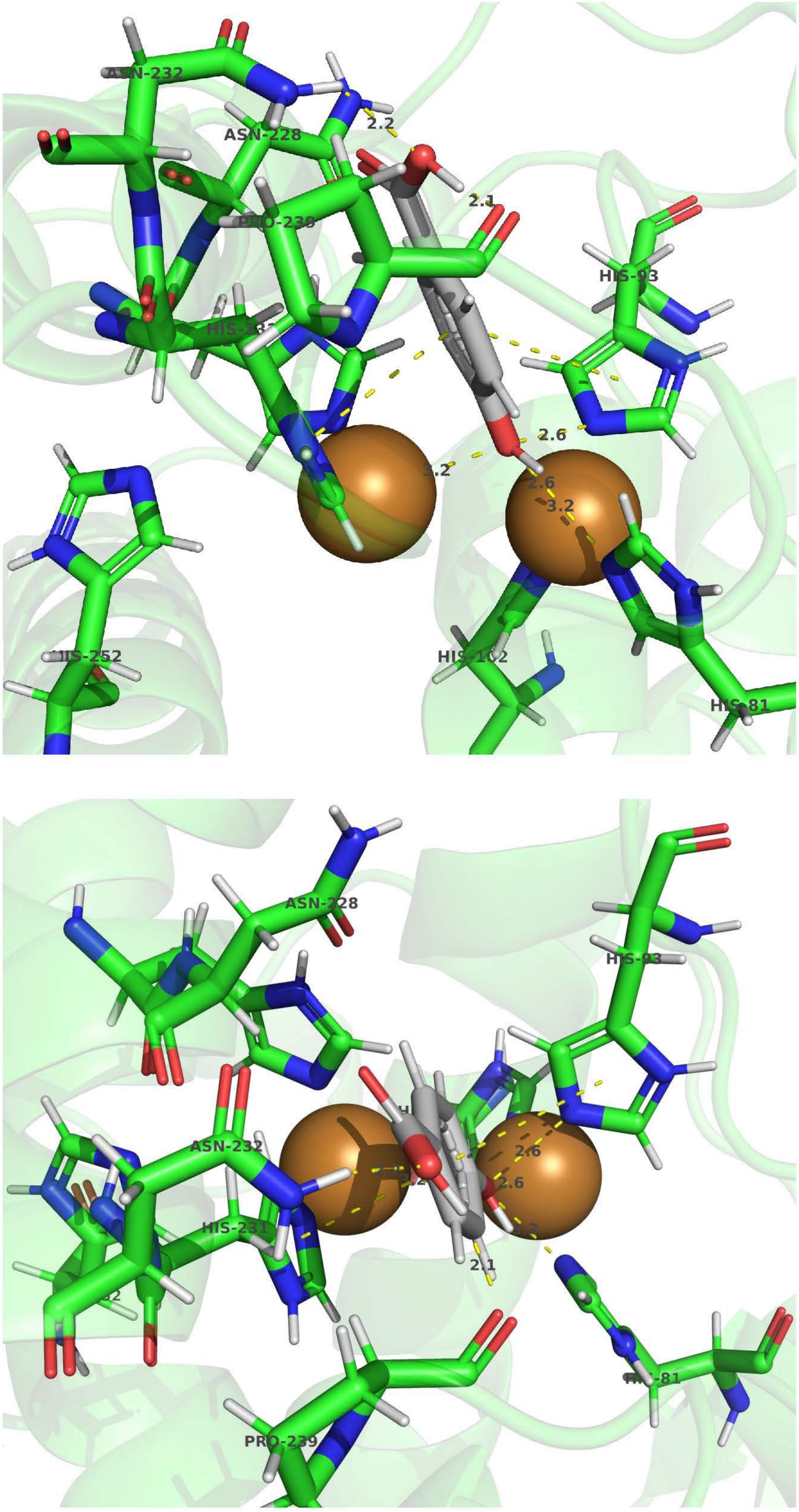

Figure S31. Top scoring AlphaFold RsTyr model docking solution for 4-hydroxybenzoic acid. A profile (top) and top-down (bottom) view for each substrate is provided. In all images the green ribbons depict the catalytic domain of the enzyme. The substrate is depicted as grey stick models. In all models the 6 Cu coordinating histidines in the tyrosinase active site, the two activity controller residues (N228 and N232) and other residues which the substrate interacts with are depicted as stick models. Cu ions are brown/copper colour spheres. Yellow dotted lines depict interactions between residues, substrates and cofactors. Numbers on the yellow lines indicate the distance of the bond in angstroms (Å).

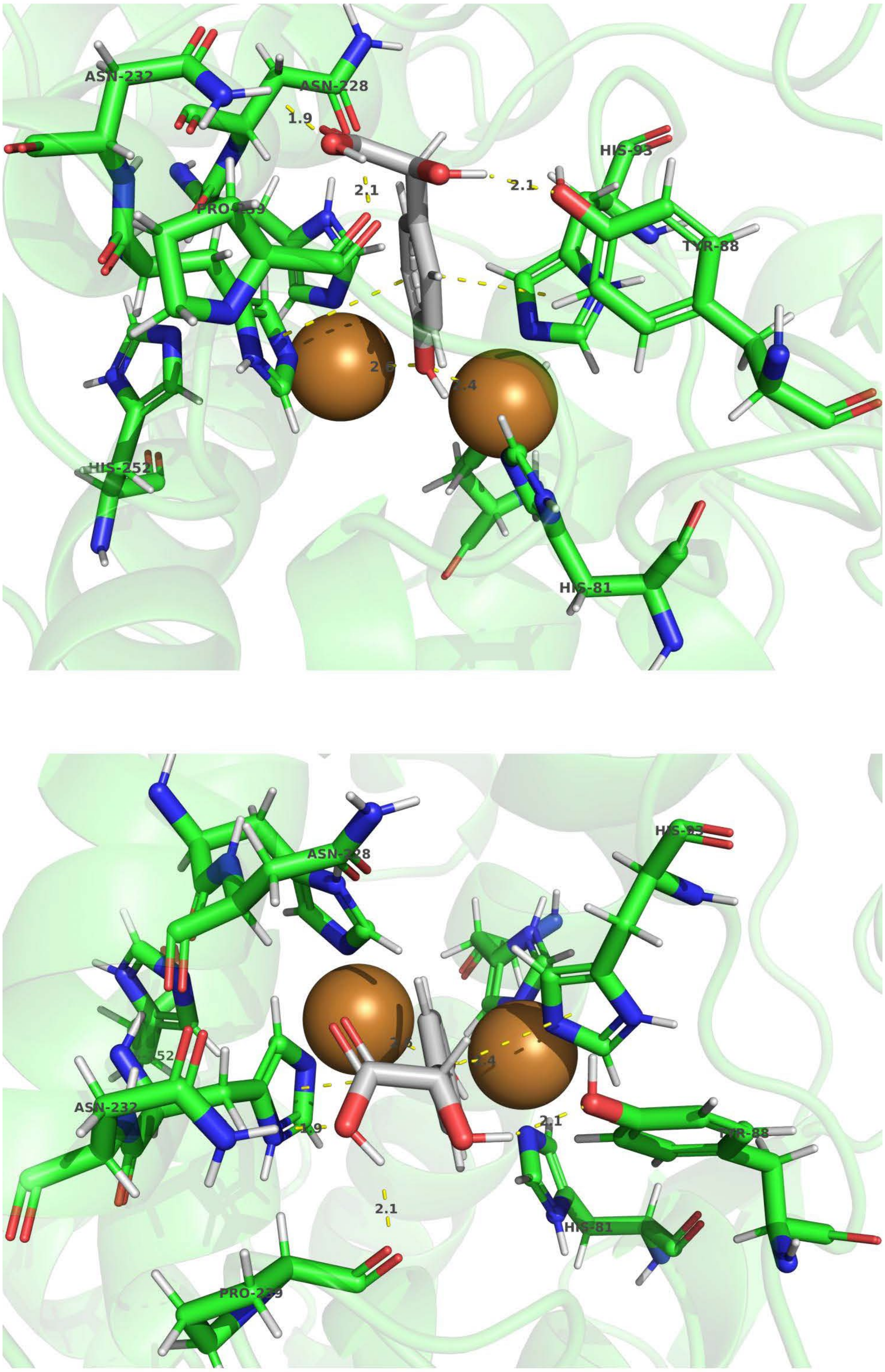

Figure S32. Top scoring AlphaFold RsTyr model docking solution for 4-hydroxymandelic acid. A profile (top) and top-down (bottom) view for each substrate is provided. In all images the green ribbons depict the catalytic domain of the enzyme. The substrate is depicted as grey stick models. In all models the 6 Cu coordinating histidines in the tyrosinase active site, the two activity controller residues (N228 and N232) and other residues which the substrate interacts with are depicted as stick models. Cu ions are brown/copper colour spheres. Yellow dotted lines depict interactions between residues, substrates and cofactors. Numbers on the yellow lines indicate the distance of the bond in angstroms (Å).

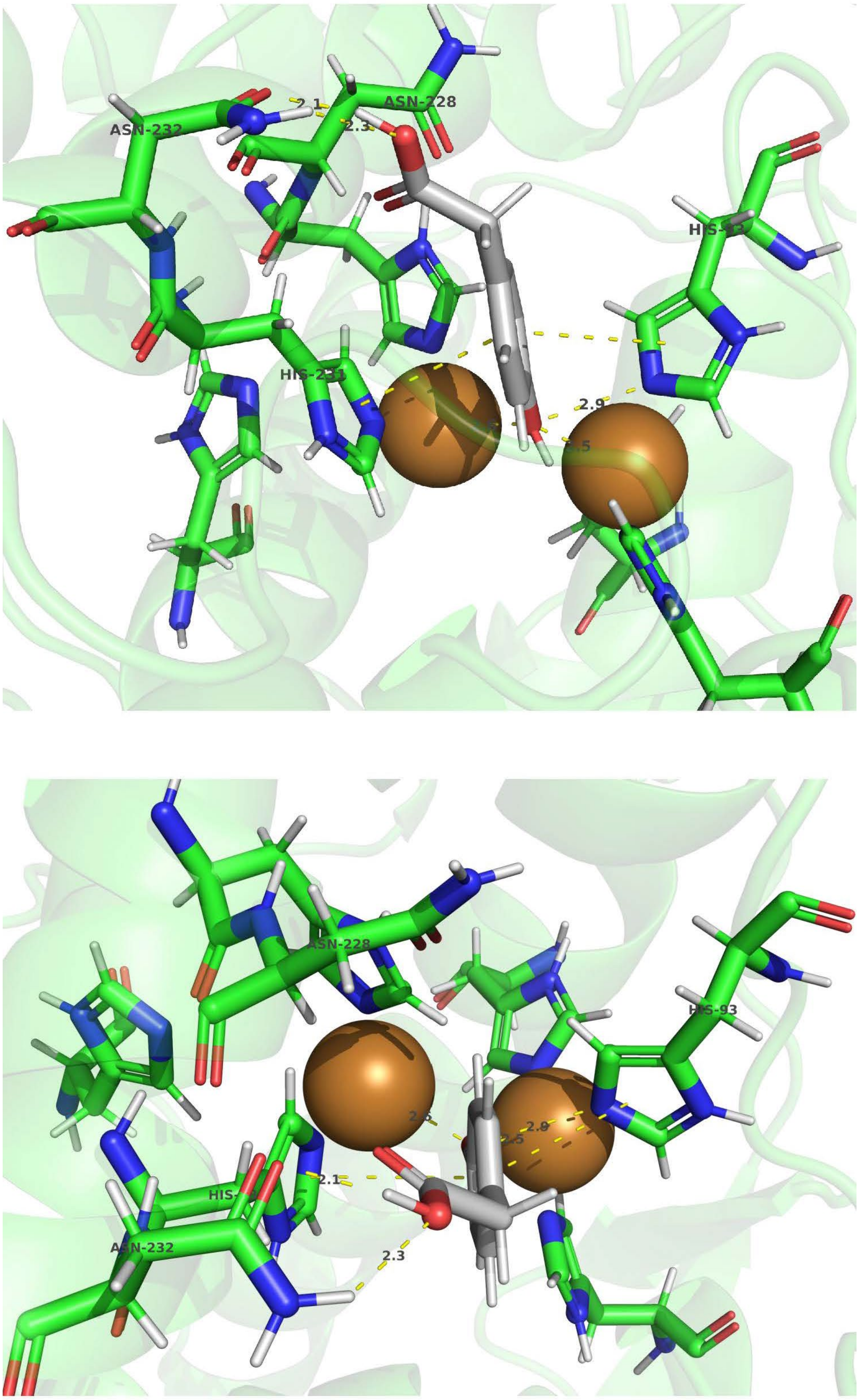

Figure S33. Top scoring AlphaFold RsTyr model docking solution for 4-hydroxyphenylacetic acid. A profile (top) and top-down (bottom) view for each substrate is provided. In all images the green ribbons depict the catalytic domain of the enzyme. The substrate is depicted as grey stick models. In all models the 6 Cu coordinating histidines in the tyrosinase active site, the two activity controller residues (N228 and N232) and other residues which the substrate interacts with are depicted as stick models. Cu ions are brown/copper colour spheres. Yellow dotted lines depict interactions between residues, substrates and cofactors. Numbers on the yellow lines indicate the distance of the bond in angstroms (Å).

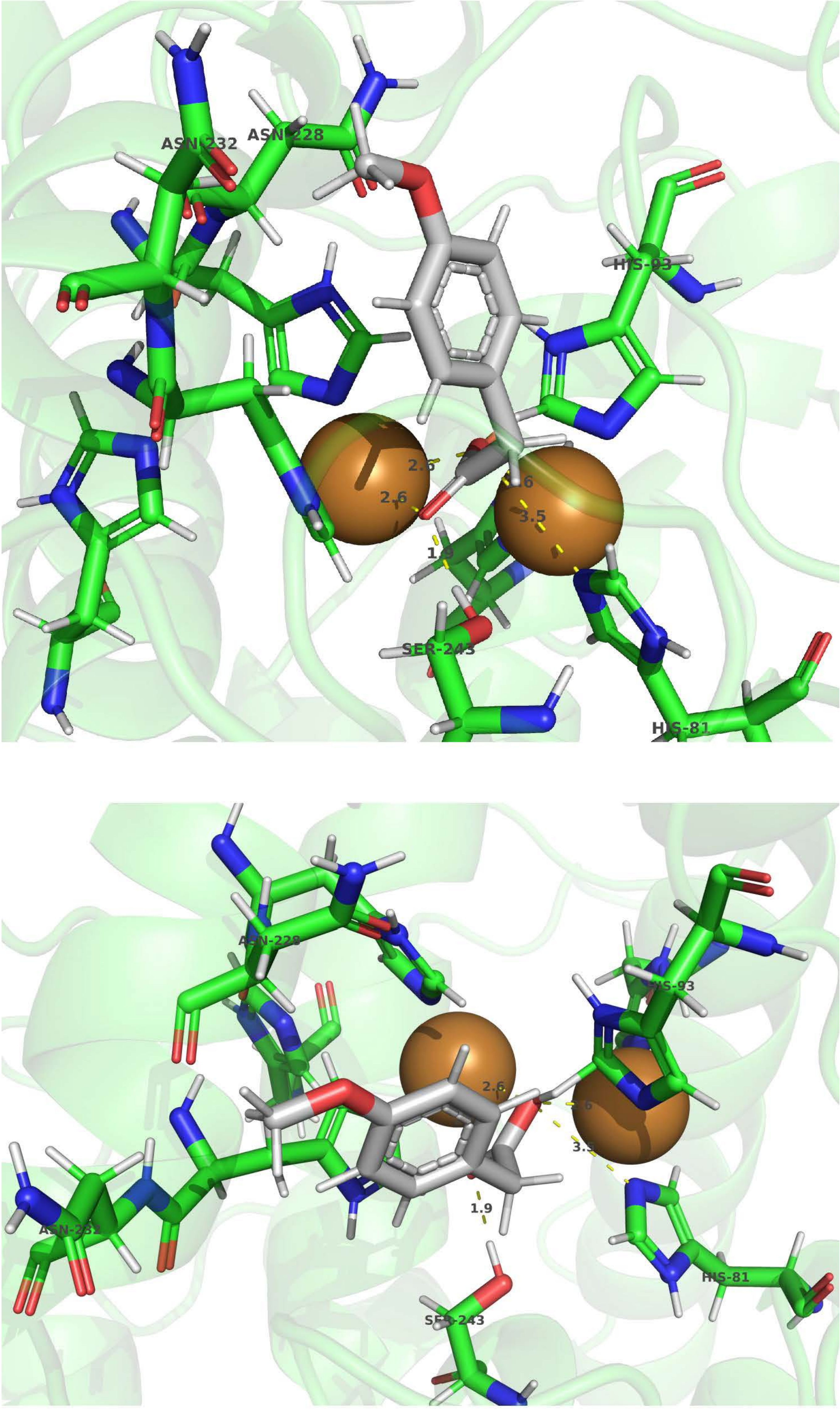

Figure S34. Top scoring AlphaFold RsTyr model docking solution for 4-methoxyphenylacetic acid. A profile (top) and top-down (bottom) view for each substrate is provided. In all images the green ribbons depict the catalytic domain of the enzyme. The substrate is depicted as grey stick models. In all models the 6 Cu coordinating histidines in the tyrosinase active site, the two activity controller residues (N228 and N232) and other residues which the substrate interacts with are depicted as stick models. Cu ions are brown/copper colour spheres. Yellow dotted lines depict interactions between residues, substrates and cofactors. Numbers on the yellow lines indicate the distance of the bond in angstroms (Å).

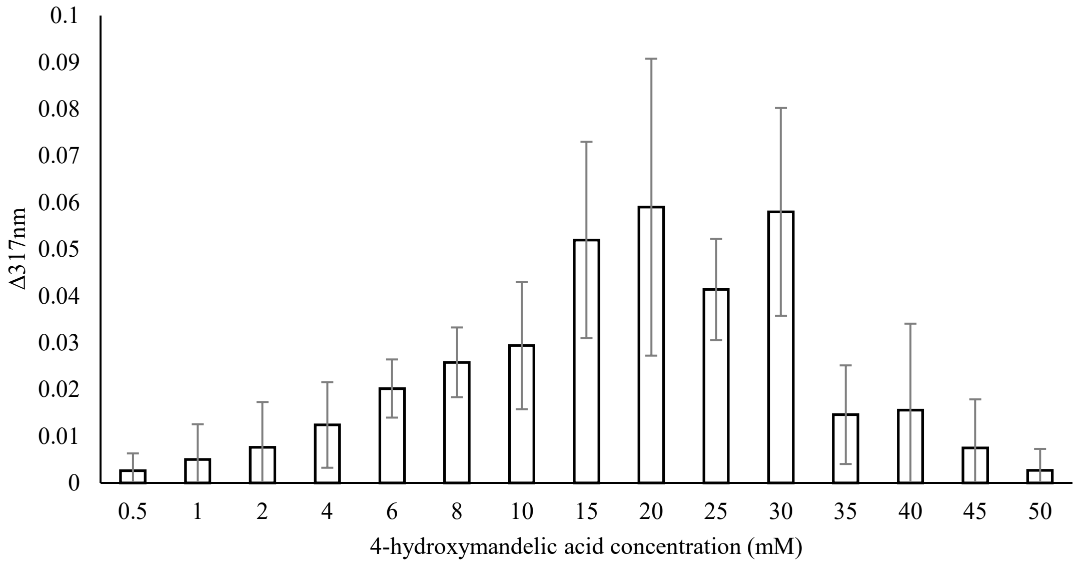

Figure S35. 4-hydroxymandelic acid Δ317 absorption change in a 1-hour kinetic assay with RsTyr at 317 nm. Error bars indicate one standard deviation from the mean.

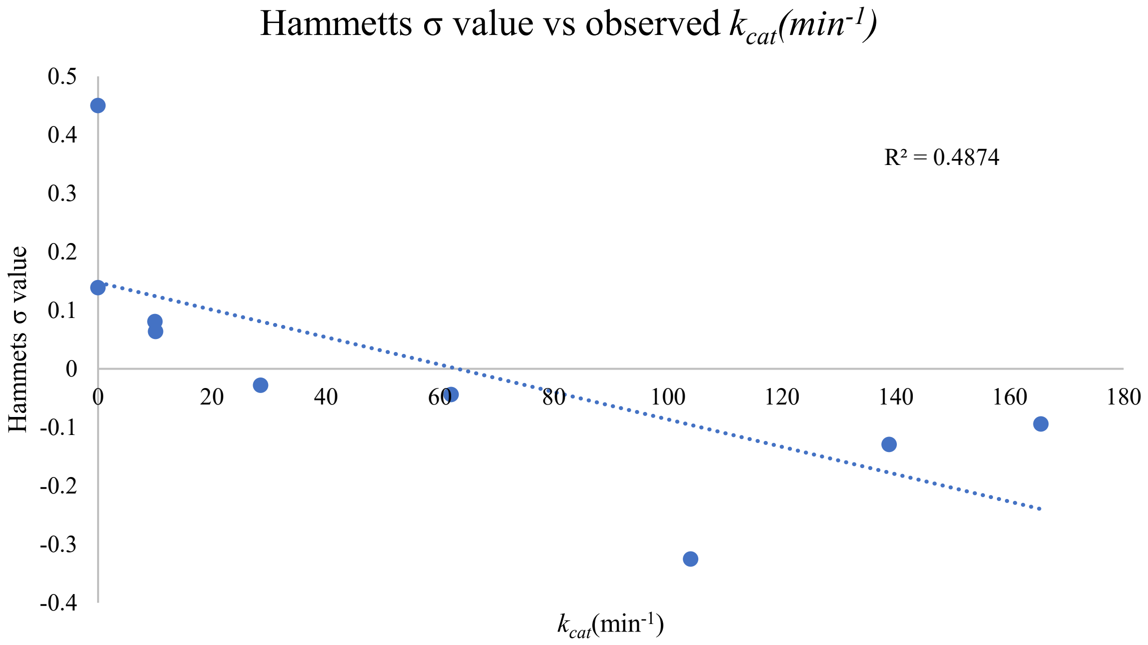

Figure S36. Predicted Hammetts σ values versus observed k_{cat}(min^{-1}) for all non-fluorinated substrates tested. Hammetts σ value predictions were made using the Ertl-molecular Hammett Sigma Constant calculator.

Supplementary Tables

Table S1. *Priestia* megaterium tyrosinase PDB files used for building homology models of the *Ralstonia pseudosolanacearum* tyrosinase. Details included – metal ion co-factors bound in the catalytic centre, substrates/inhibitors bound and crystal structure resolution (Å).

File name	Co-factors bound	Substrate / Inhibitor bound	Resolution
3nm8.pdb	2 x Cu	N/A	2.00 Å
3npy.pdb	2 x Cu	N/A	2.19 Å
3nq0.pdb	1 x Cu	N/A	2.20 Å
3nq1.pdb	2 x Cu	Kojic acid (Inhibitor)	2.30 Å
3ntm.pdb	2 x Cu	N/A	2.30 Å
4p6r.pdb	2 x Zn	Tyrosine	2.20 Å
4p6s.pdb	2 x Zn	L-DOPA	2.20 Å
4p6t.pdb	2 x Cu	Tyrosol	2.50 Å
5i38.pdb	2 x Cu	Kojic acid (Inhibitor)	2.60 Å

5i3a.pdb	2 x Zn	Hydroquinone (Inhibitor)	2.20 Å
5i3b.pdb	2 x Zn	Hydroquinone (Inhibitor)	2.20 Å
5oae.pdb	2 x Cu	SVF[1] (Inhibitor)	2.70 Å
6ei4.pdb	2 x Cu	B5N[2] (Inhibitor)	2.00 Å
6qxd.pdb	2 x Cu	JKB[3] (Inhibitor)	2.32Å

SVF[1] = 1-[4-[(4-fluorophenyl)methyl]piperidin-1-yl]ethenone, B5N[2] = [4-[(4-fluorophenyl)methyl]piperazin-1-yl]-(2-methylphenyl)methanone, JKB[3] = (2,4-dinitrophenyl)-[4-[(4-fluorophenyl)methyl]piperazin-1-yl]methanone

Table S2. A list of *R. pseudosolanacearum* tyrosinase models used in this study. For SWISS-MODEL homology models, only structures containing two copper ions in the catalytic centre were used. All .pdb files listed are freely available on Zenodo.

**File name**	**Model Generation method**	**Template (if used)**	**Residues covered**
7xio-II.pdb	7xio.pdb x-ray crystallography file modified by using SWISS-MODEL to generate missing residues and copper co-factor generation by MIB2 webserver.	7xio.pdb	33-496
7xio_catalytic.pdb	The above structure with the C-terminal domain removed.	7xio.pdb	35-310
RsTyr_AF3.pdb	AlphaFold 3 model of RsTyr containing two copper ions.	N/A	1-496
RsTyr_catalytic_AF3.pdb	The above AlphaFold 3 model with the signal peptide (residues 1 – 32) and C-terminal domain (residues 311 – 496) removed.	N/A	33-310
RsTyr_3nm8.pdb	SWISS-MODEL	3nm8.pdb	36-312
RsTyr_3npy.pdb	SWISS-MODEL	3npy.pdb	37-312
RsTyr_3nq1.pdb	SWISS-MODEL	3nq1.pdb	36-310
RsTyr_3ntm.pdb	SWISS-MODEL	3ntm.pdb	38-312
RsTyr_4p6t.pdb	SWISS-MODEL	4p6t.pdb	36-313
RsTyr_5i38.pdb	SWISS-MODEL	5i38.pdb	37-313
RsTyr_5oae.pdb	SWISS-MODEL	5oae.pdb	36-313
RsTyr_6ei4.pdb	SWISS-MODEL	6ei4.pdb	36-314
RsTyr_6qxd.pdb	SWISS-MODEL	6qxd.pdb	36-312

Table S3. A list of *P. megaterium* tyrosinase GOLD docking PDB files. All .pdb files listed are freely available on Zenodo.

**Substrate**	**Crystal structure with substrate**	**Docked substrate file**
Tyrosine	4p6r.pdb	PmTyr_Tyrosine.pdb
Tyrosol	4p6t.pdb	PmTyr_Tyrosol.pdb

Table S4. A list of *R. pseudosolanacearum* tyrosinase GOLD docking PDB files. All .pdb files listed are freely available on Zenodo.

	**Model ID**				
**Substrate**	**7xio full length**	**7xio catalytic**	**SWISS-MODEL**	**AlphaFold 3 full length**	**AlphaFold 3 catalytic**
**Tyrosine**	Tyrosine_7xio-II.pdb	Tyrosine_7xio_catalytic.pdb	Tyrosine_SM.pdb	Tyrosine_AF3.pdb	Tyrosine_AF3_catalytic.pdb
**Octopamine**	N/A	N/A	Octopamine_SM.pdb	N/A	Octopamine_AF3_catalytic.pdb
**Tyramine**	N/A	N/A	Tyramine_SM.pdb	N/A	Tyramine_AF3_catalytic.pdb
**Tyrosol**	Tyrosol_7xio-II.pdb	Tyrosol_7xio_catalytic.pdb	Tyrosol_SM.pdb	Tyrosine_AF3.pdb	Tyrosol_AF3_catalytic.pdb
**Resveratrol**	N/A	N/A	Resveratrol_SM.pdb	N/A	Resveratrol_AF3_catalytic.pdb
**4-hydroxybenzoic acid**	N/A	N/A	4HBA_SM.pdb	N/A	4HBA_AF3_catalytic.pdb
**4-hydroxymandelic acid**	N/A	N/A	4HMA_SM.pdb	N/A	4HMA_AF3_catalytic.pdb
**4-hydroxyphenylacetic acid**	N/A	N/A	4HPAA_SM.pdb	N/A	4HPAA_AF3_catalytic.pdb
**Phenol**	N/A	N/A	Phenol_SM.pdb	N/A	Phenol_AF3_catalytic.pdb
**2-fluorophenol**	N/A	N/A	2FP_SM.pdb	N/A	2FP_AF3_catalytic.pdb
**3-fluorophenol**	N/A	N/A	3FP_SM.pdb	N/A	3FP_AF3_catalytic.pdb
**4-methoxyphenylacetic acid**	N/A	N/A	4MPAA_SM.pdb	N/A	4MPAA_AF3_catalytic.pdb

Table S5. An overview of *in silico* GOLD docking scores (ChemPLP fitness) of a range of substrates into RsTyr models.

Substrate	7XIO (full length)	AlphaFold 3 catalytic domain model	SWISS-MODEL homology models
L-tyrosine	- 56.9332	57.32	48.625
Tyrosol	-55.673	54.944	42.854
Tyramine	N/A	52.656	45.178
Octopamine	N/A	58.516	45.548
Phenol	N/A	45.347	31.75
2-fluorophenol	N/A	40.553	31.126
3-fluorophenol	N/A	48.54	32.921
Resveratrol	N/A	51.489	47.06
4-hydroxybenzoic acid	N/A	48.45	39.122
4-hydroxymandelic acid	N/A	58.255	45.58
4-hydroxyphenylacetic acid	N/A	54.642	42.873
4-methoxyphenylacetic acid	N/A	46.169	40.7

Table S6. Hammet σ constant prediction for all non-fluorinated substrates tested in this study. Predictions were generated through the Ertl-molecular Hammett Sigma Constant calculator. Hammet σ constant is an estimation of the electron withdrawing or donating potential of benzoic acid derivatives with meta and para substituents.

Substrate	Hammet σ constant (prediction)
Tyrosine	-0.044
Tyrosol	-0.094
Resveratrol	-0.129
Tyramine	-0.028
Octopamine	0.064
Phenol	-0.325
4-hydroxyphenylacetic acid	0.081
4-hydroxymandelic acid	0.139
4-hydroxybenzoic acid	0.523

Table S7. A comparison of K_m, k_{cat} and k_{cat}/K_m of various tyrosinase enzymes towards tyrosine, tyrosol, resveratrol, tyramine, phenol and 4-hydroxybenzoic acid. Tyrosinase abbreviations: **CanSTyr** = *Candidatus sulfopaludibacter* tyrosinase, **CabSTyr** = *Caballeronia* sp. SBC1 tyrosinase, **SinATyr** = *S. acidiphila* ATCC BAA-1392 tyrosinase, **PseSTyr** = *Pseudomonas* sp. MWU12-2323 tyrosinase, **ChrSTyr** = *Chromobacterium sphagni* tyrosinase, **PaPPO** = *Prunus armeniaca* tyrosinase, **OeTyr** = *Olea europea* tyrosinase, **ScTyr** = *Stretomyces avermitilis* MA4680 tyrosinase, **AbPPO1/4** = *Agaricus bisporus* polyphenol oxidase (tyrosinase) 1/4, **MdPPO1/2/3** = *Malus domestica* polyphenol oxidase 1/2/3, **JrPPO1** = *Julans regia* polyphenol oxidase 1, **DlPPO1** = *Dimocarpus longan* polyphenol oxidase 1, **SzTyr** = *Streptomyces* sp. ZL-24 tyrosinase.

	**Tyrosine**			
	Km	**Kcat**	**Kcat/Km**	**Reference**
RsTyr	0.68 ± 0.13	62 ± 14.92	91.1	This study
PmTyr	0.038 ± 0.005	1.29	34.05	[46]
AbPPO1	3.9 ± 0.41	23.2 ± 0.66	5.94	[53]
AbPPO4	3.14 ± 0.33	3.77 ± 0.36	1.20	[54]
JrPPO1	1.42 ± 0.37	6.0 ± 1.1	4.3	[48]

SzTyr	0.6 ± 0.074	4.8 ± 0.23	7.9	[60]
DlPPO1	No activity	No activity	No activity	[55]
CanSTyr	3.28 ± 0.91	6.22 ± 1.37	1.89	[56]
CabSTyr	1.03 ± 0.23	7.35 ± 0.79	7.14	[56]
SinATyr	0.236 ± 0.047	5.55 ± 0.3	23.52	[56]
PseSTyr	0.125 ± 0.14	12.9 ± 0.6	103.2	[56]
ChrSTyr	0.613 ± 0.159	7.36 ± 0.7	12.00	[56]
MdPPO1	No activity	No activity	No activity	[57]
MdPPO2	7.8 ± 1.5	0.92 ± 0.19	0.117	[57]
MdPPO3	9.4 ± 2.1	0.64 ± 0.12	0.068	[57]
ScTyr	0.589 ± 0.0563	1.05 ± 0.037	1.79	[61]
		**Tyrosol**		**Reference**
	Km	**Kcat**	**Kcat/Km**	**Reference**
RsTyr	0.83 ± 0.14	165.5 ± 29.7	200.32	This study
OeTyr	2.23 ± 0.04	0.997 ± 0005	0.448	[58]
		**Resveratrol**		
	Km	**Kcat**	**Kcat/Km**	**Reference**
RsTyr	0.497 ± 0.11	138.83 ± 24.11	279.26	This study
ScTyr	0.0949 ± 0.0139	1.98 ± 0.119	20.9	[61]
		**Tyramine**		
	Km	**Kcat**	**Kcat/Km**	**Reference**
RsTyr	7.14 ± 1.25	28.55 ± 12.25	4	This study
AbPPO1	1.05 ± 0.052	23.2 ± 0.66	22.095	[53]
AbPPO4	9.53 ± 0.71	15.6 ± 0.96	1.63	[54]
JrPPO1	0.451 ± 0.084	24.7 ± 1.5	55	[48]
SzTyr	5.6 ± 0.9	6.3 ± 0.27	1.1 ± 0.19	[60]
DlPPO1	4.3 ± 0.42	35 ± 1.9	8.2	[55]
CanSTyr	0.8 ± 0.167	19.3 ± 1.4	24.125	[56]
CabSTyr	4.23 ± 1.14	6.62 ± 0.62	1.56	[56]
SinATyr	0.989 ± 0.214	0.146 ± 0.011	0.147	[56]
PseSTyr	2.81 ± 0.93	36.2 ± 5.5	12.88	[56]
ChrSTyr	0.376 ± 0.062	8.03 ± 0.51	21.35	[56]
PaPPO	4.48 ± 0.36	1.42 ± 0.13	0.318	[59]
OeTyr	0.88 ± 0.14	0.093 ± 0.005	0.107	[58]
MdPPO1	0.71 ± 0.036	9.5 ± 0.43	13.38	[57]
MdPPO2	5.6 ± 1.5	0.92 ± 0.19	0.164	[57]
MdPPO3	2.4 ± 0.47	1.0 ± 0.71	0.416	[57]

[redacted]				
	Phenol			
	Km	**Kcat**	**Kcat/Km**	**Reference**
RsTyr	2.13 ± 0.07	104.03 ± 21.39	48.84	This study
AbPPO4	12.5 ± 0.41	11.3 ± 0.71	0.904	[54]
[redacted]				
	**4-hydroxybenzoic acid**			
	Km	**Kcat**	**Kcat/Km**	**Reference**
RsTyr	Residual activity	Residual activity	Residual activity	This study
CanSTyr	No activity	No activity	No activity	[56]
CabSTyr	No activity	No activity	No activity	[56]
SinATyr	No activity	No activity	No activity	[56]
PseSTyr	0.454 ± 0.163	2.3 ± 0.28	5.066	[56]
ChrSTyr	4.46 ± 0.52	3.32 ± 0.16	0.744	[56]
